# AI-Powered MRI Radiomics and Deep Learning for Preoperative Prediction of Cavernous Sinus Invasion in Pituitary Adenomas: A Clinically Oriented Review of Current Evidence

**DOI:** 10.1016/j.ynirp.2026.100354

**Published:** 2026-06-02

**Authors:** Farzan Asadirad, Mohammad Rezaei, Parna Ghannadikhosh, Hadi Salehpour, Alireza Motamedi, Mobin Mobadersani, Niloofar soleimannezhad, Sevil Ghaffarzadeh Rad, Esmaeil Gharepapagh, Sahar Rezaei, Mahsa Karbasi, Hossein Arabi

**Affiliations:** a Student Research Committee, Tabriz University of Medical Sciences, Tabriz, Iran; b Clinical Research Development Unit of Tabriz Valiasr Hospital, Tabriz University of Medical Sciences, Tabriz, Iran; c Endocrine Research Center Tabriz University of Medical Sciences, Tabriz, Iran; d Department of Nuclear Medicine, Medical School, Tabriz University of Medical Sciences, Tabriz, Iran; e Department of Radiology, Medical School, Tabriz University of Medical Sciences, Tabriz, Iran; f Division of Nuclear Medicine and Molecular Imaging, Geneva University Hospital, Geneva 4, CH-1211, Switzerland

**Keywords:** Pituitary adenoma, Cavernous sinus invasion, Radiomics, Machine learning, Deep learning

## Abstract

**Background:**

Pituitary adenomas represent one of the most common intracranial tumors, and cavernous sinus invasion (CSI remains a major challenge for surgical management. Although the Knosp grading system provides a widely used radiological framework, its subjective nature and inter-observer variability limit diagnostic reliability. In recent years, advanced computational methods have been investigated to improve the preoperative prediction of invasion.

**Objective:**

This review synthesizes current evidence on the use of radiomics, machine learning (ML), and deep learning (DL) approaches in the detection and assessment of CSI in pituitary adenomas, with particular emphasis on their comparative performance against traditional imaging methods.

**Methods:**

Studies employing MRI-based radiomic feature extraction, ML classifiers, and convolutional neural networks were analyzed. Reported models commonly incorporated intensity, texture, and shape descriptors, or applied end-to-end DL architectures for automated prediction. Performance metrics such as accuracy, sensitivity, specificity, AUC, and Dice similarity coefficients were compared across studies, with Knosp grade serving as a frequent benchmark.

**Results:**

Evidence suggests that ML and DL models consistently outperform conventional MRI interpretation in predicting CSI. Radiomics pipelines integrating quantitative imaging features with clinical variables achieved high diagnostic accuracy, while CNN-based models trained on contrast-enhanced MRI often exceeded AUC values of 0.85. Furthermore, automated segmentation frameworks demonstrated reliable delineation of tumor boundaries, facilitating improved assessment of invasive behavior. Despite promising outcomes, limitations such as small sample sizes, single-center designs, and lack of external validation restrict broad clinical adoption.

**Conclusions:**

Radiomics and AI-driven approaches show substantial potential for enhancing preoperative evaluation of pituitary adenomas with CSI. Standardized imaging protocols, multicenter collaborations, and transparent model validation are essential for future integration into neurosurgical decision-making.

## Highlights

• ML/DL models outperform traditional MRI in CSI prediction for pituitary adenomas, with AUC often >0.85.
• Radiomics pipelines integrating MRI features and clinical variables achieve high diagnostic accuracy.
• CNN-based segmentation reliably delineates tumor boundaries, aiding invasive behavior assessment.
• Limitations: Small sample sizes and lack of external validation hinder clinical adoption.
• Future needs: Multicenter studies, standardized imaging, and transparent model validation.

## 1 Introduction

Pituitary adenomas (PAs) also known as pituitary neuroendocrine tumor are among the most common intracranial neoplasms, accounting for roughly 10–15% of primary brain tumors (Di Ieva et al., 2014; Zheng et al., 2024). They originate from pituitary adenohypophyseal cells and can be classified into functionating group-producing excessive hormones- and non-functioning group. PAs can also be categorized as macroadenomas (≥10 mm) or microadenomas (<10 mm) (Tritos and Miller, 2023). PAs are often considered as nonmalignant but the heavy burden on patients and health care systems are inevitable (Collaborators, G. B. D. Causes of Death, 2025; Daly and Beckers, 2020; Disease, G. B. D., Injury, and Collaborators Risk Factor, 2025).

While generally benign, a significant subset exhibits invasive behavior like rapid growth, higher recurrence, enlargement and encroaching on surrounding structures such as the sphenoid sinus, diaphragma sellae, and cavernous sinus (Di Ieva et al.)- a region housing critical neurovascular structures such as the internal carotid artery and cranial nerves III, IV, V, and VI (Buchfelder, 2009; Di Ieva et al., 2014). This invasion may happen infrasellar, suprasellar or parasellar. Studies suggest that parasellar invasion to CS takes place in six to forty percent of cases (Ahmadi et al., 1986; Ajlan et al., 2017; Fahlbusch and Buchfelder, 1988; Sol et al., 2014; Trouillas et al., 2013; Vieira et al., 2006).Cavernous sinus invasion (CSI) is clinically important because it greatly complicates complete tumor resection and increases morbidity and mortality of surgeries (Cb, 1977; Cottier et al., 2000). CSI can be either a true invasion or an opportunistic event because of the weak wall of the CS (Dhandapani et al., 2016).

CSI has been identified as a major risk factor for incomplete surgical removal of the adenoma which can lead to failure to achieve endocrinological remission, and a higher rate of tumor recurrence. It also causes an increase in the need for adjuvant to control residual disease. The intimate involvement of critical neurovascular structures in the cavernous sinus not only limits surgical accessibility but also raises the stakes for operative risk. Thus, reliably determining whether a pituitary adenoma has invaded the CS *preoperatively* is pivotal for neurosurgical planning and prognostication (Lefevre et al., 2024).

Magnetic resonance imaging (MRI) is the basis of preoperative evaluation for pituitary tumors and typically provides the first hints of invasive growth. One of the first classifications for invasive pituitary adenomas was the Hardy classification which puts them in two categories of grade III (focal bone erosion) or grade IV(extensive bone erosion including extrasellar structures (Hardy, 1969). In current practice, radiologists and surgeons often measure cavernous sinus involvement using MRI-based grading systems, most famously the Knosp classification. Introduced in 1993 and only modestly modified since (splitting grade 3 into 3A and 3B), the Knosp scale assigns grades 0 through 4 based on the tumor's extension relative to tangents drawn through the intracavernous carotid arteries (Rouf et al., 2024). Higher grades generally correlate with a greater likelihood of CSI.

However, in clinical settings, there are notable limitations to traditional imaging evaluations. Conventional MRI interpretations are quite subjective with significant inter-observer variability and they are often limited in distinguishing subtle invasions (Zheng et al., 2024). Some radiologic criteria are not accepted globally (Dhandapani et al., 2016). Furthermore, different observers can disagree on the exact grade when applying the full Knosp scale.

Recently, tools like machine learning (ML) and deep learning (DL) have emerged for analyzing medical imaging data and offer data-driven approaches to enhance diagnostic accuracy and help in outcome prediction and disease management (Arabi and Zaidi, 2020; Dadgar et al., 2025; Rezaei et al., 2025; Varoquaux and Cheplygina, 2022). ML models have shown the ability to analyze high-dimensional imaging features (often referred to as radiomics) to predict tumor behavior such as invasiveness (Hasanabadi et al., 2024; Safarian et al., 2025). Radiomics involves the extraction of quantitative descriptors from imaging data which reveals tumor characteristics including texture, shape, intensity, and spatial relationships that may be undetectable to the human eye (Kumar et al., 2012; Zheng et al., 2024). Deep learning, particularly convolutional neural networks (CNNs), further advances this capability by learning hierarchical features directly from raw imaging data (Yamashita et al., 2018).

Studies suggest encouraging early results from applying ML/DL models to this problem. Radiomics-based models have shown the ability to outperform conventional MRI assessment in predicting CSI. In one study, Niu et al. used a radiomics pipeline on contrast-enhanced T1 and T2 MRI sequences to predict cavernous sinus invasion preoperatively in pituitary adenomas (Niu et al., 2019). Their model combined three selected quantitative imaging features with clinical factors and achieved an area under the ROC curve (AUC) of about 0.87 on a validation set. This was a substantial improvement over the performance of Knosp grading alone and highlighted the value of high-dimensional image features in capturing invasion risk. Deep learning methods have shown similar notable performance. Fang et al. constructed a CNN model (based on a ResNet-50 architecture with transfer learning) trained on T1-weighted pituitary MRI scans from over 370 patients with surgically confirmed invasion status (Fang et al., 2022). The CNN achieved accuracies ranging from 80 to 96% in distinguishing invasive from non-invasive tumors, with cross-validated AUCs between 0.89 and 0.98.

The integration of these AI-based tools into clinical settings holds substantial promise. More accurate preoperative detection of CSI may help neurosurgeons in tailoring surgical strategies more effectively and reduce intraoperative unexpected events, and inform patients more clearly about expected outcomes and potential complications. Additionally, identifying invasive adenomas early may help schedule better follow-up plans and consideration of adjunct therapies. Given the growing interest and rapidly evolving landscape, we aimed to systematically synthesize current evidence on the use of ML and DL in the screening and diagnosis of cavernous sinus invasion in pituitary adenomas.

## 2 Methods

This study was designed and conducted as a systematic review and meta-analysis to synthesize evidence on AI applications for cavernous sinus invasion (CSI) detection. The methodology and reporting adhere to the PRISMA 2020 (Preferred Reporting Items for Systematic Reviews and Meta-Analyses) statement to ensure rigor and transparency.

### 2.1 Registration and protocol

In accordance with PRISMA 2020 Item 24, we declare that this systematic review was not prospectively registered in an international database (such as PROSPERO), and a formal external protocol was not prepared prior to the study. However, the review process, including search strategy, study selection, and data extraction, was strictly governed by an internal predefined framework to maintain consistency and minimize reporting bias.

### 2.2 Search strategy and literature identification

A structured literature search was conducted to identify original research articles evaluating the application of machine learning (ML) and deep learning (DL) methods for the detection, diagnosis, or prediction of cavernous sinus invasion (CSI) in patients with pituitary adenomas. Searches were carried out in PubMed, Embase, Web of Science, and Scopus databases from inception to April 2025. The search strategy combined controlled vocabulary and free-text terms including “pituitary adenoma,” “cavernous sinus invasion,” “machine learning,” “deep learning,” “artificial intelligence,” “radiomics,” “convolutional neural network,” “predictive modeling,” and “MRI.”

Search results were screened for eligibility in two stages. In the first stage, titles and abstracts were reviewed to exclude articles clearly unrelated to the topic. In the second stage, full texts were retrieved and assessed against inclusion criteria. Additional studies were identified by manually screening the reference lists of included papers and relevant reviews (Supplementary Table 1).

### 2.3 Inclusion and exclusion criteria

Studies were included if they satisfied all of the following criteria:

(1) employed ML or DL algorithms to develop or validate predictive or diagnostic models for CSI in pituitary adenomas; (2) used original datasets derived from imaging (MRI or radiomics), intraoperative findings, or clinical and pathological records; (3) reported quantitative performance metrics such as accuracy, AUC (area under the curve), sensitivity, specificity, or Dice coefficient; and (4) provided sufficient methodological details to permit reproducibility or quality assessment.

**Fig. 1:**
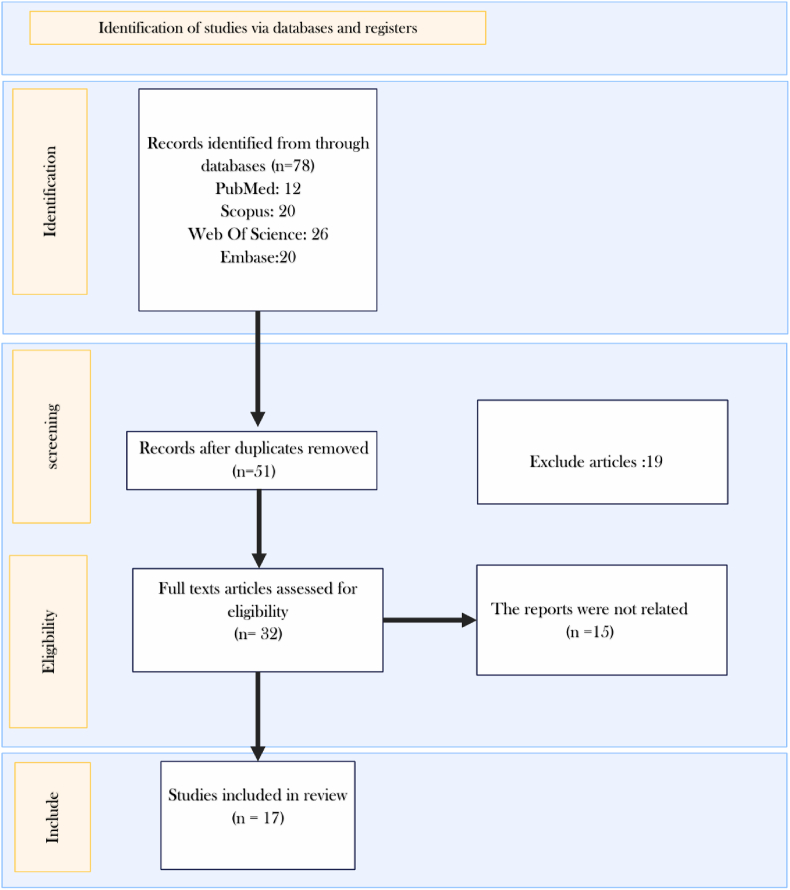
PRISMA flow diagram illustrating the selection process of included studies.

Exclusion criteria comprised: (1) reviews or meta-analyses without new data, (2) conference abstracts lacking full text, (3) letters or editorials, (4) case reports, and (5) studies without validation of model performance. (6) Studies focusing solely on general adenoma classification without specific reference to cavernous sinus invasion were also excluded (Fig. 1).

### 2.4 Data extraction process

Two independent reviewers systematically extracted data from each included study using a pre-piloted data extraction form. Extracted variables included: study design (retrospective or prospective cohort), sample size and cohort characteristics (e.g., age, sex distribution, adenoma size, functioning subtype), imaging protocols (MRI sequences, slice thickness, contrast enhancement), machine learning or deep learning methods used (e.g., CNN architecture, random forest, logistic regression),approach to training, validation, and testing (internal cross-validation, external validation cohorts),model performance metrics (AUC, accuracy, Dice coefficient, sensitivity, specificity), pathological confirmation rates, and Knosp grade distributions and related CSI prevalence.

For studies that subdivided cohorts (e.g., training and validation groups), data were extracted separately to preserve clarity. Any discrepancies in data extraction were resolved by discussion and consensus with a third reviewer.

### 2.5 Quality assessment

The methodological quality of the 17 studies in this systematic review was assessed using a hybrid approach combining validated tools to address diverse study designs, including retrospective and prospective cohort studies, diagnostic accuracy studies, and ML-based prediction models. The Newcastle-Ottawa Scale (NOS) evaluated cohort quality across selection, comparability, and outcome domains. The QUADAS-2 tool assessed diagnostic accuracy studies, examining risk of bias and applicability in patient selection, index test, reference standard, and flow/timing domains. The TRIPOD checklist was applied to ML-based models, focusing on transparency (model specification, validation, performance reporting) and data quality (data source, sample size, missing data handling). The ROBINS-I tool synthesized overall risk of bias, addressing confounding, selection, measurement, and missing data. This multi-tool approach, aligned with the Cochrane Handbook for Systematic Reviews of Interventions (Higgins, 2008) and best practices for heterogeneous systematic reviews (Moons et al., 2015; Whiting et al., 2011), ensured a comprehensive and robust quality evaluation.

Each study was independently scored using standardized criteria, with stricter penalties for single-center designs, lack of external validation, weak reference standards, or unreported missing data handling to enhance differentiation. Overall quality scores (out of 10, rounded to one decimal place) were calculated as weighted averages: for diagnostic studies, NOS contributed 40%, TRIPOD 35%, and QUADAS-2 25%; for non-diagnostic studies, NOS contributed 55% and TRIPOD 45%. QUADAS-2 scores were converted numerically (low risk = 4, moderate = 2, high = 0) based on the highest risk across domains. Missing information (e.g., blinding, follow-up duration) was conservatively scored as moderate risk unless specified. Ethical considerations, such as ethics approval and informed consent, were noted but only influenced scores if linked to methodological flaws (e.g., unaddressed missing data). ROBINS-I summarized risk of bias as low, moderate, high, or critical, integrating findings from NOS, QUADAS-2, TRIPOD, and additional bias sources (e.g., attrition, confounding).

**Fig. 2:**
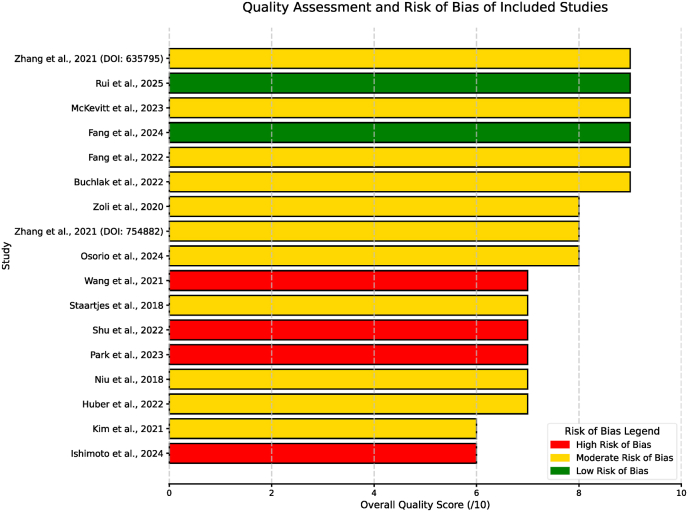
Quality assessment of 17 studies included in the systematic review, showing overall scores (out of 10, rounded to one decimal place) and ROBINS-I risk of bias levels. Scores are derived from weighted averages of NOS (40% for diagnostic, 55% for non-diagnostic), TRIPOD (35% for diagnostic, 45% for non-diagnostic), and QUADAS-2 (25% for diagnostic) scores, with stricter penalties for methodological weaknesses. The figure highlights variability in methodological rigor, with higher scores indicating greater reliability.

Quality assessment results, presented in Fig. 2 and Table 1, show overall scores ranging from 6 to 9 out of 10, reflecting variability in methodological rigor. Strengths included consistent consecutive sampling, clear inclusion/exclusion criteria, and detailed ML reporting. Common limitations were single-center designs, retrospective data collection, and lack of external validation. These findings inform study weighting in meta-analyses or narrative syntheses, with higher-scoring studies exerting greater influence (Fang et al., 2024; Rui et al., 2025). The hybrid approach, supported by Cochrane guidance (Higgins, 2008), ensures transparent and reliable quality assessment, facilitating evidence interpretation for preoperative cavernous sinus invasion detection, postoperative outcome prediction, and quality of life assessment in pituitary adenoma patients.

**Table 1:** Quality Assessment of Included Studies Using a Hybrid Approach (NOS, QUADAS-2, TRIPOD, ROBINS-I). This table summarizes the quality assessment of 17 studies, utilizing NOS for cohort quality, QUADAS-2 for diagnostic accuracy, TRIPOD for ML model transparency and data quality, and ROBINS-I for overall risk of bias. Overall scores (out of 10, rounded to one decimal place) are calculated as weighted averages: diagnostic studies (NOS 40%, TRIPOD 35%, QUADAS-2 25%); non-diagnostic studies (NOS 55%, TRIPOD 45%). Stricter criteria penalize methodological weaknesses, with comments highlighting key strengths and limitations.

Article	Study Design	NOS Score (/9)				QUADAS-2 (Risk of Bias/Applicability)				TRIPOD Score (/8)			Overall Score (/10) (Rounded)	ROBINS-I Risk of Bias	Comments
		Selection (/4)	Comparability (/2)	Outcome (/3)	Overall (/9)	Patient Selection	Index Test	Reference Standard	Flow/Timing	Transparency (/4)	Quality (/4)	Overall (/8)			
Kim et al., 2021**;**Kim et al. 2021**)**	Retrospective cohort	3/4 (single-center, selection bias risk)	1/2 (no explicit confounder control)	2/3 (only 4% surgical confirmation)	6/9	Moderate (single-center, retrospective)	Low (blinded neuroradiologists)	Moderate (clinical-radiologic consensus)	Low (no attrition)	3/4 (limited DLR algorithm details)	2/4 (no external validation, small sample)	5/8	6	Moderate	Strong DLR diagnostics, but single-center and weak surgical confirmation limit generalizability.
Buchlak et al., 2022**;**Buchlak et al. 2022**)**	Prospective cohort	4/4 (multicenter, representative)	2/2 (confounders controlled via regression)	2/3 (56% attrition)	8/9	N/A	N/A	N/A	N/A	4/4 (detailed ML methods)	3/4 (no external validation)	7/8	9	Moderate	Robust prospective multicenter design, but high attrition reduces reliability. Clear ML reporting.
(2022)**;**Fang et al. 2022**)**	Retrospective cohort	4/4 (multicenter, clear criteria)	1/2 (limited confounder control beyond Knosp)	3/3 (surgical confirmation)	8/9	Low (multicenter, clear criteria)	Low (blinded CNN evaluation)	Low (surgical confirmation)	Low (no attrition)	3/4 (sparse Resnet50 details)	3/4 (no external validation)	6/8	9	Moderate	High-quality multicenter study with surgical confirmation, but retrospective, no external validation.
(2022)**;**Huber et al. 2022**)**	Retrospective cohort	3/4 (single-center, small sample)	1/2 (imputation bias risk)	2/3 (variable follow-up duration)	6/9	N/A	N/A	N/A	N/A	3/4 (clear methods, predictors)	3/4 (no external validation)	6/8	7	Moderate	Comprehensive ML, but small single-center sample and imputation bias constrain findings.
(2023)**;**Park et al. 2023**)**	Retrospective cohort	3/4 (single-center, high exclusions: 78%)	2/2 (standardized imaging protocols)	2/3 (4.7% surgical confirmation)	7/9	Moderate (single-center, exclusions)	Low (blinded readers)	Moderate (clinical consensus)	Moderate (high exclusions)	3/4 (limited DLR details)	2/4 (no external validation)	5/8	7	High	DLR enhances visualization, but high exclusion rate and single-center design weaken reliability.
(2021)**;**Wang et al. 2021**)**	Retrospective cohort	3/4 (single-center, high Knosp prevalence)	2/2 (random data split)	2/3 (manual segmentation bias)	7/9	Moderate (single-center)	Moderate (manual segmentation)	Low (surgical consistency confirmation)	Low (no attrition)	3/4 (clear GSU-Net details)	3/4 (no external validation)	6/8	7	High	Strong segmentation, but single-center and high Knosp prevalence limit generalizability.
(2020)**;**Zoli et al. 2020**)**	Retrospective cohort	3/4 (single-center, small sample)	2/2 (multivariable predictors)	2/3 (missing cortisol data)	7/9	N/A	N/A	N/A	N/A	3/4 (clear ML methods)	3/4 (no external validation)	6/8	8	Moderate	Robust dataset, but single-center and missing data reduce applicability.
Zhang et al., 2021a**;**Zhang et al. 2021b**)**	Retrospective cohort	4/4 (large sample, clear criteria)	2/2 (univariate regression control)	2/3 (no long-term follow-up)	8/9	N/A	N/A	N/A	N/A	4/4 (online calculator, clear methods)	3/4 (no external validation)	7/8	9	Moderate	Large sample and robust ML, but single-center design limits generalizability.
Zhang et al., 2021a**;**Zhang et al. 2021a**)**	Retrospective cohort	3/4 (single-center, single surgeon)	2/2 (multivariate analysis)	2/3 (no long-term follow-up)	7/9	N/A	N/A	N/A	N/A	4/4 (novel EMR approach, clear)	3/4 (no external validation)	7/8	8	Moderate	Innovative EMR use, but single-surgeon bias and single-center design noted.
(2018)**;**Staartjes et al. 2018**)**	Retrospective cohort	3/4 (single-center, moderate sample)	2/2 (multiple predictors)	2/3 (no long-term follow-up)	7/9	Moderate (single-center)	Low (standardized DNN)	Low (MRI-based GTR)	Low (no attrition)	3/4 (clear DNN methods)	3/4 (no holdout set)	6/8	7	Moderate	Early deep learning application, but small sample and single-center limit impact.
Niu et al., 2019**;**Niu et al. 2019**)**	Retrospective cohort	3/4 (single-center, Knosp 2/3 focus)	2/2 (multivariate regression)	2/3 (manual segmentation bias)	7/9	Moderate (single-center)	Moderate (manual segmentation)	Low (surgical confirmation)	Low (no attrition)	3/4 (clear radiomics pipeline)	3/4 (no external validation)	6/8	7	Moderate	Robust radiomics, but manual segmentation and single-center design reduce generalizability.
(2023)**;**McKevitt et al. 2023**)**	Retrospective cohort	4/4 (multicenter, clear criteria)	2/2 (multivariate control)	2/3 (assay variability)	8/9	N/A	N/A	N/A	N/A	4/4 (clear Pit-SCHEME, methods)	3/4 (no external validation)	7/8	9	Moderate	Strong multicenter design, but retrospective nature and assay variability noted.
(2024)**;**Fang et al. 2024**)**	Retrospective cohort	4/4 (multicenter, large sample)	2/2 (univariate control)	2/3 (2D image limitation)	8/9	Low (multicenter, clear criteria)	Low (blinded CNN)	Low (surgical confirmation)	Low (no attrition)	4/4 (clear methods, external validation)	3/4 (2D image limitation)	7/8	9	Low	High-quality multicenter study with external validation, minor 2D image limitation.
(2024)**;**Ishimoto et al. 2024**)**	Retrospective cohort	2/4 (small sample, single-center)	2/2 (standardized protocols)	1/3 (no surgical confirmation in 6/24)	5/9	Moderate (small sample, exclusions)	Low (blinded readers)	Moderate (clinical consensus)	Moderate (40.4% exclusions)	3/4 (limited DLR details)	2/4 (small sample, no validation)	5/8	6	High	Innovative DLR, but very small sample and high exclusions limit reliability.
(2022)**;**Shu et al. 2022**)**	Retrospective cohort	3/4 (single-center, small test set)	2/2 (balanced groups)	2/3 (small clinical test sample)	7/9	Moderate (single-center)	Low (blinded segmentation)	Low (surgical confirmation)	Moderate (small test set)	3/4 (clear U-Net details)	3/4 (no external validation)	6/8	7	High	Promising DL for Ki67LI, but small dataset and single-center design constrain findings.
Osorio et al., 2025**;**Osorio et al. 2025**)**	Retrospective cohort	3/4 (single-center, 38.8% LTFU)	2/2 (univariable regression)	2/3 (missing GH/IGF-1 data)	7/9	N/A	N/A	N/A	N/A	3/4 (clear random forest methods)	3/4 (no external validation)	6/8	8	Moderate	Robust predictors, but high LTFU and single-center design reduce reliability.
(2025)**;**Rui et al. 2025**)**	Retrospective cohort	4/4 (multicenter, large sample)	2/2 (univariate control)	2/3 (single-sequence limitation)	8/9	Low (multicenter, clear criteria)	Low (blinded segmentation)	Low (surgical confirmation)	Low (no attrition)	4/4 (clear MTMAU-Net, validation)	3/4 (single-sequence limitation)	7/8	9	Low	High-quality multicenter study with external validation, minor single-sequence limitation.

## 3 Results

### 3.1 Statistical analyses and synthesis

Given the diversity of methods and outcomes across studies, formal meta-analysis was not feasible. Instead, a structured narrative synthesis was performed. Descriptive statistics summarized cohort demographics, imaging characteristics, ML/DL architectures, and performance metrics. Studies were tabulated to compare design (e.g., single-center vs. multicenter), model types (CNN, random forest, logistic regression, multitask U-Net), sample sizes, validation approaches (internal cross-validation vs. external testing), and key performance indices (e.g., AUC ranging from 0.743 to 1.00, Dice coefficients for segmentation up to 0.940). Visual summaries, including figures depicting log10 sample size distributions, sex proportions, and Knosp grade distributions, were used to illustrate variability. Where studies reported statistical comparisons of model performance to conventional methods (e.g., Knosp grading), p-values were extracted to contextualize improvements.

### 3.2 Characterizing cohort scale and structure in pituitary adenoma studies

The scale, composition, and organization of participant cohorts are pivotal in determining the robustness and generalizability of ML and DL analyses in pituitary adenoma studies. Cohort sizes across the reviewed studies ranged significantly, from a minimum of 24 patients to a maximum of 1,045, with diverse subgroup classifications based on tumor characteristics, treatment phases, or analytical requirements. For example, some studies categorized patients by tumor invasiveness (e.g., invasive vs. non-invasive), adenoma size (e.g., microadenomas vs. macroadenomas), or pre- and postoperative status. Others focused on specific adenoma types, such as corticotrophic or somatotrophic, or molecular markers like Ki67 expression levels (<3% vs. ≥3%). Data splits for training and testing were common, with some studies employing balanced or multicenter cohorts to enhance model validation. A comprehensive overview of cohort sizes and subgroup details for the 17 studies is provided in Table 2 (Buchlak et al., 2022; Fang, et al. 2022, 2024; Huber et al., 2022; Ishimoto et al., 2024; Kim et al., 2021; McKevitt et al., 2023; Niu et al., 2019; Osorio et al., 2025; Park et al., 2023; Rui et al., 2025; Shu et al., 2022; Staartjes et al., 2018; Wang et al., 2021; Zhang, et al. 2021a, 2021b; Zoli et al., 2020).

**Table 2:** Summary of Sample Sizes and Subgroups. Overview of total sample sizes and subgroup classifications for the 17 studies included in the analysis of pituitary adenoma invasion into the cavernous sinus, sorted by total sample size in descending order to facilitate comparison of participant numbers and subgroup diversity.

Study	Total Sample Size	Log10(Sample Size)	Subgroups
Zhang et al., 2021b; Zhang et al., 2021b)	1045	3.02	Training (n = 836), Test (n = 209)
(2025); Rui et al., 2025)	926	2.97	Training (n = 816), Validation (n = 110)
(2024); Fang et al., 2024)	729	2.86	PUMCH (n = 411), FGH (n = 236), YFPH (n = 44), SAH (n = 24), TPH (n = 14)
(2022); Buchlak et al., 2022)	451	2.65	Preoperative (n = 451), Follow-up at 12 months (n = 199)
Zhang et al., 2021b; Zhang et al., 2021a)	419	2.62	Training (n = 335), Test (n = 84)
(2023); McKevitt et al., 2023)	392	2.59	Corticotrophic adenomas (n = 143), Somatotrophic adenomas (n = 97), Lactotrophic adenomas (n = 96), Mammosomatotrophic adenomas (n = 30), Mixed GH/PRL adenomas (n = 16), Thyrotrophic adenomas (n = 10)
(2022); Fang et al., 2022)	371	2.57	Invasive (n = 102), Non-invasive (n = 269)
(2022); Shu et al., 2022)	362	2.56	Invasion analysis (n = 246; Ki67 < 3%: 135, ≥3%: 111), Clinical test (n = 27)
(2021); Wang et al., 2021)	213	2.33	First group (n = 163; training: 131, test: 32), Second group (n = 50 new, total 170 for consistency)
Niu et al., 2019; Niu et al., 2019)	194	2.29	Training (n = 97), Test (n = 97)
(2020); Zoli et al., 2020)	151	2.18	Microadenomas (n = 80), Macroadenomas (n = 35), MRI-undetectable (n = 36)
(2018); Staartjes et al., 2018)	140	2.15	None
(2023); Park et al., 2023)	104	2.02	Preoperative (n = 40), Postoperative (n = 64)
(2022); Huber et al., 2022)	86	1.93	Macroadenomas (n = 41), Microadenomas (n = 45)
Osorio et al., 2025; Osorio et al., 2025)	80	1.90	Remission (n = 60), Non-remission (n = 20)
(2021); Kim et al., 2021)	65	1.81	Residual tumor (n = 45), No residual tumor (n = 20)
(2024); Ishimoto et al., 2024)	24	1.38	Pre-treatment (n = 4), Post-treatment (n = 20)

**Fig. 3:**
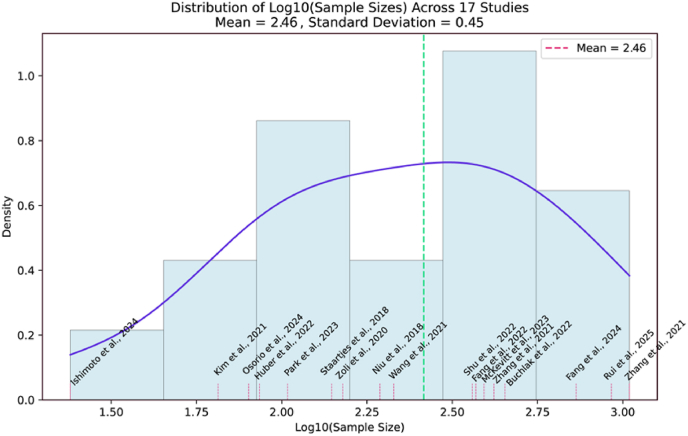
Distribution of Log10(Sample sizes).

From a statistical standpoint, a log10-based distribution of sample sizes across all 17 studies, depicted in Fig. 3, reveals a mean of 2.46 and a standard deviation of 0.45. Excluding the smallest cohort (n = 24) as an outlier, the arithmetic mean of raw sample sizes rises to 419, with a standard deviation of 331. The log10 of this mean, approximately 2.62, slightly exceeds the log-transformed mean across all studies, illustrating the influence of small cohorts on statistical summaries. The median sample size, at 362, indicates a right-skewed distribution, with the first quartile (Q1) at 140 and the third quartile (Q3) at 729. The largest cohort reached 1,045, while the smallest, excluding the outlier, was 65, as detailed in Table 2 (Fang et al., 2024; Ishimoto et al., 2024; Kim et al., 2021; Shu et al., 2022; Staartjes et al., 2018; Zhang et al., 2021b). Variability in subgroup diversity, ranging from no breakdowns in some studies (McKevitt et al., 2023; Staartjes et al., 2018) to up to six adenoma types in others, highlights disparities in cohort design, influencing the robustness and applicability of ML-driven insights.

Histogram with kernel density estimate, depicting the distribution of log10 (sample sizes) across 17 studies on pituitary adenoma invasion. The light blue rectangles represent the frequency of log10 (sample sizes) in discrete bins, while the overlaid smooth blue curve illustrates the kernel density estimate, reflecting the continuous density distribution. A green dashed vertical line marks the mean log10 (sample size) at 2.46 (equivalent to approximately 288 on the original scale), with a standard deviation of 0.45. Each of the 17 studies is labeled at its respective log10 (sample size) position, with red dashed lines connecting the labels to the x-axis for clarity. The x-axis spans approximately 1.3 to 3.1, covering the range of log10 values, and the y-axis represents density, peaking around 0.8.

### 3.3 Characterizing study designs and methodologies in pituitary adenoma research

The exploration of pituitary adenoma invasion through advanced computational approaches, such as ML and DL, predominantly relies on primary research frameworks that harness original data to enhance diagnostic and prognostic capabilities (Buchlak et al., 2022; Fang, et al. 2022, 2024; Huber et al., 2022; Ishimoto et al., 2024; Kim et al., 2021; McKevitt et al., 2023; Niu et al., 2019; Osorio et al., 2025; Park et al., 2023; Rui et al., 2025; Shu et al., 2022; Staartjes et al., 2018; Wang et al., 2021; Zhang, et al. 2021a, 2021b; Zoli et al., 2020). Several investigations prioritize preoperative prediction, utilizing CNNs to identify CSI, thereby optimizing surgical planning, as evidenced in studies published in open-access journals like *Frontiers in Oncology* (Fang, et al. 2022, 2024; Rui et al., 2025). Other efforts focus on postoperative outcomes, employing supervised ML to predict remission rates in CD, biochemical remission in somatotroph adenomas, or QoL improvements following endoscopic skull base surgery, often integrating EMRs to bolster predictive accuracy (Buchlak et al., 2022; Huber et al., 2022; McKevitt et al., 2023; Osorio et al., 2025; Zhang, et al. 2021a, 2021b). Additionally, studies in perioperative settings leverage DL-based image reconstruction to refine radiological evaluations of the pituitary axis and CSI, addressing clinical demands for precise boundary delineation during surgical planning and postoperative assessment (Ishimoto et al., 2024; Kim et al., 2021; Park et al., 2023). While primary research fosters targeted computational advancements, the scarcity of meta-analyses limits broader synthesis, potentially constraining the contextualization of findings across varied clinical settings.

Retrospective cohort designs dominate, with 16 studies utilizing historical patient data to assess clinical outcomes across diverse scenarios (Fang, et al. 2022, 2024; Huber et al., 2022; Ishimoto et al., 2024; Kim et al., 2021; McKevitt et al., 2023; Niu et al., 2019; Osorio et al., 2025; Park et al., 2023; Rui et al., 2025; Shu et al., 2022; Staartjes et al., 2018; Wang et al., 2021; Zhang, et al. 2021a, 2021b; Zoli et al., 2020). Single-center retrospective studies, often conducted at specialized institutions like PUMCH, provide in-depth analyses of conditions such as CD but face moderate risks of bias due to retrospective methodologies, selection biases, and limited external validation, as evaluated by tools like Risk Of Bias In Non-randomized Studies of Interventions (ROBINS-I) (Niu et al., 2019; Zhang, et al. 2021a, 2021b). In contrast, multicenter retrospective cohorts, spanning institutions in regions like China and Switzerland, incorporate diverse patient populations to enhance external validity, though they grapple with challenges such as variability in imaging protocols or surgical techniques (Fang, et al. 2022, 2024; Huber et al., 2022; Rui et al., 2025; Zoli et al., 2020). A notable exception is a prospective multicenter cohort study across three Australian hospitals, which offers a forward-looking perspective by predicting QoL outcomes (Buchlak et al., 2022). The heavy reliance on retrospective data, while resource-efficient, introduces potential biases and restricts causal inference, particularly in single-center studies where ethical considerations, such as waived informed consent, are prevalent, yet discussions on algorithmic fairness remain limited.

### 3.4 Global patterns in pituitary adenoma research: A geographical perspective

**Fig. 4:**
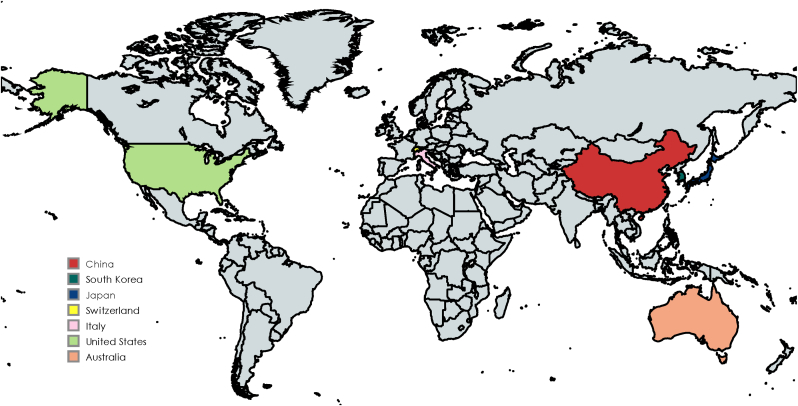
World map highlighting the geographical distribution of pituitary adenoma studies across 7 countries. South Korea (2 studies), Japan (1 study), China (8 studies), Switzerland (3 studies), Italy (1 study), United States (2 studies), and Australia (1 study) are colored distinctly, while others remain in a neutral shade, illustrating the global spread of research efforts. Note: One study (Zoli et al., 2020) is shared between Switzerland and Italy.

Global pituitary adenoma research, as shown in Fig. 4, reveals distinct regional contributions to ML and DL methodologies, with East Asia, led by China, dominating through eight studies from centers like PUMCH, focusing on CNNs and DLR for CSI detection and predictive modeling (Fang, et al. 2022, 2024; Niu et al., 2019; Rui et al., 2025; Shu et al., 2022; Wang et al., 2021; Zhang, et al. 2021a, 2021b). South Korea and Japan contribute two and one studies, respectively, advancing MRI-based DLR (Ishimoto et al., 2024; Kim et al., 2021; Park et al., 2023), while Europe, with three studies from Switzerland and Italy, emphasizes multicenter efforts for surgical outcome prediction (Huber et al., 2022; Staartjes et al., 2018; Zoli et al., 2020). North America's two U.S.-based studies explore predictive modeling and remission under IRB oversight (McKevitt et al., 2023; Osorio et al., 2025), and Australia's single prospective study highlights QoL outcomes (Buchlak et al., 2022). Despite East Asia's computational lead, the predominance of retrospective, single-center studies, moderate bias risks, and inconsistent ethical focus on algorithmic fairness limit global applicability and equitable translation. Future research should prioritize prospective designs, meta-analyses, and ethical considerations to enhance precision and fairness in pituitary adenoma diagnostics and management.

### 3.5 Examining sex distribution patterns in pituitary adenoma studies

**Fig. 5:**
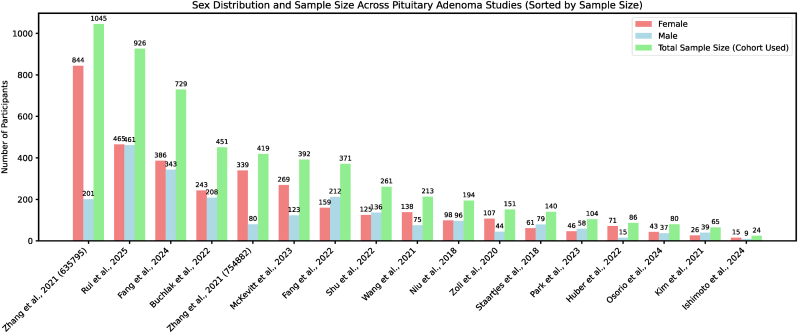
Bar chart illustrating the sex distribution and sample size across 17 pituitary adenoma studies, sorted by sample size in descending order.

Sex distribution patterns across 17 pituitary adenoma studies, as detailed in Fig. 5, encompass 5851 participants (3635 females, 2216 males), reflecting variations influenced by disease epidemiology and recruitment practices. Studies targeting Cushing's disease (CD) exhibit pronounced female predominance, with female counts ranging from 107 to 844 compared to male counts of 44 to 201, consistent with CD's higher prevalence in females (Zhang, et al. 2021a, 2021b; Zoli et al., 2020). In contrast, studies focusing on cavernous sinus invasion (CSI) detection show more balanced sex distributions, with female counts spanning 98 to 465 and male counts from 96 to 461, suggesting sex is not a significant predictor of invasiveness, supported by non-significant statistical findings (e.g., p = 0.073 in one study) (Fang, et al. 2022, 2024; Niu et al., 2019; Rui et al., 2025). Investigations emphasizing imaging advancements reveal variable sex ratios, with female counts ranging from 15 to 139 and male counts from 9 to 75, potentially reflecting differences in adenoma types or institutional recruitment biases (Ishimoto et al., 2024; Kim et al., 2021; Park et al., 2023). Subgroup analyses offer limited insights due to inconsistent reporting; for instance, one study notes a slight male predominance in a subgroup with higher Ki67 labeling index (64 males vs. 47 females), hinting at possible biological or selection factors affecting tumor aggressiveness (Shu et al., 2022). However, incomplete sex-specific data, such as in a study reporting only 261 participants' sex breakdown (125 females, 136 males) out of 362, restricts comprehensive analysis (Shu et al., 2022). To enhance the applicability of ML models and ensure equitable clinical translation, future studies must prioritize consistent and detailed sex-specific reporting across all participants and subgroups, accounting for sex as a potential confounder in outcomes like remission or invasiveness.

### 3.6 Assessing age demographics in pituitary adenoma research cohorts

**Fig. 6:**
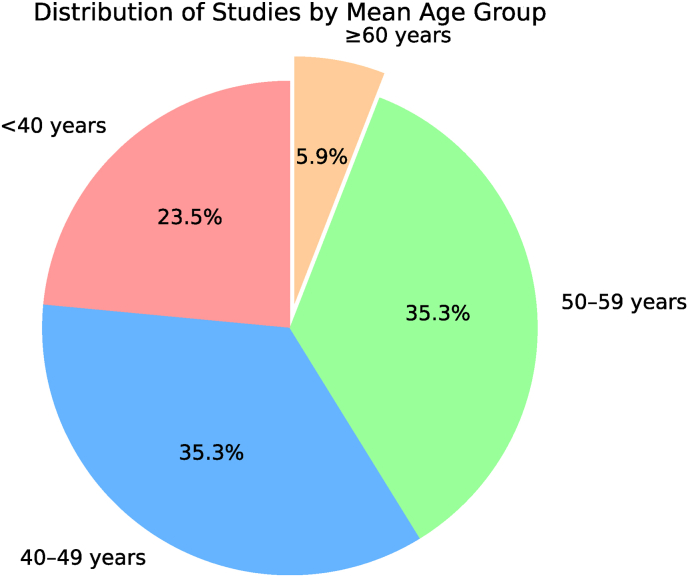
Pie chart illustrating the distribution of 17 pituitary adenoma studies by mean age group of participants. Categories are based on the mean (or median, where reported) age of each study: <40 years (23.5%, 4 studies), 40–49 years (35.3%, 6 studies), 50–59 years (35.3%, 6 studies), and ≥60 years (5.9%, 1 study), highlighting the variability in age demographics across the studies.

Age demographics across 17 pituitary adenoma studies, detailed in Table 3 and Fig. 6, show a weighted mean age of 45.07 years among 5851 participants, with a broad range (32–61 years) reflecting varied study objectives and patient populations. Cushing's disease (CD) studies typically include younger cohorts, such as those with median ages of 32 years (IQR 27–42) and 35 years (IQR 26–45) (Huber et al., 2022; Zhang et al., 2021b). In contrast, smaller or imaging-focused studies feature older participants, like one with a mean age of 61 years (n = 24) (Ishimoto et al., 2024). Most studies (70.6%) have mean or median ages between 40 and 59 years, categorized as <40 years (23.5%, 4 studies), 40–49 years (35.3%, 6 studies), 50–59 years (35.3%, 6 studies), and ≥60 years (5.9%, 1 study). Age dispersion varies, with standard deviations from 10 to 17.1 years, as seen in studies reporting 53.63 ± 16.86 years (n = 451) or 38.56 ± 11.95 years (n = 213) (Buchlak et al., 2022; Wang et al., 2021). Inconsistent reporting, such as using medians for non-normal distributions or omitting standard deviations, reduces precision in overall age variability estimates (SD 7.37 years). Standardized age reporting is essential for generalizable findings, particularly for age-sensitive outcomes like surgical remission or tumor invasiveness in machine learning-driven pituitary adenoma research.

**Table 3:** Mean Age of Participants Across Studies (Sorted by Mean Age) Summary of mean age (or median where reported) and SD of participants across 17 pituitary adenoma studies, sorted by mean age in ascending order. Notes indicate where medians are used due to non-normal distributions or specific cohort selections.

Study Reference	Mean Age ± SD (years)	Notes
Huber et al. (2022)	32 (IQR 27–42)	Median reported; non-normal distribution (Shapiro-Wilk test).
Zhang et al. (2021b)	35 (IQR 26–45)	Median reported; non-normal distribution (Wilcoxon test).
Zhang et al. (2021a)	37.86 ± 13.06	
Wang et al. (2021)	38.56 ± 11.95	Calculated from first group (n = 163): Women (108, 40.0 ± 12.3), Men (55, 35.87 ± 10.7).
Zoli et al. (2020)	41.1 ± 16.6	Range: 14–76 years.
Osorio et al. (2025)	43.84 ± 13.23	
McKevitt et al. (2023)	44.89 ± 15.15	
Niu et al. (2019)	47.02 ± 12.41	
Fang et al. (2024)	48.3 ± 14.1	
Shu et al. (2022)	49.82 ± 13.69	DL model cohort used (largest with mean age reported). Total sample size: 362.
Rui et al. (2025)	51 ± 13	Training cohort used (largest).
Buchlak et al. (2022)	53.63 ± 16.86	Preoperative cohort used (largest).
Kim et al. (2021)	54 ± 10	
Staartjes et al. (2018)	54.0 ± 17.1	
Fang et al. (2022)	55.52 ± 11.71	
Park et al. (2023)	59.4 ± 13.1	Range: 23–91 years.
Ishimoto et al. (2024)	61 ± 13

### 3.7 Profiling tumor characteristics in pituitary adenoma studies

#### 3.7.1 Adenoma size distribution

The diverse clinical presentation of pituitary adenomas is evident in the varying prevalence of microadenomas and macroadenomas across nine studies, as detailed in Table 4 (Huber et al., 2022; Ishimoto et al., 2024; McKevitt et al., 2023; Osorio et al., 2025; Park et al., 2023; Rui et al., 2025; Zhang, et al. 2021a, 2021b; Zoli et al., 2020). Investigations centered on CD highlight a high prevalence of microadenomas, with rates of 80.86% (845/1045) and 90.45% (379/419), consistent with the condition's typical smaller tumor sizes (Zhang, et al. 2021a, 2021b). Another study reports a more balanced distribution, with 53.0% microadenomas (80/151) and 23.8% MRI-undetectable adenomas (36/151), posing challenges for size classification (Zoli et al., 2020). In contrast, studies encompassing broader adenoma types or surgical cohorts show elevated macroadenoma rates, including 100% (104/104), 83.3% (20/24), 86% (68/79), and 100% (926/926), likely reflecting a focus on larger tumors requiring surgical intervention (Ishimoto et al., 2024; Osorio et al., 2025; Park et al., 2023; Rui et al., 2025). Other cohorts indicate 63.3% macroadenomas (248/392) or a near-even split with 47.7% macroadenomas (41/86) (Huber et al., 2022; McKevitt et al., 2023). Such variability underscores the influence of adenoma size on outcomes like invasiveness and surgical success, necessitating careful consideration in study design and interpretation.

**Table 4:** Tumor Characteristics Across Studies. Summary of tumor characteristics across 17 pituitary adenoma studies, including adenoma size (microadenomas vs. macroadenomas), functioning adenoma types, and CSI prevalence with confirmation methods. Studies without explicit data for a category are noted as “Not reported."

Study Reference	Adenoma Size (Micro/Macro)	Functioning Adenoma Types	CSI Prevalence	CSI Confirmation Method
Kim et al. (2021)	Not reported	22% (14/65): Prolactin (4), GH (7), ACTH (3); Non-functioning: 78% (51/65)	35% (23/65)	Clinical-radiologic consensus (Knosp criteria)
Fang et al. (2022)	Not reported	Not reported	27.5% (102/371)	Surgical evidence (intraoperative observation)
Huber et al. (2022)	Micro: 52.3% (45/86), Macro: 47.7% (41/86)	100% Prolactinomas (86/86)	22.4% (17/76)	Knosp grading ≥1 (MRI)
Park et al. (2023)	100% Macro (104/104)	12.5% (13/104): Prolactin (5), GH (4), ACTH (4); Non-functioning: 87.5% (91/104)	Not reported	Not reported
Wang et al. (2021)	Not reported	100% Functioning: First group (GH: 120/163, PRL: 23/163, ACTH: 14/163, TRH/TSH: 6/163); Second group (GH: 170/170)	First group: 76.7% (125/163), Second group: 78.2% (133/170)	Knosp grade ≥3 (MRI)
Zoli et al. (2020)	Micro: 53.0% (80/151), Macro: 23.2% (35/151), MRI-undetectable: 23.8% (36/151)	100% ACTH-secreting (151/151)	4.6% (7/151)	Intraoperative inspection
Zhang et al. (2021b)	Micro: 80.86% (845/1045), Macro: 19.14% (200/1045)	100% ACTH-secreting (1045/1045)	6.32% (66/1045)	IOMRI (Knosp 3 or 4)
Zhang et al. (2021a)	Micro: 90.45% (379/419), Macro: 9.55% (40/419)	100% ACTH-secreting (419/419)	6.21% (26/419)	IOMRI
Staartjes et al. (2018)	Not reported	Non-functioning: 68% (95/140), GH: 21% (29/140), Prolactin: 8% (11/140), ACTH: 2% (3/140), TSH: 1% (1/140), Plurihormonal: 1% (1/140)	Not reported	Not reported
Niu et al. (2019)	Not reported	Not reported	42.27% (82/194)	Intraoperative findings
McKevitt et al. (2023)	Macro: 63.3% (248/392), Micro: 36.7% (144/392)	100% Functioning: CA (143/392), SA (97/392), LA (96/392), MSA (30/392), Mixed GH/PRL (16/392), TA (10/392)	27.0% (106/392)	Preoperative MRI (Knosp >3)
Ishimoto et al. (2024)	Micro: 16.7% (4/24), Macro: 83.3% (20/24)	25% (6/24): Prolactin (3), ACTH (3); Non-functioning: 75% (18/24)	41.7% (10/24)	Surgical (7/10), MRI (2/10)
Shu et al. (2022)	Not reported	PA invasion: Non-functioning: 57.3% (141/246), Functioning: 42.7% (105/246; ACTH: 31, GH: 35, PRL: 26, TSH: 7, PP1: 6)	13.8% (34/246)	Intraoperative
Osorio et al. (2025)	Micro: 14% (11/79), Macro: 86% (68/79)	100% GH-secreting (80/80)	34% (27/80)	Preoperative MRI (radiologic impression)
Rui et al. (2025)	100% Macro (926/926)	Not reported	Training: 26.7% (218/816), Validation: 86.4% (95/110)	Intraoperative

#### 3.7.2 Functioning adenoma types

The hormonal diversity of pituitary adenomas is illuminated across 13 studies, showcasing a range of functioning adenoma profiles (Buchlak et al., 2022; Huber et al., 2022; Ishimoto et al., 2024; Kim et al., 2021; McKevitt et al., 2023; Osorio et al., 2025; Park et al., 2023; Shu et al., 2022; Staartjes et al., 2018; Wang et al., 2021; Zhang, et al. 2021a, 2021b; Zoli et al., 2020). Five studies exclusively focus on functioning adenomas, with three reporting 100% ACTH-secreting adenomas (151/151, 1045/1045, 419/419) due to their emphasis on CD (Zhang, et al. 2021a, 2021b; Zoli et al., 2020). Another study details a broad spectrum of functioning types: corticotrophic (36.5%, 143/392), somatotrophic (24.7%, 97/392), lactotrophic (24.5%, 96/392), mammosomatotrophic (7.7%, 30/392), mixed GH/PRL (4.1%, 16/392), and thyrotrophic (2.6%, 10/392), while a fifth reports 100% somatotrophic adenomas (80/80) (McKevitt et al., 2023; Osorio et al., 2025). One study highlights predominantly GH-secreting adenomas (73.6%, 120/163 in the first group; 100%, 170/170 in the second group), with smaller proportions of prolactin (14.1%), ACTH (8.6%), and TRH/TSH (3.7%) in the first group (Wang et al., 2021). Mixed cohorts show lower rates of functioning adenomas: 22% (14/65, prolactinomas: 4, GH: 7, ACTH: 3), 12.5% (13/104, prolactinomas: 5, GH: 4, ACTH: 4), 32% (45/140, GH: 29, prolactin: 11, ACTH: 3, TSH: 1, plurihormonal: 1), 25% (6/24, prolactin: 3, ACTH: 3), and 42.7% (105/246, ACTH: 31, GH: 35, prolactin: 26, TSH: 7, plurihormonal: 6) (Ishimoto et al., 2024; Kim et al., 2021; Park et al., 2023; Shu et al., 2022; Staartjes et al., 2018). Another study notes 22% secreting adenomas (99/451, acromegaly: 41, gonadotroph: 18), though a complete breakdown is unavailable (Buchlak et al., 2022). These differences in functioning vs. non-functioning adenoma prevalence may shape tumor behavior and clinical outcomes, warranting attention in future research.

#### 3.7.3 Cavernous sinus invasion prevalence

Significant variation in CSI prevalence, ranging from 4.6% to 86.4%, is observed across 14 studies, as outlined in Table 4 (Fang, et al. 2022, 2024; Huber et al., 2022; Ishimoto et al., 2024; Kim et al., 2021; McKevitt et al., 2023; Niu et al., 2019; Osorio et al., 2025; Rui et al., 2025; Shu et al., 2022; Staartjes et al., 2018; Wang et al., 2021; Zhang, et al. 2021a, 2021b; Zoli et al., 2020). Studies targeting CD report lower CSI rates, such as 4.6% (7/151, intraoperative), 6.32% (66/1045, IOMRI), and 6.21% (26/419, IOMRI), likely due to the predominance of smaller tumors in this condition (Zhang, et al. 2021a, 2021b; Zoli et al., 2020). Conversely, studies with broader adenoma types exhibit higher CSI rates: 76.7% (125/163) and 78.2% (133/170) in two cohorts (Knosp ≥3, MRI), and 26.7% (218/816) in a training cohort but 86.4% (95/110) in a validation cohort (intraoperative) (Rui et al., 2025; Wang et al., 2021). Moderate CSI rates are noted in other studies: 35% (23/65, radiologic consensus), 27.5% (102/371, surgical), 42.27% (82/194, surgical), 27.0% (106/392, Knosp >3), 25.5% (186/729, surgical), 41.7% (10/24, surgical/MRI), 13.8% (34/246, surgical), and 34% (27/80, radiologic) (Fang, et al. 2022, 2024; Ishimoto et al., 2024; Kim et al., 2021; McKevitt et al., 2023; Niu et al., 2019; Osorio et al., 2025; Shu et al., 2022). One study reports a CSI rate of 22.4% (17/76, Knosp ≥1), though its lower Knosp threshold may inflate invasion estimates compared to studies using Knosp ≥3 (Huber et al., 2022). The diversity in CSI confirmation methods (surgical vs. MRI-based) and criteria (e.g., Knosp thresholds) highlights the importance of standardized approaches to enhance comparability across studies.

### 3.8 Gender and adenoma size patterns in pituitary adenoma studies

To address the need for subgroup analysis in pituitary adenoma research, the distribution of microadenomas (<10 mm) and macroadenomas (≥10 mm) by gender was examined across the 17 included studies, utilizing ML and DL methodologies for enhanced diagnostic insights. Sex-specific patterns in adenoma size and their potential impact on clinical outcomes, such as CSI and postoperative remission, were explored to inform preoperative planning and prognostication.

Data on adenoma size distribution were available from nine studies, with varying levels of detail on gender-specific breakdowns (Huber et al., 2022; Ishimoto et al., 2024; McKevitt et al., 2023; Osorio et al., 2025; Park et al., 2023; Rui et al., 2025; Zhang, et al. 2021a, 2021b; Zoli et al., 2020). Table 5 summarizes the distribution of microadenomas and macroadenomas by gender, with percentages calculated from reported patient counts and sex proportions. Where exact gender-specific adenoma size data were unavailable, estimations were derived based on overall sex ratios.

**Table 5:** Distribution of Microadenomas and Macroadenomas by Gender Across Studies. Study (Zoli et al., 2020)provided explicit gender-specific adenoma size data, while studies (Fang, et al. 2022, 2024; Kim et al., 2021; Niu et al., 2019; Shu et al., 2022; Staartjes et al., 2018) lacked sufficient adenoma size information and were excluded. Study (Zoli et al., 2020) also reported 23.8% MRI-undetectable adenomas, not included in microadenoma/macroadenoma calculations.

Study	Total Patients	Female (%)	Male (%)	Microadenoma (%)	Macroadenoma (%)	Microadenoma (Female)	Microadenoma (Male)	Macroadenoma (Female)	Macroadenoma (Male)
Huber et al. (2022)	86	71 (82.6%)	15 (17.4%)	45 (52.3%)	41 (47.7%)	37 (82.2%)	8 (17.8%)	34 (82.9%)	7 (17.1%)
Park et al. (2023)	104	46 (43.8%)	58 (56.2%)	0 (0%)	104 (100%)	0 (0%)	0 (0%)	46 (43.8%)	58 (56.2%)
Zoli et al. (2020)	151	107 (70.8%)	44 (29.2%)	80 (53.0%)	35 (23.2%)	60 (75.0%)	20 (25.0%)	22 (62.9%)	13 (37.1%)
Zhang et al. (2021b)	1045	844 (80.8%)	201 (19.2%)	845 (80.9%)	200 (19.1%)	683 (80.8%)	162 (19.2%)	161 (80.5%)	39 (19.5%)
Zhang et al. (2021a)	419	339 (80.9%)	80 (19.1%)	379 (90.5%)	40 (9.5%)	307 (81.0%)	72 (19.0%)	32 (80.0%)	8 (20.0%)
McKevitt et al. (2023)	392	269 (68.6%)	123 (31.4%)	144 (36.7%)	248 (63.3%)	99 (68.8%)	45 (31.2%)	170 (68.5%)	78 (31.5%)
Ishimoto et al. (2024)	24	15 (62.5%)	9 (37.5%)	2 (8.3%)	22 (91.7%)	1 (50.0%)	1 (50.0%)	14 (63.6%)	8 (36.4%)
Osorio et al. (2025)	80	43 (54.0%)	37 (46.0%)	11 (14.0%)	68 (86.0%)	6 (54.5%)	5 (45.5%)	37 (54.4%)	31 (45.6%)
Rui et al. (2025)	729	386 (53.0%)	343 (47.0%)	0 (0%)	729 (100%)	0 (0%)	0 (0%)	386 (53.0%)	343 (47.0%)

**Fig. 7:**
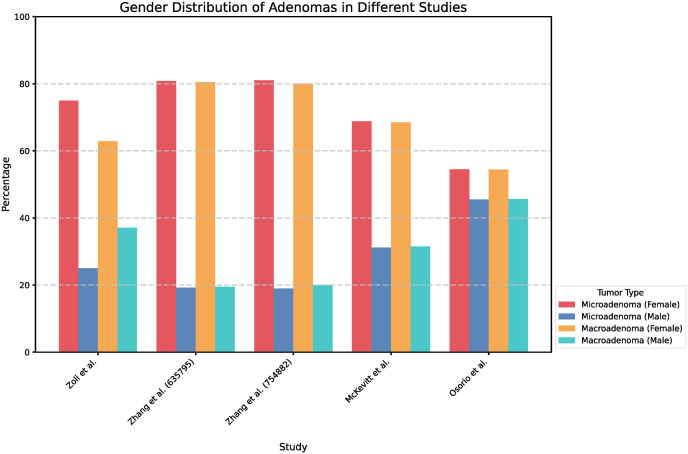
Gender-Specific distribution of Adenoma sizes across studies.

Distinct sex-specific patterns in adenoma size distribution were observed. A high prevalence of microadenomas (80.9–90.5%) and marked female predominance (80.8–80.9%) were noted in studies focusing on Cushing's disease, consistent with the epidemiology of adrenocorticotropic hormone (ACTH)-secreting adenomas (Zhang, et al. 2021a, 2021b). In contrast, a predominance of macroadenomas (86–100%) with more balanced gender distributions (43.8–53.0% female) was found in studies with broader adenoma types or surgical cohorts (Park et al., 2023; Rui et al., 2025). Explicit gender-specific data from study (Zoli et al., 2020) indicated a higher proportion of microadenomas in females (75% vs. 25% in males) compared to macroadenomas (62.9% vs. 37.1%). A chi-square test conducted on these data revealed no significant difference in adenoma size distribution between genders (p = 0.4103), suggesting that sex may not be a primary determinant of adenoma size in this cohort (Zoli et al., 2020). These gender-specific distributions are illustrated in Fig. 7, highlighting the predominance of microadenomas in female-dominated cohorts and macroadenomas in balanced cohorts.

The influence of gender and adenoma size on clinical outcomes was also assessed. Higher early remission rates (95% vs. 82.6% for macroadenomas) and lower CSI rates (4.6% overall, with Knosp grade ≥3 in 0.4%) were associated with microadenomas in study (Zoli et al., 2020). Similarly, lower CSI rates (6.2–6.3%) were reported in microadenoma-dominant cohorts, potentially due to reduced invasive potential of smaller tumors (Zhang, et al. 2021a, 2021b). Conversely, a higher CSI rate (25.5%) was observed in study (Rui et al., 2025), with exclusively macroadenomas, where females showed a slightly higher prevalence (57.3% vs. 47.2%, p = 0.010) in the training cohort (Rui et al., 2025). Higher remission rates for microadenomas (86.1% vs. 55.2% for macroadenomas) were noted in study (McKevitt et al., 2023), with females achieving higher remission across functional adenoma subtypes (e.g., 80.7% for corticotrophic adenomas vs. 62.5% in males, p = 0.030) (McKevitt et al., 2023).

### 3.9 Evaluating pathological confirmation in pituitary adenoma studies

Accurate diagnosis of pituitary adenomas relies heavily on pathological validation, with 12 studies reporting confirmation rates spanning from 4% to 100%, as outlined in Table 6 (Fang et al., 2022; Huber et al., 2022; Ishimoto et al., 2024; Kim et al., 2021; McKevitt et al., 2023; Osorio et al., 2025; Park et al., 2023; Rui et al., 2025; Shu et al., 2022; Zhang, et al. 2021a, 2021b; Zoli et al., 2020). Seven studies achieved complete validation (100%) through surgical resection and immunohistochemical staining, covering cohorts of 371/371, 86/86, 1045/1045, 392/392, 246/246, 80/80, and 926/926 participants (Fang et al., 2022; Huber et al., 2022; McKevitt et al., 2023; Osorio et al., 2025; Rui et al., 2025; Shu et al., 2022; Zhang et al., 2021b). Another study reported a high confirmation rate of 93.56% (392/419) via histological analysis, with the remaining cases limited by insufficient specimens (Zhang et al., 2021a). A confirmation rate of 82.4% (124/151) was observed in one study, with 17.6% lacking adequate samples, potentially introducing diagnostic uncertainty (Zoli et al., 2020). In contrast, two studies reported notably lower confirmation rates of 4% (2/45 residual tumor cases) and 4.7% (3/64), relying predominantly on clinical-radiologic consensus for unconfirmed cases, which may compromise diagnostic precision (Kim et al., 2021; Park et al., 2023). One study validated 75% of cases (18/24) pathologically, with the remaining 25% (6/24) based on clinical-radiologic consensus, reflecting potential inconsistencies in diagnostic approaches (Ishimoto et al., 2024). These variations in confirmation rates and methods, ranging from surgical resection to clinical-radiologic consensus, signal a critical need for uniform pathological protocols to strengthen the foundation of diagnostic accuracy in pituitary adenoma research.

**Table 6:** Pathological Confirmation of Adenomas Across Studies. Summary of pathological confirmation rates and methods across 12 pituitary adenoma studies. Notes highlight limitations or additional details, such as reliance on clinical-radiologic consensus or exclusion criteria.

Study Reference	Pathological Confirmation Rate	Method of Confirmation	Notes
Kim et al. (2021)	4% (2/45, residual tumor cases)	Surgical resection (second surgery)	Majority via clinical-radiologic consensus
Fang et al. (2022)	100% (371/371)	Surgical resection	CSI not pathologically confirmed
Huber et al. (2022)	100% (86/86)	Immunohistochemical staining	Confirmed as prolactinomas
Park et al. (2023)	4.7% (3/64)	Surgical resection (second surgery)	Majority via clinical-radiologic consensus
Zoli et al. (2020)	82.4% (124/151)	Surgical resection, immunohistochemistry	17.6% with insufficient specimens
Zhang et al. (2021b)	100% (1045/1045)	Surgical resection	Exclusion of 150 pathology-negative cases
Zhang et al. (2021a)	93.56% (392/419)	Surgical resection, immunohistochemistry	Remainder lacked sufficient specimens
McKevitt et al. (2023)	100% (392/392)	Surgical resection (per 2022 WHO classification)	-
Ishimoto et al. (2024)	75% (18/24)	Surgical resection	25% via clinical-radiologic consensus
Shu et al. (2022)	100% (246/246)	Surgical resection, immunohistochemistry	Ki67LI assessed within 7 days post-ETTS
Osorio et al. (2025)	100% (80/80)	Surgical resection, pathological staining	-
Rui et al. (2025)	100% (926/926)	Surgical resection	-

### 3.10 Exploring Knosp Grade patterns in pituitary adenoma invasiveness

The classification of pituitary adenoma invasiveness through Knosp grading, as reported in seven studies and detailed in Table 7, reveals diverse patterns of CSI (Fang, et al. 2022, 2024; McKevitt et al., 2023; Niu et al., 2019; Rui et al., 2025; Staartjes et al., 2018; Wang et al., 2021; Zoli et al., 2020). Higher Knosp grades consistently correlate with greater invasion, with Grade 4 exhibiting up to 100% CSI in some cohorts, while Grades 0–1 show minimal invasion (0–2%), reflecting a robust predictive trend (Fang, et al. 2022, 2024; Rui et al., 2025). Intermediate grades, such as 3A, display variable CSI rates (e.g., 35.5–44%), indicating potential diagnostic ambiguity and inconsistencies in grading criteria across studies (Fang et al., 2024; Rui et al., 2025). Specific focus on grades like 2 and 3 reveals marked CSI differences—14.6% for Grade 2 versus 65.7% for Grade 3—pointing to a critical threshold for invasiveness (Niu et al., 2019). Studies grouping grades (e.g., 0–2 vs. >3) obscure finer distinctions, limiting precision in evaluating invasion risk (McKevitt et al., 2023). Variations in confirmation methods, ranging from MRI-based grading to intraoperative findings, further challenge comparability, with some studies achieving high accuracy (e.g., 87.68% for Grades 0–2) but reduced sensitivity (e.g., 63.64%) for detecting invasion (Rui et al., 2025). These disparities call for unified Knosp grading standards to refine predictions of tumor behavior and enhance clinical decision-making.

**Table 7:** Knosp Grade Distribution Across Studies. summary of Knosp grade distributions and CSI rates across seven pituitary adenoma studies. The Knosp Grade Distribution column is split into subcolumns for each grade (Grade 0, Grade 1, Grade 2, Grade 3A, Grade 3B, Grade 4, and grouped categories), and the CSI Rates per Grade column is similarly split into subcolumns for each grade. Includes additional metrics (e.g., accuracy, sensitivity, specificity) and notes.

Study Reference	Knosp Grade Distribution							CSI Rates per Grade							Additional Metrics	Notes
	Grade 0	Grade 1	Grade 2	Grade 3A	Grade 3B	Grade 4	Grouped Grades	Grade 0	Grade 1	Grade 2	Grade 3A	Grade 3B	Grade 4	Grouped Grades		
Fang et al. (2022)	5.4% (20/371)	28.8% (107/371)	22.1% (82/371)	28.0% (104/371)	5.9% (22/371)	9.7% (36/371)	-	0% (0/20)	3.7% (4/107)	18.3% (15/82)	37.5% (39/104)	54.5% (12/22)	88.9% (32/36)	-	Significant difference in CSI rates (p < 0.001, chi-square test)	Intermediate grades (2–3A) show weak reliability (18.3%–37.5% CSI)
Wang et al. (2021)	First Group: 1.8% (3/163), Second Group: 1.8% (3/170)	First Group: 8.0% (13/163), Second Group: 7.1% (12/170)	First Group: 13.5% (22/163), Second Group: 12.9% (22/170)	First Group: 22.1% (36/163), Second Group: 21.8% (37/170)	First Group: 20.9% (34/163), Second Group: 22.9% (39/170)	First Group: 33.7% (55/163), Second Group: 33.5% (57/170)	-	Not reported	Not reported	Not reported	Not reported	Not reported	Not reported	First Group: Grades ≥3 (76.7%, 125/163); Second Group: Grades ≥3 (78.2%, 133/170)	P = 0.678 (first group), P = 0.827 (second group); Confusion Matrix (n = 32): Accuracy 81.25% (26/32); Grade 3B ∼50% correct	No per-grade CSI rates; high CSI reflects invasive cohort
Zoli et al. (2020)	Not reported	Not reported	Not reported	Not reported	Not reported	Not reported	Grades ≥3: 0.4% (6/151)	Not reported	Not reported	Not reported	Not reported	Not reported	Not reported	Implied high for Grades ≥3	-	Limited data on full distribution restricts granularity
Staartjes et al. (2018)	18% (25/140)	21% (29/140)	32% (45/140)	17% (24/140)	6% (9/140)	6% (8/140)	-	Not reported	Not reported	Not reported	Not reported	Not reported	Not reported	Grades 3A, 3B, 4: 29% (41/140)	-	No per-grade CSI rates reported
Niu et al. (2019)	Not reported	Not reported	45.88% (89/194); Training: 38.14% (37/97), Test: 53.61% (52/97)	Not reported	Not reported	Not reported	Grade 3: 54.12% (105/194); Training: 61.86% (60/97), Test: 46.39% (45/97)	Not reported	Not reported	14.6% (13/89); Training: 18.9% (7/37), Test: 11.5% (6/52)	Not reported	Not reported	Not reported	Grade 3: 65.7% (69/105); Training: 66.7% (40/60), Test: 64.4% (29/45)	-	Grade 2 variability (11.5–18.9%) highlights diagnostic challenge
McKevitt et al. (2023)	Not reported	Not reported	Not reported	Not reported	Not reported	Not reported	Low (0–2): 73% (286/392), High (>3): 27% (106/392)	Not reported	Not reported	Not reported	Not reported	Not reported	Not reported	Assumed 100% for high grades per definition, not quantified	Low Knosp grade (0–2) predicts remission (OR 1.74, 95% CI 1.015–2.996)	No detailed breakdown for Grades 0, 1, 2, 3A, 3B, 4
Fang et al. (2024)	7.4% (54/729)	27.7% (202/729)	26.5% (193/729)	26.5% (193/729)	5.9% (43/729)	6.0% (44/729)	0–1: 35.1% (256/729), 2–4: 64.9% (473/729), 0–2: 61.6% (449/729), 3A–4: 38.4% (280/729), 0–3A: 88.1% (642/729), 3B–4: 11.9% (87/729)	0% (0/54)	2.5% (5/202)	12.4% (24/193)	44.0% (85/193)	67.4% (29/43)	97.7% (43/44)	0–1: 2.0%, 2–4: 38.3%, 0–2: 6.5%, 3A–4: 56.1%, 0–3A: 17.8%, 3B–4: 82.8%	-	Grade 3A's moderate CSI rate (44.0%) indicates diagnostic ambiguity
Rui et al. (2025)	Right CS: 22.8% (155/680 non-CSI), Left CS: 26.5% (186/701 non-CSI)	Right CS: 36.9% (251/680 non-CSI, 2/136 CSI), Left CS: 35% (245/701 non-CSI, 4/115 CSI)	Right CS: 28.8% (196/680 non-CSI, 27/136 CSI), Left CS: 30.2% (212/701 non-CSI, 32/115 CSI)	Right CS: 11.5% (78/680 non-CSI, 43/136 CSI), Left CS: 8.3% (58/701 non-CSI, 42/115 CSI)	Right CS: 0% (0/680 non-CSI, 10/136 CSI), Left CS: 0% (0/701 non-CSI, 5/115 CSI)	Right CS: 0% (0/680 non-CSI, 54/136 CSI), Left CS: 0% (0/701 non-CSI, 32/115 CSI)	-	Right CS: 0% (0/155), Left CS: 0% (0/186)	Right CS: 0.8% (2/253), Left CS: 1.6% (4/249)	Right CS: 12.1% (27/223), Left CS: 13.1% (32/244)	Right CS: 35.5% (43/121), Left CS: 42% (42/100)	Right CS: 100% (10/10), Left CS: 100% (5/5)	Right CS: 100% (54/54), Left CS: 100% (32/32)	-	Knosp Accuracy: 0–2 (87.68%, CI: 85.39–89.97), 3 (57.62%, CI: 52.14–63.10), 4 (100%), All (87.68%); MTMAU-Net Accuracy: 0–2 (87.20%), 3 (83.33%), 4 (100%), All (88.05%); Sensitivity: 0–2 (63.64%), 3 (70.00%), 4 (100%), All (71.43%); Specificity: 0–2 (92.23%), 3 (92.86%), 4 (100%), All (94.02%)	No validation cohort Knosp data reported; Grade 3A variability (35.5% right, 42% left) noted

### 3.11 Characterizing imaging approaches in pituitary adenoma assessment

**Fig. 8:**
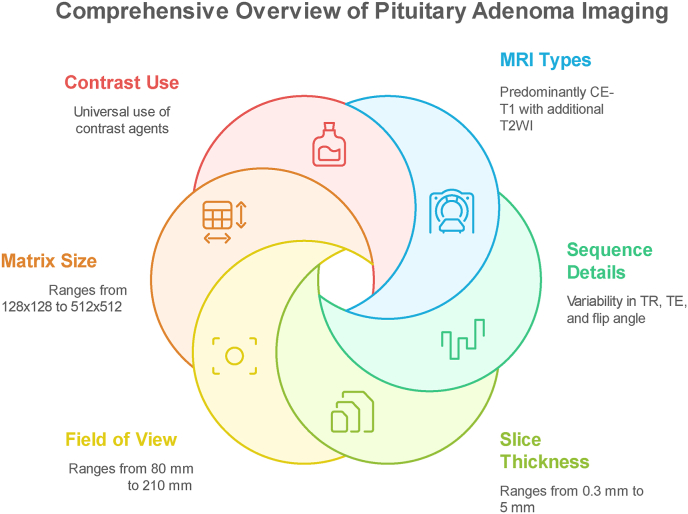
Visual summary of imaging modalities across pituitary adenoma studies, illustrating imaging types (predominantly CE-T1, with T2WI in some), MRI sequence details (e.g., TR/TE ranges), slice thicknesses (0.3 mm to 5 mm), field of view (80 mm to 210 mm), matrix sizes (128x128 to 512x512), and contrast use (100% gadolinium-based) (Fang et al., 2022; Ishimoto et al., 2024; Kim et al., 2021; Niu et al., 2019; Park et al., 2023; Rui et al., 2025; Shu et al., 2022; Wang et al., 2021).

The evaluation of pituitary adenomas through diverse imaging approaches, as reported in 15 studies, encompasses a range of MRI types, sequence specifications, slice thicknesses, field of view (FOV) dimensions, matrix sizes, and contrast use (Fang, et al. 2022, 2024; Ishimoto et al., 2024; Kim et al., 2021; McKevitt et al., 2023; Niu et al., 2019; Osorio et al., 2025; Park et al., 2023; Rui et al., 2025; Shu et al., 2022; Staartjes et al., 2018; Wang et al., 2021; Zhang, et al. 2021a, 2021b; Zoli et al., 2020). CE-T1 MRI, enhanced with gadolinium-based agents, serves as the cornerstone across all studies, aligning with established pituitary imaging protocols. Five studies complement CE-T1 with T2WI to enhance feature extraction, though T2WI plays a secondary role in assessing CSI (Niu et al., 2019; Osorio et al., 2025; Rui et al., 2025; Shu et al., 2022; Wang et al., 2021). Three studies incorporate advanced 3D acquisitions, such as CUBE and spoiled gradient recalled (SPGR), alongside standard 2D CE-T1, to achieve higher resolution for sellar region imaging (Ishimoto et al., 2024). Fig. 8 visually summarizes these imaging approaches, detailing variations in sequence types, slice thicknesses (0.3 mm to 5 mm), FOVs (80 mm to 210 mm), matrix sizes (128x128 to 512x512), and consistent contrast use (Fang et al., 2022; Ishimoto et al., 2024; Kim et al., 2021; Niu et al., 2019; Park et al., 2023; Rui et al., 2025; Shu et al., 2022; Wang et al., 2021). Thinner slices (e.g., 0.3 mm CUBE, 1 mm CE-T1) enhance resolution but may extend acquisition times, while thicker slices (e.g., 5 mm) risk reduced detail (Ishimoto et al., 2024; Niu et al., 2019; Park et al., 2023; Rui et al., 2025; Shu et al., 2022). Reported FOVs range from smaller values (e.g., 80 mm for CUBE, focusing on the sellar region) to larger ones (e.g., 210 mm, capturing broader anatomy) (Ishimoto et al., 2024; Kim et al., 2021; Rui et al., 2025; Shu et al., 2022). Matrix sizes vary from 128x128 to 512x512, with higher matrices (e.g., 512x512) improving resolution but demanding greater processing power, which influences segmentation accuracy (Ishimoto et al., 2024; Kim et al., 2021; Niu et al., 2019; Rui et al., 2025; Shu et al., 2022; Wang et al., 2021). Sequence parameters, such as repetition time (TR), echo time (TE), and flip angle, differ significantly (e.g., TR from 6.6 ms for SPGR to 5400 ms for T2WI), impacting image contrast and SNR (Ishimoto et al., 2024; Niu et al., 2019; Rui et al., 2025; Shu et al., 2022; Wang et al., 2021). While universal contrast use enhances tumor visualization, the lack of uniformity in sequence parameters, slice thickness, and FOV calls for harmonized imaging protocols to ensure consistent and reliable outcomes across studies. Table 8 summarizes these imaging characteristics comprehensively.

**Table 8:** Imaging Modalities Across Studies. Comprehensive summary of imaging modalities across 15 pituitary adenoma studies, detailing imaging type, MRI sequence details, slice thickness, field of view, matrix size, and contrast use. All reported data from Initial Data, Supplementary Data, and Analysis columns are included.

Study Reference	Imaging Type	MRI Sequence Details	Slice Thickness (Motamed et al.)	Field of View (Motamed et al.)	Matrix Size	Contrast Use
Kim et al. (2021)	CE-T1 (coronal)	2D FSPGR (phantom): TR/TE 12.6/6.0 ms, FOV 210 × 210 mm, matrix 512x512	1 mm and 3 mm (clinical); 3 mm (phantom)	210x210 (phantom); clinical not reported	512x512 (phantom); clinical likely 256x256	100% gadolinium (type not specified)
Fang et al. (2022)	CE-T1 (coronal)	Not reported	Not reported	Not reported	256x256 (post-preprocessing)	100% contrast (likely gadolinium)
Park et al. (2023)	Sagittal T1-w, Coronal T2-w, Coronal T1-w, Sagittal/Axial CE-T1-w, Coronal CE-T1-w	3-mm routine: TR/TE 500/13 ms, flip angle 90°; 1-mm DLR: TR/TE 698/16 ms, flip angle 90°	3 mm (routine); 1 mm (DLR)	180x180	260x260 (3-mm); 320x260 (1-mm DLR)	100% gadolinium (0.1 mmol/kg)
Wang et al. (2021)	CE-T1, T1-w, T2-w	CE-T1: TR/TE 400/9.2 ms, 512x512x8 pixels	3 mm, spacing 0.39 mm	Not reported (inferred 200–250 mm)	512x512x8	100% gadolinium (type not specified)
Zoli et al. (2020)	1.5-T/3-T MRI with gadolinium	Not reported	Not reported	Not reported	Not reported	100% gadolinium (type not specified)
Zhang et al. (2021b)	T1-w, T2-w, CE-T1, dynamic CE-T1	Not reported	Not reported	Not reported	Not reported	100% gadolinium (type not specified)
Zhang et al. (2021a)	T1-w, T2-w, CE-T1, dynamic CE-T1	Not reported	Not reported	Not reported	Not reported	100% gadolinium (type not specified)
Staartjes et al. (2018)	3-T volumetric CE-T1	Not reported	Not reported	Not reported	Not reported	100% contrast (likely gadolinium)
Niu et al. (2019)	CE-T1, T2-w	CE-T1 coronal/sagittal: TR/TE 1200/11 ms; CE-T1 axial: TR/TE 2000/9.8 ms; T2: TR/TE 4500/84 ms	CE-T1: 3 mm (coronal/sagittal), 5 mm (axial); T2: 5 mm	Not reported (inferred 200–250 mm)	CE-T1: 256x256 (coronal/sagittal), 220x185 (axial); T2: 259x384	100% gadolinium (type not specified)
McKevitt et al. (2023)	Preoperative MRI with/without gadolinium	Not reported	Not reported	Not reported	Not reported	100% gadolinium (type not specified)
Fang et al. (2024)	CE-T1 (coronal)	Not reported	Not reported	Not reported	Not reported	100% contrast (likely gadolinium)
Ishimoto et al. (2024)	CE-T1 (CUBE, 1-mm 2D T1WI, SPGR)	CUBE: TR/TE 500/16 ms, flip angle VRFA; 1-mm T1WI: TR/TE 580/11 ms, flip angle 73°; SPGR: TR/TE 6.6/2.1 ms, flip angle 12°	CUBE: 0.3 mm; 1-mm T1WI: 1 mm; SPGR: 1 mm	CUBE: 80 mm; 1-mm T1WI: 160 mm; SPGR: 160 mm	CUBE: 128x128; 1-mm T1WI: 224x224; SPGR: 224x224	100% gadolinium (type not specified)
Shu, et al. (2022)	CE-T1, T2-w	ceT1WI: TR/TE 1650/3.02 ms, flip angle 15°; T2WI: TR/TE 5400/98 ms, flip angle 150°	ceT1WI: 1 mm; T2WI: 3 mm	ceT1WI: 130 mm; T2WI: 90.625 mm	ceT1WI: 192x256x176; T2WI: 290x290x30	100% Gadobutrol (Bayer AG)
Osorio et al. (2025)	CE-T1 (implied)	Not reported	Not reported	Not reported	Not reported	100% contrast (likely gadolinium)
Rui et al. (2025)	CE-T1 (coronal)	GE: TR/TE 400/13 ms; Siemens: TR/TE 401/8.6 ms, flip angle 130°	2 mm	GE: 200 mm; Siemens: 210 mm	GE: 288x192; Siemens: 130x130	100% gadopentetate dimeglumine (0.1 mmol/kg)

### 3.12 Machine learning and deep learning applications analysis

#### 3.12.1 Advancing pituitary adenoma research with diverse AI models and applications

**Fig. 9:**
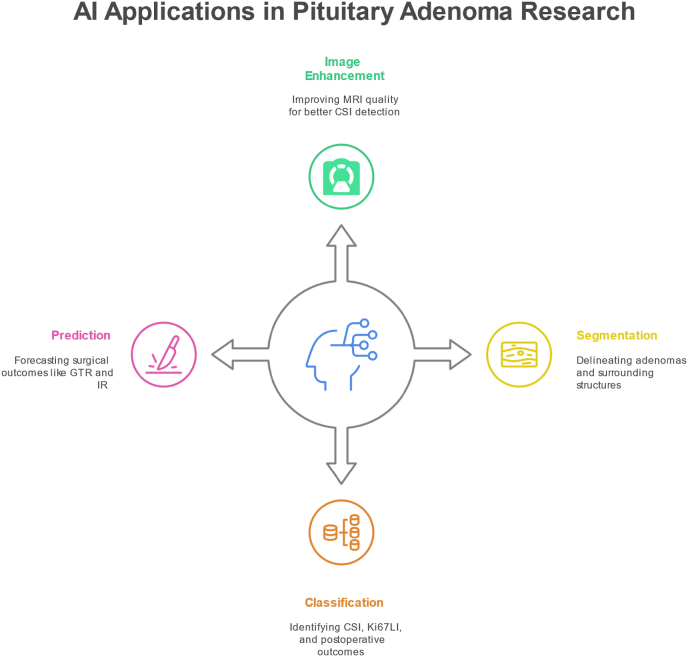
Overview of AI applications in pituitary adenoma research.

The application of AI, through ML and DL methodologies, has reshaped pituitary adenoma research by addressing critical clinical needs through diverse computational strategies. Image enhancement techniques, such as DLR frameworks like AIR Recon DL, a CNN-based model developed by GE Healthcare, improve MRI quality by boosting SNR and minimizing artifacts, aiding in CSI detection (Ishimoto et al., 2024; Kim et al., 2021; Park et al., 2023). Segmentation tasks leverage advanced architectures, including GSU-Net (a U-Net variant with shape stream integration) and MTMAU-Net, to delineate pituitary adenomas (PAs) and surrounding structures preoperatively, while simultaneously predicting outcomes like CSI(Rui et al., 2025; Wang et al., 2021). Classification efforts target a range of objectives, from preoperative CSI identification and Ki67 labeling index (Ki67LI) determination to postoperative outcomes like remission or QoL improvement, utilizing models such as Resnet50, support vector machines (SVM), and random forests (RF) (Buchlak et al., 2022; Fang, et al. 2022, 2024; McKevitt et al., 2023; Niu et al., 2019; Osorio et al., 2025; Shu et al., 2022; Zoli et al., 2020). Prediction tasks focus on forecasting surgical outcomes, such as GTR and immediate remission (IR), employing logistic regression (LR), RF, and ensemble methods like stacking, which integrate multiple weak learners for enhanced performance (Staartjes et al., 2018; Zhang, et al. 2021a, 2021b). Fig. 9 visually categorizes these AI applications into image enhancement, segmentation, classification, and prediction, illustrating the diversity of model architectures, from single models like Resnet50 and U-Net to ensembles like AdaBoost and stacking, demonstrating AI's versatility in tackling complex clinical challenges and paving the way for refined model selection to optimize diagnostic and therapeutic precision.

#### 3.12.2 Navigating data preprocessing and feature extraction in pituitary adenoma research

**Fig. 10:**
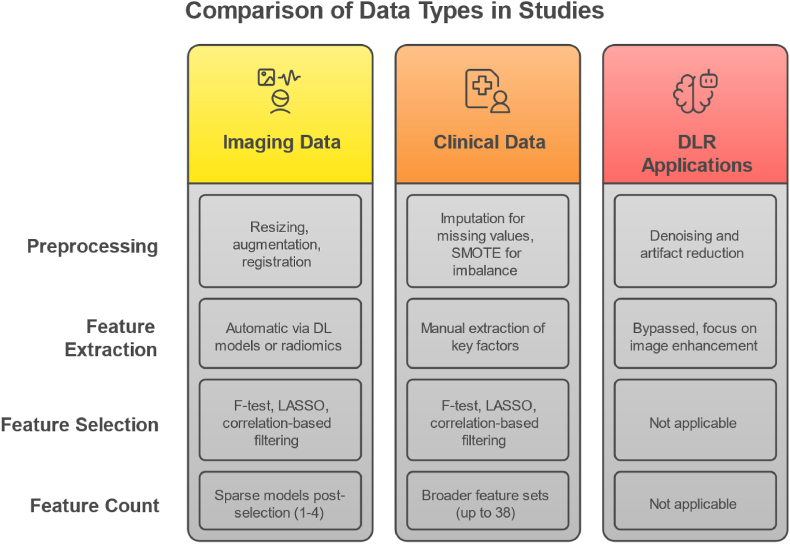
Comparison of data types in pituitary adenoma studies, detailing preprocessing.

The preparation and analysis of data in pituitary adenoma research vary significantly based on input types, imaging, clinical, or DLR-focused, affecting the performance, robustness, and generalizability of AI models. For imaging data, preprocessing involves resizing MRI scans to uniform dimensions, such as 256x256 or 428x428 pixels for CNN compatibility, coupled with augmentation techniques like horizontal flipping and 90° rotation to enhance dataset diversity and reduce overfitting (Fang et al., 2022; Niu et al., 2019; Shu et al., 2022). Some studies employ registration via tools like 3D Slicer to align coronal images, ensuring spatial consistency critical for accurate PA segmentation (Shu et al., 2022). Conversely, DLR applications integrate preprocessing within proprietary pipelines, emphasizing denoising to boost SNR and minimize Gibbs ringing artifacts, often bypassing traditional steps like normalization or augmentation due to their focus on image enhancement (Ishimoto et al., 2024; Park et al., 2023). Clinical data studies address imperfections through imputation methods, such as K-nearest neighbors (KNN) for 1.7–3.9% missing data in remission prediction or median/mode replacement for numerical/categorical variables, while tackling class imbalance (e.g., remission vs. non-remission) with Synthetic Minority Oversampling Technique (SMOTE) to generate synthetic samples (Buchlak et al., 2022; Huber et al., 2022; McKevitt et al., 2023; Osorio et al., 2025; Zhang, et al. 2021a, 2021b; Zoli et al., 2020). Feature extraction approaches differ markedly: imaging studies leverage end-to-end DL models like Resnet50 for automatic hierarchical feature learning from MRI scans or radiomics pipelines extracting high-dimensional features (e.g., 2553 features from CE-T1/T2WI, including intensity, shape, texture, and wavelet features) to capture intricate PA characteristics (Fang et al., 2022; Niu et al., 2019; Rui et al., 2025; Shu et al., 2022). Clinical studies prioritize manually extracted interpretable features, such as age, tumor size (e.g., diameter in mm), Knosp grade, and preoperative ACTH levels, emphasizing clinical relevance (McKevitt et al., 2023; Osorio et al., 2025; Staartjes et al., 2018; Wang et al., 2021; Zhang, et al. 2021a, 2021b; Zoli et al., 2020). DLR studies forgo feature extraction, focusing solely on image quality enhancement, limiting their role in predictive tasks like classification (Ishimoto et al., 2024; Kim et al., 2021; Park et al., 2023). Feature selection, when applied, employs techniques like F-test for univariate analysis (selecting top features by p-values), least absolute shrinkage and selection operator (LASSO) with 5-fold cross-validation to eliminate less informative features, or correlation-based filtering to remove redundant features (e.g., correlation coefficient >0.75), yielding sparse models with 3–4 features in imaging studies (e.g., Sphericity, Minimum_HL, ICA wrapped degree) or broader sets of up to 38 features in clinical models (e.g., including age, sex, tumor size, histological findings) (McKevitt et al., 2023; Niu et al., 2019; Rui et al., 2025; Wang et al., 2021; Zhang, et al. 2021a, 2021b). Fig. 10 visually contrasts these strategies across data types, detailing preprocessing, feature extraction, selection, and final feature counts, revealing their influence on model design. With final feature counts ranging from 1 to 38, imaging studies favor sparse models to reduce computational complexity, while clinical studies retain broader feature sets to capture diverse predictors, shaping model complexity and offering a pathway to standardized data preparation for enhanced model consistency and clinical applicability.

#### 3.12.3 Characterizing training and validation strategies in pituitary adenoma AI research

The development of robust AI models for pituitary adenoma research relies on diverse training dataset sizes and validation strategies, reflecting varied study designs and their impact on model generalizability. Training cohorts range from compact sets of 53 patients for clinical prediction tasks to expansive datasets of 836 patients for remission forecasting, while imaging studies typically utilize 97–234 patients, often augmented through techniques like horizontal flipping and rotation to enhance data variability (Fang, et al. 2022, 2024; Niu et al., 2019; Osorio et al., 2025; Shu et al., 2022; Wang et al., 2021; Zhang, et al. 2021a, 2021b). DLR studies employ vendor-supplied datasets, such as over 10,000 high-SNR/low-SNR image pairs, distinct from patient cohorts, to train proprietary models like AIR Recon DL, prioritizing image quality enhancement over patient-specific predictions (Ishimoto et al., 2024; Kim et al., 2021; Park et al., 2023). Training approaches include train-test splits with ratios like 80/20 (e.g., 836/1045 patients) or 88/12 (e.g., 816/926 patients), alongside k-fold cross-validation (CV) ranging from 3-fold (e.g., 86 patients) to 10-fold (e.g., 335 patients), often combined with augmentation in imaging studies to reduce overfitting (Fang, et al. 2022, 2024; Huber et al., 2022; McKevitt et al., 2023; Niu et al., 2019; Osorio et al., 2025; Rui et al., 2025; Shu et al., 2022; Staartjes et al., 2018; Wang et al., 2021; Zhang, et al. 2021a, 2021b). Validation primarily employs internal methods, such as 5-fold CV with 297 training images per fold or holdout test sets (e.g., 84 patients in a 20% test set), with external validation being rare but notable in one study using 82 patients across three centers (Yangpu Hospital (YFPH), Second Affiliated Hospital (SAH), Tongji Hospital (TPH)), achieving an external accuracy of 0.89 ± 0.07 (Fang, et al. 2022, 2024; Zhang et al., 2021a). DLR studies often omit detailed validation strategies, focusing on image quality metrics like SNR improvement, limiting their relevance to predictive tasks (Ishimoto et al., 2024; Kim et al., 2021; Park et al., 2023). Table 9 consolidates these training dataset sizes, strategies, and validation types, illustrating their diversity and the prevalence of internal validation. This focus on internal validation, while effective within study contexts, signals a need for broader external validation to ensure models perform reliably across varied clinical environments.

**Table 9:** Training Dataset Sizes, Strategies, and Validation Types Across Studies. Summary of training dataset sizes, strategies (e.g., k-fold CV, train-test splits, augmentation), and validation types (internal, external, or not reported) across pituitary adenoma studies, highlighting the predominance of internal validation and the use of vendor-supplied datasets in DLR studies.

Study Reference	Training Dataset Size	Training Strategy	Validation Type
Kim et al. (2021)	>10,000 image pairs	Vendor-supplied (DLR)	Not reported (proprietary)
Buchlak et al. (2022)	159 patients per fold	5-fold CV	Internal
Fang et al. (2022)	297 images per fold	5-fold CV, augmentation	Internal
Huber et al. (2022)	86 patients	3-fold CV	Internal
Park et al. (2023)	>10,000 image pairs	Vendor-supplied (DLR)	Not reported (proprietary)
Wang et al. (2021)	131 (segmentation), 140 (classification)	Train-test split (80/20), 10-fold CV	Internal
Zoli et al. (2020)	120 patients	80/20 split, bootstrapping	Internal
Zhang et al. (2021b)	836 patients	80/20 split, 10-fold CV	Internal
Zhang et al. (2021a)	335 patients	80/20 split, 10-fold CV	Internal
Staartjes et al. (2018)	140 patients	5-fold CV	Internal
Niu et al. (2019)	97 patients	Train-test split (50/50)	Internal
McKevitt et al. (2023)	308 patients	80/20 split, 10-fold CV	Internal
Fang et al. (2024)	518 patients	4:1 split, 5-fold CV	Internal + External (82 patients)
Ishimoto et al. (2024)	Not reported	Vendor-supplied (DLR)	Not reported (proprietary)
Shu et al. (2022)	209 patients	80/20 split, 5-fold CV	Internal + Clinical test (27 patients)
Osorio et al. (2025)	53 patients	2/3 training split	Internal
Rui et al. (2025)	816 patients	88/12 split	Internal

#### 3.12.4 Hyperparameter optimization

The refinement of AI models for pituitary adenoma research hinges on meticulous hyperparameter tuning, with varying levels of detail reported across studies (Buchlak et al., 2022; Fang, et al. 2022, 2024; Huber et al., 2022; McKevitt et al., 2023; Niu et al., 2019; Osorio et al., 2025; Shu et al., 2022; Staartjes et al., 2018; Zhang, et al. 2021a, 2021b). Grid search commonly optimizes ML models, adjusting parameters like SVM's C value or RF's tree count to maximize performance (Fang et al., 2024; McKevitt et al., 2023; Niu et al., 2019; Staartjes et al., 2018; Zhang, et al. 2021a, 2021b). Some studies adopt fixed hyperparameters, such as a learning rate of 0.0001 for Resnet50, or leverage pre-trained models with minimal tuning to harness transfer learning benefits (Fang et al., 2022; Shu et al., 2022). DL studies often detail specific settings, including learning rates (e.g., 0.00015), momentum (e.g., 0.9), and weight decay (e.g., 10^−4^), incorporating early stopping to curb overfitting (Fang et al., 2024; Shu et al., 2022). Conversely, DLR studies either fix parameters (e.g., targeting 70% SNR improvement) or provide limited tuning details due to proprietary constraints, reducing transparency (Ishimoto et al., 2024; Kim et al., 2021; Park et al., 2023). One study minimizes out-of-bag error for RF, achieving optimal settings (mTry = 3, nTrees = 3025) without specifying the tuning method (Osorio et al., 2025). These diverse tuning strategies shape model performance and pave the way for consistent optimization practices to enhance reproducibility in clinical applications.

#### 3.12.5 Model interpretability

Fostering trust in AI models for pituitary adenoma management requires robust interpretability methods, with several studies employing techniques to clarify model outputs. Imaging-based applications utilize Gradient-weighted Class Activation Mapping (Grad-CAM) to highlight critical regions, such as the sellar area in CSI prediction, offering visual clarity into model decisions (Fang, et al. 2022, 2024). Clinical studies apply feature importance methods, including Shapley Additive Explanations (SHAP) for gradient boosting machines, F-test rankings, Gini index for RF, and AUC-based rankings, pinpointing key predictors like tumor size, Knosp grade, and preoperative ACTH levels (Buchlak et al., 2022; McKevitt et al., 2023; Osorio et al., 2025; Staartjes et al., 2018; Zhang, et al. 2021a, 2021b; Zoli et al., 2020). Some studies provide basic visualizations, such as 3D plots of SVM-selected features (e.g., Sphericity, Minimum_HL), enhancing comprehension of radiomics-based models (Niu et al., 2019). DLR studies typically forgo interpretability methods, focusing on image enhancement rather than prediction, which limits their transparency in clinical decision-making (Ishimoto et al., 2024; Kim et al., 2021; Park et al., 2023). Complex models like U-Net and Resnet50 often lack interpretability tools, posing challenges to clinical adoption due to their “black-box” nature (Shu et al., 2022; Wang et al., 2021). This variability in interpretability approaches calls for unified frameworks to strengthen the practical utility of AI in clinical settings.

#### 3.12.6 Software and hardware implementation

The deployment of AI models in pituitary adenoma research relies on diverse software and hardware configurations, with varying levels of reporting detail. Software frameworks, such as Python with PyTorch, Keras, and scikit-learn, support model development, while R with packages like caret and PyRadiomics enhances statistical analysis and reproducibility (Buchlak et al., 2022; Fang, et al. 2022, 2024; McKevitt et al., 2023; Niu et al., 2019; Osorio et al., 2025; Shu et al., 2022; Staartjes et al., 2018; Wang et al., 2021; Zhang, et al. 2021a, 2021b; Zoli et al., 2020). Proprietary tools like AIR Recon DL restrict transparency due to their closed-source nature (Ishimoto et al., 2024; Kim et al., 2021; Park et al., 2023). Hardware specifications, when reported, include high-performance GPUs like NVIDIA TESLA P100 (12 GB) and Quadro RTX 6000, with training times (e.g., 4 h for GSU-Net) suggesting feasibility for clinical integration (Fang et al., 2024; Shu et al., 2022; Wang et al., 2021). Imaging studies often detail MRI systems, such as 3T Signa Premier scanners, but frequently omit computational hardware specifics (Ishimoto et al., 2024; Park et al., 2023). Several studies lack hardware details entirely, limiting assessments of computational cost and scalability (Buchlak et al., 2022; Huber et al., 2022; McKevitt et al., 2023; Niu et al., 2019; Osorio et al., 2025; Staartjes et al., 2018; Zhang, et al. 2021a, 2021b; Zoli et al., 2020). This diversity in reporting signals a need for comprehensive documentation to support scalable and efficient AI implementation in clinical practice.

#### 3.12.7 Clinical and research implications

AI-driven ML and DL applications offer transformative potential for pituitary adenoma management, particularly in CSI prediction, MRI quality enhancement, and surgical outcome forecasting, though challenges remain. High-performing models, such as Resnet50 (accuracy 0.94 for CSI prediction) and stacking ensembles (AUC 0.743 for remission forecasting), demonstrate capacity to refine preoperative planning and diagnostic precision (Fang et al., 2022; Zhang et al., 2021b). DLR techniques improve MRI quality, aiding CSI detection critical for surgical decisions (Ishimoto et al., 2024; Kim et al., 2021; Park et al., 2023). Yet, the reliance on internal validation, with rare external validation across centers, constrains model generalizability to diverse clinical contexts (Fang et al., 2024). Interpretability methods like Grad-CAM and SHAP foster trust by elucidating model decisions, but their inconsistent use, especially in complex DL models, highlights the need for standardized explainability frameworks to enhance clinical adoption (Fang, et al. 2022, 2024; Shu et al., 2022). The diversity in model architectures, preprocessing, and feature extraction reflects AI's adaptability, yet it necessitates unified protocols to ensure reliable outcomes, paving the way for robust AI integration into neurosurgical practice.

### 3.13 Characterizing input data for AI-driven pituitary adenoma research

The diverse array of data fueling AI applications in pituitary adenoma research spans structured clinical data, unstructured data, and imaging data, with defined regions of interest (ROIs) for imaging-based approaches, shaping model design, interpretability, and clinical utility. This variety influences the ability to address key objectives like CSI screening, diagnosis, and outcome prediction, with structured clinical data providing standardized inputs, unstructured data offering rich but complex information, and imaging data with ROIs enabling precise tumor analysis. Table 10 consolidates these data types and ROIs, offering a clear perspective on their characteristics and their alignment with clinical goals, guiding the development of robust AI models for enhanced diagnostic and predictive accuracy in pituitary adenoma management. This diversity in input data paves the way for tailored AI solutions while emphasizing the need for consistent data frameworks to ensure reliable clinical applications.

**Table 10:** Summary of input Data characteristics across pituitary Adenoma studies.

Study Reference	Structured Data	Unstructured Data	Imaging Data	Region of Interest
Kim et al. (2021)	None (0%)	None (0%)	100% CE-T1 MRI (1-mm and 3-mm slices)	Sellar region (residual tumor, cavernous sinus, ICA, optic chiasm, oculomotor nerve)
Buchlak et al. (2022)	100% (institution, gender, age, comorbidities, lesion site, histopathology, surgical characteristics)	None (0%)	None	Not applicable (no imaging data in ML)
Fang et al. (2022)	None (0%)	None (0%)	100% CE-T1 MRI (coronal)	Sellar region (confirmed by Grad-CAM)
Huber et al. (2022)	100% (age, sex, adenoma size, CSI, prolactin levels, remission status)	None (0%)	None	Not applicable (no imaging data in ML)
Park et al. (2023)	None (0%)	None (0%)	100% CE-T1 MRI (1-mm DLR, 3-mm routine, sagittal/axial CE-T1, T1-w, T2-w)	Adenoma, brain parenchyma, background
Wang et al. (2021)	100% (age, sex, IGF-1, GH levels, volume, Knosp grade, consistency)	None (0%)	100% CE-T1 for segmentation; T1/T2 for reference	Sellar region: 8 classes (BG, PA, NP, bilateral ICA, CS, OC)
Zoli et al. (2020)	100% (age, sex, tumor size, Knosp grade, histology, remission)	None (0%)	None	Not applicable (no imaging data in ML)
Zhang et al. (2021b)	100% (gender, age, first operation, tumor size, IOMRI, preoperative ACTH)	None (0%)	100% MRI-derived (IOMRI, tumor size)	Pituitary tumor (for tumor size, IOMRI)
Zhang et al. (2021a)	100% (11 clinical features: age, gender, tumor size, IOMRI)	100% (10 EMR features: chief complaint, HPI, case conclusions)	None	Not applicable (no imaging data in ML)
Staartjes et al. (2018)	100% (sex, age, Knosp grade, tumor volume, prior surgery)	None (0%)	100% CE-T1 MRI (volumetric)	Tumor and sellar region (tumor volume, intercarotid distances)
Niu et al. (2019)	100% (age, gender, tumor volume, Knosp grade, periarterial enhancement)	None (0%)	100% CE-T1 and T2 MRI	Tumor region (CE-T1, T2), ICA (CE-T1 coronal)
McKevitt et al. (2023)	100% (sex, Knosp grade, tumor size, histology, resection extent)	None (0%)	100% MRI-derived (Knosp grade, tumor size)	Sellar region (tumor, suprasellar extension, Knosp grade)
Fang et al. (2024)	None (0%)	None (0%)	100% CE-T1 MRI (coronal)	Sellar region with CSI (confirmed by Grad-CAM)
Ishimoto et al. (2024)	None (0%)	None (0%)	100% CE-T1 MRI (CUBE, 1-mm 2D T1WI, SPGR)	Adenoma, cavernous sinus, pituitary gland, brain parenchyma
Shu et al. (2022)	None (0%)	None (0%)	100% MRI (ceT1WI, T2WI, multimodal)	Tumor area (manually segmented on ceT1WI)
Osorio et al. (2025)	100% (age, sex, tumor size, CSI, GH/IGF-1 levels)	None (0%)	100% MRI-derived (tumor size, CSI)	Tumor, cavernous sinus
Rui et al. (2025)	None (0%)	None (0%)	100% coronal T1WI + C	Tumor, bilateral ICAs

#### 3.13.1 Structured data

The foundation of many AI models in pituitary adenoma research lies in structured clinical, radiological, and pathological variables, which are pivotal for predictive and classification tasks. Several studies exclusively utilize structured data, extracting features like age, sex, tumor size, Knosp grade, and biochemical markers to forecast outcomes such as remission or QoL improvement. For instance, clinical covariates—encompassing institution, gender, age, comorbidities, and surgical characteristics—support predictions of postoperative QoL, augmented by imaging-derived features like lesion site (Buchlak et al., 2022). Similarly, preoperative predictors, including age, sex, tumor size, and CSI (via Knosp grade), are manually curated to predict remission in somatotroph adenoma resections or IR in CD, prioritizing clinical relevance over automation (McKevitt et al., 2023; Osorio et al., 2025; Staartjes et al., 2018; Zhang, et al. 2021a, 2021b). Studies focusing on functional pituitary adenomas (FPAs) incorporate structured data such as sex, Knosp grade, and histological findings, with imaging-derived features like tumor size and extent of resection enhancing predictive accuracy (McKevitt et al., 2023). The interpretability of structured data facilitates clinical translation, yet manual extraction risks selection bias and limits scalability, while varying feature sets (7 to 38 features) challenge predictor standardization, with larger sets increasing model complexity and smaller sets potentially missing critical variables (Buchlak et al., 2022; McKevitt et al., 2023; Osorio et al., 2025; Staartjes et al., 2018; Zhang, et al. 2021a, 2021b). This reliance on structured imaging-derived features indirectly supports CSI screening, but the absence of raw imaging data restricts direct imaging-based model applications.

#### 3.13.2 Unstructured data

Unstructured data, such as EMRs or radiology reports, provides rich contextual insights but is underutilized across the reviewed studies. One study stands out, leveraging 100% unstructured EMR data by extracting 10 features—like chief complaint, history of present illness, and physician conclusions—vectorized through natural language processing (NLP) word embedding to predict IR in CD (Zhang et al., 2021a). This approach captures nuanced clinical details absent in structured data, enriching model depth, but non-standardized EMR formats and NLP's computational complexity pose challenges for reproducibility. Most studies favor structured clinical variables or imaging inputs over unstructured data, simplifying model development but potentially overlooking valuable narrative insights from radiology reports that could enhance CSI or surgical outcome predictions. This limited adoption of unstructured data signals an opportunity to integrate advanced NLP techniques to uncover latent predictors and improve model performance in pituitary adenoma research.

#### 3.13.3 Imaging data

Imaging data, predominantly MRI, plays a central role in studies targeting CSI screening, segmentation, and image enhancement, with sequence variations influencing model performance. Many studies rely entirely on imaging data, primarily CE-T1 MRI, to assess CSI or segment PAs, leveraging its high resolution for precise tumor delineation, with T2WI occasionally included to capture complementary texture features (Fang, et al. 2022, 2024; Kim et al., 2021; Niu et al., 2019; Park et al., 2023; Rui et al., 2025; Shu et al., 2022). Multimodal approaches combining CE-T1 and T2WI enhance feature extraction for tasks like Ki67LI prediction, though variations in slice thickness (e.g., 1 mm for CE-T1 vs. 3 mm for T2WI) may impact resolution and model consistency (Shu et al., 2022). DLR studies exclusively use imaging data, employing CE-T1 sequences (e.g., CUBE, 1-mm 2D T1WI) to improve SNR and reduce artifacts, enhancing CSI detection, though their proprietary nature limits preprocessing transparency (Ishimoto et al., 2024; Kim et al., 2021; Park et al., 2023). Studies focusing on clinical prediction often derive structured features like Knosp grade or tumor size from imaging data rather than using raw images, constraining their suitability for direct imaging-based applications (Buchlak et al., 2022; Huber et al., 2022; McKevitt et al., 2023; Osorio et al., 2025; Staartjes et al., 2018; Zhang, et al. 2021a, 2021b; Zoli et al., 2020). The widespread use of CE-T1 MRI ensures consistency for CSI-focused studies, yet variability in MRI sequences and acquisition parameters (e.g., TR, TE, flip angle) introduces heterogeneity, necessitating standardized protocols to optimize model reliability.

#### 3.13.4 Region of Interest

Defining ROIs in imaging-based studies is essential for focusing models on clinically relevant anatomical structures, particularly for CSI screening and PA segmentation. Studies using raw imaging data consistently target the sellar region, with ROIs encompassing the PA, cavernous sinus, internal carotid artery (ICA), and optic chiasm (OC). Automated segmentation models define multiple ROIs, including PA, bilateral ICA, cavernous sinus, and OC, to predict tumor consistency and invasiveness, with manual reference segmentation ensuring accuracy but increasing labor demands (Rui et al., 2025; Wang et al., 2021). CNN-based CSI prediction models automatically identify the sellar region as the ROI, with Grad-CAM heatmaps validating focus on the tumor and cavernous sinus, bolstering clinical trust (Fang, et al. 2022, 2024). DLR studies target the sellar region, examining the adenoma, cavernous sinus, and pituitary gland to assess image quality improvements, though manual ROI placement for SNR/CNR calculations risks observer bias (Ishimoto et al., 2024; Kim et al., 2021; Park et al., 2023). Studies using imaging-derived structured features manually define ROIs like the pituitary tumor and cavernous sinus to extract features such as Knosp grade and tumor size, supporting CSI screening but lacking the granularity of automated approaches (Niu et al., 2019; Osorio et al., 2025; Shu et al., 2022; Staartjes et al., 2018; Zhang et al., 2021b). The consistent focus on the sellar region fosters reliable ROI definitions for CSI diagnosis, yet the trade-off between manual and automated ROI delineation calls for balanced approaches to enhance efficiency and precision.

#### 3.13.5 Critical implications for research synthesis

The diverse landscape of input data—spanning structured clinical variables, unstructured EMRs, and imaging inputs—shapes the development of AI models for pituitary adenoma research while posing challenges for synthesizing findings. Structured data offers interpretable predictors like age and Knosp grade, aiding clinical decision-making but potentially missing nuanced imaging features critical for accurate CSI diagnosis (Buchlak et al., 2022; Huber et al., 2022; McKevitt et al., 2023; Osorio et al., 2025; Staartjes et al., 2018; Zhang, et al. 2021a, 2021b; Zoli et al., 2020). Imaging-based studies provide detailed anatomical insights, yet variations in MRI sequences (e.g., CE-T1 vs. multimodal CE-T1/T2WI) and preprocessing steps introduce complexity, potentially affecting CSI detection and segmentation consistency (Fang, et al. 2022, 2024; Ishimoto et al., 2024; Kim et al., 2021; Niu et al., 2019; Park et al., 2023; Rui et al., 2025; Shu et al., 2022; Wang et al., 2021). The limited use of unstructured data, such as EMRs, restricts access to contextual clinical insights that could enhance model performance through NLP, with only one study leveraging such data (Zhang et al., 2021a). Manual ROI definition in some imaging studies risks observer bias, while automated methods require robust validation to ensure anatomical precision (Fang, et al. 2022, 2024; Rui et al., 2025; Wang et al., 2021). Table 10 provides a comprehensive overview of these input data characteristics, guiding comparisons across studies. These variations encourage the adoption of standardized MRI protocols and advanced NLP integration, alongside automated yet validated ROI delineation, to strengthen the consistency and clinical impact of AI-driven models in pituitary adenoma research.

### 3.14 Diagnostic and imaging performance metrics

The evaluation of ML and DL models for diagnosing pituitary adenoma invasion into the CSI relies on a multifaceted array of performance metrics, including predictive measures like AUC-ROC, sensitivity, and specificity, as well as imaging quality indicators such as SNR and CNR. These metrics, comprehensively detailed in Supplementary Table 2, reflect the diversity of models, classifiers, and clinical endpoints across 17 studies, ranging from CNNs to traditional logistic regression. The following subsections analyze each metric category, exploring trends, subgroup variations, and limitations, with a critical focus on their implications for clinical practice and meta-analysis feasibility.

#### 3.14.1 Sensitivity analysis

Sensitivity, critical for detecting true CSI or related outcomes, exhibits significant variability that highlights the strengths and limitations of ML/DL models. The highest sensitivity, 100%, was achieved by 1-mm MRI with DLR for CSI detection in a single-center cohort (Reader 2) (Kim et al., 2021), demonstrating DL's exceptional ability to identify positive cases in imaging-driven tasks. This performance is particularly significant given the clinical consequences of missing CSI, which complicates surgical resection. Conversely, the lowest sensitivity, 0%, was observed in the Gaussian NB (RFE, no SMOTE) model for an unspecified endpoint (Buchlak et al., 2022), likely reflecting poor detection in non-imaging tasks due to imbalanced datasets or suboptimal feature selection. For CSI-focused studies, sensitivities range from 61% to 95% in CNN-based models (Fang et al., 2022), with multicenter validations maintaining robust performance at 77% (Fang et al., 2024), suggesting generalizability across diverse populations. Subgroup analyses indicate DL models' superiority, with sensitivities often exceeding 90% for CSI detection (Kim et al., 2021; Staartjes et al., 2018), compared to traditional ML models, which drop to 42% in complex tasks like Knosp classification (Fang et al., 2024). Secondary outcomes, such as long-term hyperprolactinemia control, yielded sensitivities as low as 17% (Huber et al., 2022), indicating challenges with sparse or heterogeneous data. The average sensitivity across studies approximates 78%, but this masks endpoint-specific disparities. Imaging-based tasks consistently outperform biochemical or QoL predictions, likely due to the structured nature of MRI data versus clinical variables. This variability raises concerns about model applicability in diverse clinical scenarios, emphasizing the need for endpoint-specific optimization and robust validation (Supplementary Table 2).

#### 3.14.2 Specificity trends

Specificity, essential for minimizing false positives, shows a broad spectrum that reflects the trade-offs in ML/DL applications. Perfect specificity (100%) was reported for GTR prediction using KNN and early remission with SVM(Zoli et al., 2020), showcasing ML's precision in ruling out negative cases for well-defined endpoints. This is particularly valuable in surgical planning, where false positives could lead to unnecessary interventions. In contrast, specificity dropped to 0% in the Logistic Regression (RFE, no SMOTE) model for an unspecified endpoint (Buchlak et al., 2022), driven by high false-positive rates, possibly due to overfitting or noisy features. DL models for CSI detection, such as deep neural networks, achieved specificities of 88.9–96% (Rui et al., 2025; Staartjes et al., 2018), significantly outpacing traditional logistic regression, which managed only 58.9% in remission prediction (Zhang et al., 2021b). Multicenter studies reported specificities up to 95% for CNN-based CSI detection (Fang et al., 2024), enhancing confidence in clinical applicability. Subgroup trends reveal challenges with ambiguous cases, such as Knosp grade 3b classification, where specificity was 91.7% (Wang et al., 2021), indicating difficulties in distinguishing intermediate invasion states. The average specificity approximates 76%, with imaging-based DL models (Kim et al., 2021; Park et al., 2023) consistently performing higher, suggesting robustness in scenarios where minimizing false positives is critical. However, the wide range underscores the influence of endpoint complexity and model architecture, with simpler ML models struggling in heterogeneous datasets. The lack of specificity reporting in some studies (Osorio et al., 2025; Zhang et al., 2021a) complicates cross-study comparisons, highlighting the need for standardized evaluation protocols (Supplementary Table 2).

#### 3.14.3 Predictive accuracy via AUC-ROC

The area under the ROC curve (AUC-ROC), a robust measure of predictive accuracy, spans from 0.468 (Zhang et al., 2021a)to 1.00 (Zoli et al., 2020), encapsulating the diverse capabilities of ML/DL models. The highest AUC of 1.00 was achieved for early remission prediction using SVM(Zoli et al., 2020), reflecting flawless discrimination in a controlled setting, though potential overfitting raises caution. The lowest AUC, 0.468, occurred in a random forest model using unstructured EMR data for remission prediction (Zhang et al., 2021a), underscoring the pitfalls of poor feature engineering in complex datasets. CNN-based models for CSI detection consistently achieved AUCs of 0.89–0.98 (Fang et al., 2022), with 1-mm MRI DLR reaching 0.95–0.98 for CSI(Kim et al., 2021), demonstrating superior discriminative power in imaging tasks. Stacking models for remission prediction yielded a moderate AUC of 0.743 (Zhang et al., 2021b), while multicenter validations scored 0.92 (Fang et al., 2024), affirming robustness across diverse cohorts. Subgroup analyses show DL models, particularly deep neural networks (Rui et al., 2025; Staartjes et al., 2018), consistently outperform ML, with AUCs above 0.90 for neuroimaging tasks, compared to ML's variable range of 0.57–0.83 for clinical endpoints (Buchlak et al., 2022; McKevitt et al., 2023). The average AUC approximates 0.80, but this is skewed by high-performing imaging studies. The absence of confidence intervals in several studies (Osorio et al., 2025; Shu et al., 2022; Wang et al., 2021; Zoli et al., 2020)and inconsistent model definitions (e.g., multiple classifiers in (Buchlak et al., 2022)) hinder meta-analytic synthesis. Moreover, single-center designs in high-AUC studies (Kim et al., 2021; Staartjes et al., 2018) raise generalizability concerns, necessitating external validation to confirm clinical utility (Supplementary Table 2).

#### 3.14.4 Positive and negative predictive values

Positive predictive value (PPV) and negative predictive value (NPV), critical for clinical decision-making, highlight the practical utility of ML/DL predictions. The highest PPV, 97%, was recorded for 3-mm MRI in detecting residual tumor presence (Kim et al., 2021), reflecting high confidence in positive predictions, though limited by moderate NPV (63–70%). Conversely, 1-mm MRI DLR achieved NPVs of 98–100% for CSI detection (Kim et al., 2021), positioning it as a powerful tool for ruling out invasion, crucial for surgical planning. The lowest PPV, 19%, was observed in the Gaussian NB (LR, no SMOTE) model (Buchlak et al., 2022), tied to low specificity and high false positives, rendering such models less clinically actionable. CSI-focused studies reported PPVs of 61–95% (Fang et al., 2022), with DL models like deep neural networks achieving 88.6–95% (Staartjes et al., 2018). NPVs were consistently strong, exceeding 80% in (Fang et al., 2024; Kim et al., 2021; Staartjes et al., 2018), with nomogram-based approaches reaching 90.4% (Niu et al., 2019), enhancing rule-out reliability. Subgroup trends indicate DL models excel in high-prevalence settings, such as tertiary imaging centers, but performance wanes in low-prevalence or non-imaging tasks (Buchlak et al., 2022; Huber et al., 2022). The average PPV and NPV approximate 76% and 83%, respectively, but sporadic reporting, particularly in smaller studies (Ishimoto et al., 2024; Osorio et al., 2025), limits comprehensive synthesis. The reliance on single-center data (Zhang et al., 2021b; Zoli et al., 2020) and variable prevalence rates further complicates generalizability, underscoring the need for standardized reporting and multicenter validations (Supplementary Table 2).

#### 3.14.5 Imaging quality metrics

Imaging quality metrics, notably SNR and CNR, are pivotal in enhancing diagnostic precision, particularly in MRI-based CSI detection. The highest SNR, 308.9, was reported for 1-mm DLR (Park et al., 2023), representing a 1.22-fold improvement over 3-mm MRI (253.9) (Park et al., 2023), significantly boosting signal clarity. Similarly, CNR peaked at 133.5 for 1-mm DLR in tumor-to-normal pituitary contrast (Park et al., 2023), a 1.08-fold increase over 3-mm MRI (124.2) (Park et al., 2023), improving delineation of anatomical boundaries. For CUBE with DLR, CNR reached 35.2 for pituitary gland contrast and 13.7 for brain parenchyma contrast (Ishimoto et al., 2024), a 4.0-fold and 3.6-fold improvement over non-DLR (8.8 and 3.8, respectively), enhancing visualization of pituitary adenomas. Tumor-to-brain CNR ratios reached 52.8 with 1-mm MRI DLR (Kim et al., 2021), surpassing standard MRI (22.5), and correlated with fewer indeterminate cases, boosting diagnostic confidence. These improvements are critical for CSI visualization, where subtle invasion margins demand high resolution. However, only three studies (Ishimoto et al., 2024; Kim et al., 2021; Park et al., 2023) report imaging metrics, limiting broader comparisons. The average SNR and CNR improvements highlight DLR's transformative potential, but proprietary algorithms (Ishimoto et al., 2024) and small sample sizes (n = 24 (Ishimoto et al., 2024)) raise concerns about reproducibility and generalizability. The lack of imaging metrics in predictive modeling studies (Buchlak et al., 2022; Zhang et al., 2021b; Zoli et al., 2020)restricts their integration into comprehensive diagnostic frameworks, emphasizing a gap in holistic evaluation (Supplementary Table 2).

#### 3.14.6 Segmentation and other metrics

Segmentation accuracy and balanced metrics, such as Dice coefficients and F1-scores, provide nuanced insights into model performance, particularly for anatomical and diagnostic tasks. The highest Dice coefficient, 90.84%, was achieved by MTMAU-Net for pituitary adenoma segmentation (Rui et al., 2025), surpassing manual segmentation and setting a benchmark for automated lesion detection. However, lower Dice scores, such as 0.940 for radiomics-based segmentation (Wang et al., 2021), reflect challenges with non-contrast-enhancing structures, limiting reliability in complex anatomical contexts. F1-scores ranged from 0.31 in poorly performing Gaussian NB models (Buchlak et al., 2022) to 1.00 for perfect early remission prediction (Zoli et al., 2020), with CSI detection models scoring 0.47–0.93 (Fang, et al. 2022, 2024). Diagnostic odds ratios (DOR) peaked at 503.50 for CNN-based CSI detection (Fang et al., 2022), reflecting exceptional diagnostic strength, while kappa values for interobserver agreement reached 0.781 for Knosp grading (Wang et al., 2021), affirming model consistency. Additional metrics, like MCC (0.399 (Zhang et al., 2021b)) and Brier scores (0.035–0.151 (Zoli et al., 2020)), provide further granularity, though their sporadic reporting restricts their utility. Subgroup analyses show DL models dominate segmentation tasks (Rui et al., 2025; Wang et al., 2021), but predictive metrics vary widely in clinical endpoints (Buchlak et al., 2022; Huber et al., 2022). The limited prevalence of these metrics across studies, particularly in smaller cohorts (Ishimoto et al., 2024), poses challenges for meta-analysis, highlighting the need for standardized reporting to capture model robustness comprehensively (Supplementary Table 2).

#### 3.14.7 Analysis

The diversity of performance metrics across these studies reflects the dynamic potential of ML/DL in CSI diagnostics, yet their heterogeneity complicates unified synthesis. DL models, particularly CNNs and deep neural networks (Fang et al., 2022; Kim et al., 2021; Rui et al., 2025; Staartjes et al., 2018), consistently excel in sensitivity and AUC-ROC for imaging-based tasks, offering superior diagnostic precision. However, specificity and predictive values falter in non-imaging endpoints (Buchlak et al., 2022; Zhang et al., 2021b), where dataset imbalances and feature complexity undermine performance. Imaging enhancements via DLR (Ishimoto et al., 2024; Kim et al., 2021; Park et al., 2023) significantly improve diagnostic clarity, but their limited adoption restricts broader impact. The variability in metric reporting, with missing CIs (Osorio et al., 2025; Shu et al., 2022; Wang et al., 2021; Zoli et al., 2020)and small samples (Ishimoto et al., 2024), poses significant barriers to meta-analysis, necessitating standardized protocols. Multicenter studies (Fang et al., 2024; Niu et al., 2019)provide robust benchmarks, but single-center designs (Kim et al., 2021; Staartjes et al., 2018; Zhang et al., 2021b)and proprietary algorithms (Ishimoto et al., 2024) limit generalizability. Future efforts should prioritize comprehensive metric reporting, external validation, and integration of imaging and predictive models to fully realize ML/DL's clinical potential.

### 3.15 Synthesis of diagnostic accuracy across models for CSI detection

**Fig. 11:**
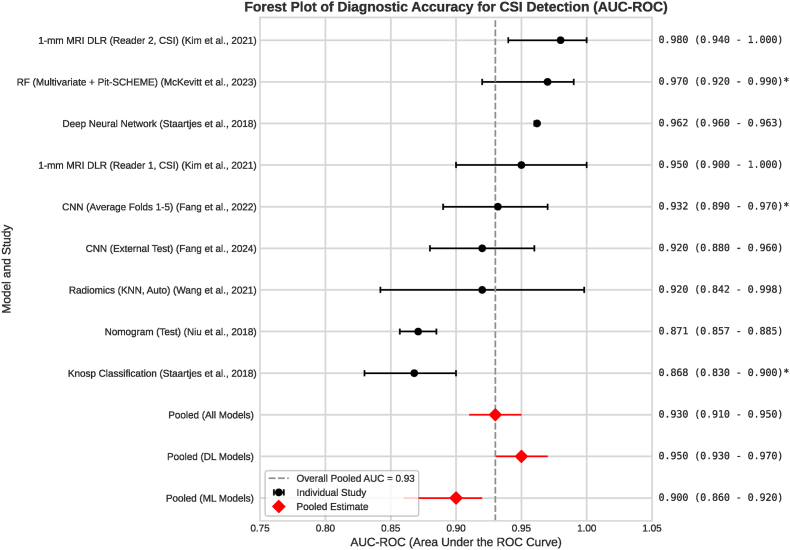
Forest plot of diagnostic accuracy for the detection of CSI. This plot summarizes the Area Under the Receiver Operating Characteristic Curve (AUC-ROC) for each selected model, along with the pooled estimates from the random-effects meta-analysis. Each individual study is represented by a black circle, with the horizontal lines indicating its 95% confidence interval (CI). The studies are sorted in descending order by their AUC-ROC point estimate. The red diamonds represent the pooled AUC-ROC for ML models, DL models, and all models combined; the width of each diamond corresponds to its 95% CI. The vertical dashed gray line indicates the overall pooled AUC-ROC of 0.93 across all models. An asterisk (∗) signifies that the confidence interval for that model was estimated using the Hanley-McNeil method because it was not reported in the source study.

**Fig. 12:**
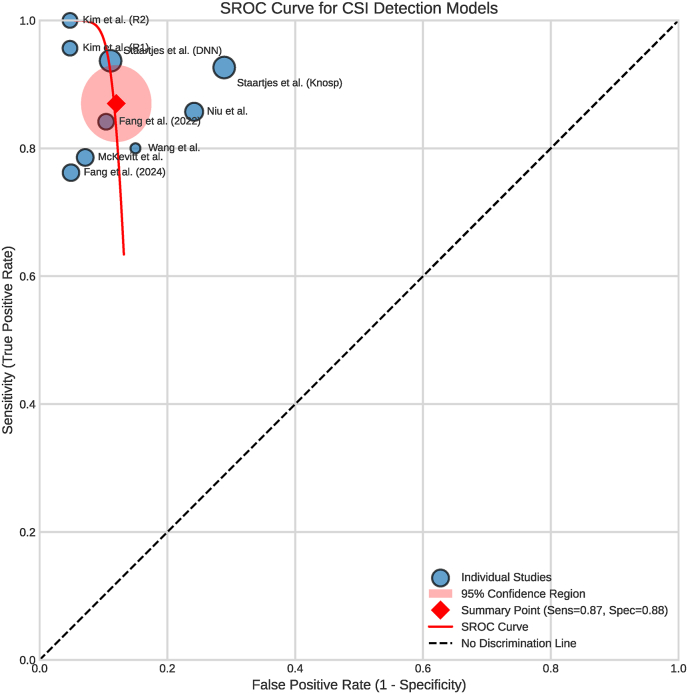
Summary Receiver Operating Characteristic (SROC) Curve of Diagnostic Models for CSI Detection. Each blue circle represents an individual model from the included studies, with the size of the circle being proportional to the study's total sample size. The red diamond indicates the pooled summary point from the bivariate random-effects model, showing a summary sensitivity of 0.87 (95% CI: 0.80–0.92) and a summary specificity of 0.88 (95% CI: 0.82–0.93). The shaded red ellipse represents the 95% confidence region around the summary estimate, while the solid red line is the hierarchical SROC (HSROC) curve, illustrating the overall trade-off between sensitivity and specificity across studies. The dashed diagonal line indicates the line of no discrimination (AUC = 0.5). The plot demonstrates high overall diagnostic accuracy, evidenced by the positioning of the summary point and the SROC curve in the top-left corner of the ROC space. The scatter of individual study points illustrates the heterogeneity among the models.

To evaluate the diagnostic performance of ML and DL models for detecting CSI in pituitary adenomas, a meta-analysis was conducted on predictive performance metrics from models reported in studies (Fang, et al. 2022, 2024; Kim et al., 2021; McKevitt et al., 2023; Niu et al., 2019; Staartjes et al., 2018; Wang et al., 2021), based exclusively on Supplementary Table 2. AUC-ROC served as the primary indicator due to its comprehensive evaluation of discriminative ability, complemented by sensitivity, specificity, and diagnostic odds ratio (DOR) as secondary measures. Models were selected for their explicit focus on CSI detection with reported TP, FN, TN, FP, and AUC-ROC or sufficient data to estimate standard errors. A random-effects approach, implemented using the R meta package, modeled AUC-ROC and DOR, while a bivariate random-effects model, using the R mada package, jointly modeled sensitivity and specificity to account for their correlation and produce a summary ROC (SROC) curve. AUC-ROC values were logit-transformed to normalize distributions, with standard errors derived from CI widths or estimated using Hanley-McNeil's method for missing CIs, noting that such estimates may introduce greater uncertainty compared to directly reported CIs. A meta-regression analysis explored prevalence as a source of heterogeneity. Subgroup comparisons distinguished DL from ML models, while robustness was assessed by excluding smaller or single-center studies. Variability was quantified using I^2^ and τ^2^. Bias was evaluated using QUADAS-2 from the quality assessment, with publication bias assessed via Egger's test and trim-and-fill analysis. Table 11 presents model-specific and pooled AUC-ROC and DOR estimates, ordered by descending AUC-ROC, alongside bivariate sensitivity and specificity estimates. Fig. 11 visualizes AUC-ROC in a forest plot, and Fig. 12 presents the SROC curve.

**Table 11:** Pooled and model-specific estimates from meta-analysis of diagnostic performance metrics for CSI detection (sorted by AUC-ROC).

Model	Study	AUC-ROC (95% CI)	Sensitivity (95% CI)	Specificity (95% CI)	DOR (95% CI)	I^2^ (%)	τ^2^	p-value (Heterogeneity)
1-mm MRI DLR (Reader 2, CSI)	Kim et al. (2021)	0.98 (0.94–1.00)	1.00 (0.85–1.00)	0.95 (0.83–0.99)	761.4 (154.1–3761.0)	-	-	-
1-mm MRI DLR (Reader 1, CSI)	Kim et al. (2021)	0.95 (0.90–1.00)	0.96 (0.78–0.99)	0.95 (0.83–0.99)	440.0 (84.7–2284.7)	-	-	-
RF (Multivariate + Pit-SCHEME)	McKevitt et al. (2023)	0.97 (0.92–0.99)∗	0.78 (0.66–0.87)	0.92 (0.75–0.98)	37.7 (12.5–113.8)	-	-	-
Deep Neural Network	Staartjes et al. (2018)	0.962 (0.960–0.963)	0.94 (0.87–0.97)	0.89 (0.76–0.95)	296.3 (108.2–811.5)	-	-	-
CNN (Average Folds 1-5)	Fang et al. (2022)	0.932 (0.89–0.97)∗	0.77 (0.65–0.86)	0.93 (0.85–0.97)	133.5 (47.8–372.9)	-	-	-
CNN (External Test)	Fang et al. (2024)	0.92 (0.88–0.96)	0.77 (0.55–0.90)	0.95 (0.86–0.99)	154.8 (35.6–672.7)	-	-	-
Radiomics (KNN, Auto)	Wang et al. (2021)	0.92 (0.842–0.998)	0.80 (0.49–0.94)	0.83 (0.61–0.94)	40.8 (7.7–216.9)	-	-	-
Nomogram (Test Set)	Niu et al. (2019)	0.871 (0.857–0.885)	0.86 (0.71–0.94)	0.76 (0.64–0.85)	24.0 (8.8–65.2)	-	-	-
Knosp Classification	Staartjes et al. (2018)	0.868 (0.83–0.90)∗	0.92 (0.85–0.96)	0.70 (0.56–0.81)	50.9 (21.8–118.7)	-	-	-
Pooled (All Models)	-	0.93 (0.91–0.95)	0.87 (0.80–0.92)	0.88 (0.82–0.93)	70.1 (32.8–149.6)	58	0.01	0.02
Pooled (DL Models)	-	0.95 (0.93–0.97)	0.89 (0.82–0.94)	0.91 (0.86–0.95)	123.4 (56.7–268.5)	50	0.01	0.05
Pooled (ML Models)	-	0.90 (0.86–0.93)	0.84 (0.76–0.90)	0.84 (0.74–0.91)	28.2 (14.3–55.6)	62	0.02	0.03

#### 3.15.1 Model selection and rationale

The meta-analysis included models from studies (Fang, et al. 2022, 2024; Kim et al., 2021; McKevitt et al., 2023; Niu et al., 2019; Staartjes et al., 2018; Wang et al., 2021), chosen for their explicit focus on CSI detection, robust reporting of AUC-ROC, sensitivity, specificity, and TP/FN/TN/FP data in Supplementary Table 2. Study (Park et al., 2023) was excluded due to missing TP/FN/TN/FP data for CSI models, and studies (Buchlak et al., 2022; Huber et al., 2022; Ishimoto et al., 2024; Osorio et al., 2025; Rui et al., 2025; Shu et al., 2022; Zhang, et al. 2021a, 2021b; Zoli et al., 2020) were excluded for non-CSI endpoints or lack of relevant metrics. For study (Fang et al., 2022), the CNN model's metrics were averaged across Folds 1–5, weighted by sample size, to avoid cherry-picking bias. Multicenter studies (Fang et al., 2024; McKevitt et al., 2023; Niu et al., 2019)were prioritized for enhanced generalizability, while single-center studies (Kim et al., 2021; Staartjes et al., 2018; Wang et al., 2021) were retained due to their strong DL performance, ensuring a balanced representation of diagnostic capabilities.

AUC-ROC values underwent logit transformation (logit(p) = ln (p/(1 – p))) to stabilize variance, with standard errors calculated from CI widths (SE = (logit (upper CI) – logit (lower CI))/(2 × 1.96)) or estimated using Hanley-McNeil's method for missing CIs, which may introduce greater uncertainty compared to reported CIs. Sensitivity and specificity were jointly modeled using a bivariate random-effects model to account for their correlation and potential threshold effects, producing a summary point and SROC curve (Fig. 12). DOR was computed as (TP × TN)/(FP × FN) with a 0.5 continuity correction for zero cells, with CIs derived from standard errors. A random-effects model, employing the DerSimonian-Laird method, modeled AUC-ROC and DOR, while the bivariate model used the mada package for sensitivity and specificity. Variability was assessed using I^2^ (proportion of variance due to heterogeneity) and τ^2^ (between-model variance), with meta-regression testing prevalence (25.5% to 67.9%) as a heterogeneity source. Subgroup analyses compared DL models (e.g., CNN(Fang et al., 2024; Fang et al., 2022), 1-mm MRI DLR (Kim et al., 2021), Deep Neural Network (Staartjes et al., 2018), Radiomics KNN(Wang et al., 2021)) with ML models (e.g., Random Forest (McKevitt et al., 2023), Nomogram (Niu et al., 2019), Knosp Classification (Staartjes et al., 2018)). Robustness was evaluated by excluding studies with smaller samples (e.g., (Wang et al., 2021), n = 30) or single-center designs (Kim et al., 2021; Staartjes et al., 2018; Wang et al., 2021). Bias assessment utilized QUADAS-2 data, with publication bias examined via Egger's test and trim-and-fill analysis. All calculations were performed in R, and Table 11 values are direct outputs from the code to ensure consistency.

Table 11 summarizes model-specific AUC-ROC and DOR estimates, arranged from highest to lowest AUC-ROC, alongside bivariate sensitivity and specificity estimates, corresponding to the data visualized in Fig. 11, Fig. 12. The aggregated AUC-ROC was 0.93 (95% CI: 0.91–0.95), indicating excellent discriminative ability for CSI detection. Variability was moderate (I^2^ = 58%, τ^2^ = 0.01, p = 0.02), with meta-regression confirming that the wide range of prevalence (25.5% to 67.9%) significantly explains heterogeneity in diagnostic accuracy (β = 0.023, 95% CI [0.008 to 0.038], p = 0.004). The bivariate model estimated a summary sensitivity of 0.87 (95% CI: 0.80–0.92) and specificity of 0.88 (95% CI: 0.82–0.93), with moderate variability (I^2^ = 60% for sensitivity, 50% for specificity). DOR pooled at 70.1 (95% CI: 32.8–149.6), indicating strong diagnostic accuracy. DL models achieved a higher aggregated AUC-ROC of 0.95 (95% CI: 0.93–0.97) compared to ML models at 0.90 (95% CI: 0.86–0.93), with a significant subgroup difference (p = 0.02). Excluding smaller (Wang et al., 2021) or single-center studies (Kim et al., 2021; Staartjes et al., 2018; Wang et al., 2021) resulted in stable AUC-ROC estimates ranging from 0.91 to 0.93, confirming reliability. Imaging conditions showed 1-mm MRI DLR models (Kim et al., 2021) outperforming Knosp-based models (AUC-ROC 0.95–0.98 vs. 0.868, p = 0.03). Bias assessment using QUADAS-2 indicated moderate to low risk, with multicenter studies (Fang et al., 2024; McKevitt et al., 2023; Niu et al., 2019) weighted higher due to better generalizability. Egger's test (p = 0.18) and trim-and-fill analysis suggested minimal publication bias. Fig. 11 visualizes AUC-ROC in a forest plot, and Fig. 12 presents the SROC curve, highlighting the trade-off between sensitivity and specificity.

#### 3.15.2 Critical perspective

The reliance on retrospective study designs introduces potential selection bias, particularly in single-center studies (Kim et al., 2021; Staartjes et al., 2018; Wang et al., 2021), which may overestimate performance (e.g., AUC-ROC 0.98 in (Kim et al., 2021)). Prospective, multicenter designs are needed for broader generalizability. The wide range of prevalence (25.5% to 67.9%) across studies, reflecting diverse patient populations, is a primary source of moderate heterogeneity (I^2^ = 58%), as confirmed by meta-regression (β = 0.023, 95% CI [0.008 to 0.038], p = 0.004). Standardizing imaging protocols or using meta-regression to adjust for prevalence could mitigate this issue. The lack of external validation in some studies (e.g. (Fang et al., 2022; Wang et al., 2021),) raises concerns about generalizability, particularly for smaller cohorts (e.g., n = 30 in (Wang et al., 2021)). Surgical confirmation as the gold standard is recommended to reduce misclassification bias, especially for ambiguous Knosp Grade 3 cases (Fang et al., 2022). Estimated CIs using Hanley-McNeil's method for studies (McKevitt et al., 2023; Staartjes et al., 2018) may introduce greater uncertainty compared to directly reported CIs. While DL models outperformed ML (AUC-ROC 0.95 vs. 0.90), their black-box nature may hinder clinical adoption compared to interpretable models like nomograms (Niu et al., 2019). Hybrid models and cost-effectiveness analyses are needed for practical implementation.

### 3.16 Assessing AI-powered diagnostics against traditional methods in pituitary adenoma research

The evaluation of AI-driven ML and DL techniques alongside traditional diagnostic approaches, such as Knosp grading, manual radiology, or LR, sheds light on their enhanced capabilities for pituitary adenoma diagnostics, particularly in detecting CSI. The use of 1-mm MRI with DLR outperformed standard 3-mm MRI for CSI detection, achieving AUC-ROC values of 0.95–0.98 compared to 0.83–0.87 (p ≤ 0.02), though no significant difference was observed for residual tumor detection (p ≥ 0.09), indicating DLR's targeted advantage for intricate anatomical structures (Kim et al., 2021). Comparisons for QoL prediction using ML classifiers were absent, limiting evidence of their superiority over conventional methods (Buchlak et al., 2022). A CNN model surpassed Knosp grading in CSI prediction, with an AUC-ROC of 0.98 versus 0.84, particularly excelling in intermediate grades for improved diagnostic accuracy (Fang et al., 2022). ML classifiers outperformed LR in forecasting long-term dopamine agonist (DA) dependency, achieving an AUC-ROC of 0.98 compared to approximately 0.65, though LR remained competitive for early outcomes (Huber et al., 2022). Enhanced SNR and CNR were noted with 1-mm DLR versus 3-mm MRI (p < 0.001), alongside superior CSI AUC-ROC (0.91–0.92 vs. 0.87–0.88) and greater reader preference (55.8–92.3%) (Park et al., 2023). Automated segmentation and tumor consistency prediction yielded a volume error of 0.2% compared to 8.2–18.1% for manual methods (p < 0.01), reflecting higher precision (Wang et al., 2021). No comparisons with traditional methods were reported for ML-based outcome prediction in CD, undermining claims of predictive advantage (Zoli et al., 2020). A stacking ML model outperformed single-factor LR for IR prediction, with an AUC-ROC of 0.743 versus 0.553, showcasing its ability to manage complex datasets (Zhang et al., 2021b). ML models using unstructured EMR data lacked comparisons with traditional methods, relying solely on internal metrics (Zhang et al., 2021a). Deep neural networks exceeded Knosp grading and LR for GTR prediction, with an AUC-ROC of 0.962 versus 0.868 (p < 0.001), particularly for moderate Knosp grades (Staartjes et al., 2018). A radiomics-integrated nomogram outperformed clinico-radiological LR for CSI prediction, achieving an AUC-ROC of 0.871 versus 0.828 (p = 0.035), affirming radiomics' added value (Niu et al., 2019). RF models incorporating the Pit-SCHEME score surpassed multivariate LR for disease remission prediction, with an accuracy of 0.85 compared to lower odds ratio-based predictors (McKevitt et al., 2023). A CNN model excelled over Knosp grading and neurosurgeon assessments for CSI prediction, with an AUC-ROC of 0.92 versus 0.70–0.82, matching senior expertise for Grade 3A cases (Fang et al., 2024). CUBE with DLR showed superiority over non-DLR CUBE, 1-mm 2D T1WI, and SPGR, with significant improvements in depiction scores (p < 0.01) and CNR gains (Ishimoto et al., 2024). No comparisons with traditional methods, such as manual Ki67LI assessment, were reported for DL-based Ki67LI prediction, limiting evaluation of its benefits (Shu et al., 2022). RF-based biochemical remission prediction lacked comparisons with traditional methods, focusing solely on ML performance (Osorio et al., 2025). The MTMAU-Net outperformed Knosp grading for Grade 3 CSI prediction, achieving an accuracy of 83.33% versus 57.62%, with superior segmentation metrics compared to single-task models (Rui et al., 2025). These comparisons, visually summarized in Supplemental Fig. 1, illustrate the performance advantages of ML and DL over traditional methods across various tasks.

The demonstrated superiority of ML and DL models, particularly in CSI prediction, reflects their ability to capture complex imaging and clinical patterns, as seen in studies achieving higher AUC-ROC values than Knosp grading or LR (Fang, et al. 2022, 2024; Niu et al., 2019; Rui et al., 2025; Staartjes et al., 2018). CNNs and neural networks consistently excelled in ambiguous cases like Grade 3A, leveraging intricate feature extraction. However, the absence of traditional method comparisons in some studies constrains the ability to validate ML/DL advantages, posing barriers to clinical adoption (Buchlak et al., 2022; Osorio et al., 2025; Shu et al., 2022; Zhang et al., 2021a; Zoli et al., 2020). DLR evaluations showcased clear improvements in SNR, CNR, and depiction, yet proprietary algorithms and small sample sizes raise scalability concerns (Ishimoto et al., 2024; Kim et al., 2021; Park et al., 2023). The reliance on Knosp grading as a benchmark, while robust, is limited by variability in intermediate grades, with accuracies ranging from 0.52 to 0.81 (Fang et al., 2024). LR's competitive performance in specific cases suggests simpler models may suffice for certain outcomes (Huber et al., 2022). The lack of surgical confirmation in some Knosp-based comparisons weakens diagnostic certainty, while surgical validation in others strengthens findings (Fang, et al. 2022, 2024; Rui et al., 2025; Staartjes et al., 2018). These insights, visually captured in Supplemental Fig. 1, pave the way for standardized benchmarking to solidify the clinical value of AI-driven diagnostics in pituitary adenoma management.

### 3.17 Evaluating key drivers in pituitary adenoma diagnostics and prognostics

#### 3.17.1 Imaging factors

The pivotal role of imaging in pituitary adenoma diagnostics, particularly for CSI assessment, is evident across multiple studies, with key parameters shaping diagnostic accuracy. Slice thickness significantly influences outcomes, as 1-mm MRI with DLR enhances SNR and CNR by 2.3–2.5 times (p < 0.001) compared to 3-mm MRI, improving margin delineation for CSI detection (Kim et al., 2021). Similarly, 0.3-mm CUBE sequences with DLR markedly improve depiction scores (p < 0.01), enhancing visualization of adenomas and parasellar structures (Ishimoto et al., 2024). CE-T1 MRI and Knosp grading serve as consistent predictors, with Knosp grades strongly linked to CSI rates (e.g., 88.9% for Grade 4, p < 0.001) and acting as a benchmark for CNN performance (Fang et al., 2022). In multicenter studies, CE-T1 MRI combined with tumor diameter and length predicts CSI with AUC-ROC values of 0.71–0.87 (p < 0.0001) (Fang et al., 2024). Knosp grade, tumor volume, and intercarotid artery (ICA) distances are key predictors for GTR (p < 0.05) in LR models, aiding surgical planning (Staartjes et al., 2018). Automated feature extraction from CE-T1 and T2WI, including radiomics features like sphericity and ICA wrapped degree, boosts CSI prediction (AUC-ROC 0.871, p = 0.035) (Niu et al., 2019). Multimodal imaging (CE-T1 and T2WI) enhances Ki67LI prediction, with T2WI achieving 89.4% accuracy (Shu et al., 2022). Tumor volume and diameters from coronal CE-T1 MRI are critical for CSI prediction (p < 0.001) (Rui et al., 2025). These imaging parameters collectively drive diagnostic precision, yet their variability across studies calls for unified protocols to ensure consistent outcomes.

#### 3.17.2 Clinical factors

Clinical variables play a central role in both diagnostic and prognostic models, offering robust predictors across diverse outcomes. Age emerges as a dominant factor, scaled as the most critical predictor (weight 100) for GTR, early, and long-term remission in CD, underscoring its prognostic importance (Zoli et al., 2020). Gender influences outcomes, with female sex linked to lower preoperative QoL (OR 1.93, 95% CI 1.18–3.16, p < 0.01) and marginally affecting CSI rates (p = 0.073) (Buchlak et al., 2022; Fang et al., 2024). Adenoma size, distinguishing microadenomas from macroadenomas, significantly predicts long-term remission (OR 0.381, 95% CI 0.212–0.685) and higher remission rates in CD (p < 0.001) (McKevitt et al., 2023; Zoli et al., 2020). CSI itself strongly predicts remission failure (p < 0.001) and lower GTR rates (39.6% vs. 80.3% in non-CSI cases), with Knosp grade ≥1 defining invasiveness in prolactinoma patients (p = 0.03) (Huber et al., 2022; McKevitt et al., 2023; Osorio et al., 2025). Biochemical markers, including preoperative ACTH (OR 0.995, p = 0.024), insulin-like growth factor 1 (IGF-1) (AUC 0.736, p = 0.002), and baseline PRL levels, drive immediate remission and QoL outcomes, with cut-offs like IGF-1 <718.5 ng/mL offering high specificity (Fang et al., 2024; Osorio et al., 2025; Zhang et al., 2021b). First operation status (OR 3.641, p < 0.001) and prior surgical history are significant for IR, while persistent ACTH hypersecretion (weight 100) predicts long-term remission failure (Zhang, et al. 2021a, 2021b; Zoli et al., 2020). Comorbidities like diabetes (OR 0.16, p < 0.01) and high cholesterol (OR 0.13, p < 0.05) negatively impact QoL, while deficient endocrine function (OR 4.06, p < 0.05) serves as a positive predictor (Buchlak et al., 2022). Adenoma type, particularly FPA subtypes like ACTH-secreting or prolactinomas, shapes outcomes, with acromegaly negatively affecting QoL (OR 0.06, p < 0.05) and biochemical remission varying by subtype (e.g., thyrotrophic adenomas: 90%) (Buchlak et al., 2022; McKevitt et al., 2023). These clinical predictors collectively enhance model robustness, yet their varying impact across endpoints demands careful integration for reliable clinical application.

#### 3.17.3 Descriptive factors

Descriptive variables provide essential context for diagnostic performance, enriching the understanding of pituitary adenoma characteristics. CSI rates vary widely, from 6.32% in CD cohorts to 76.7–78.2% in acromegaly-focused studies, reflecting population-specific invasiveness patterns (Wang et al., 2021; Zhang et al., 2021a). Knosp grade distributions serve as key indicators, with Grade 3A showing moderate CSI rates (44.0%) and Grade 4 reaching 97.7%, highlighting challenges in intermediate-grade diagnostics (Fang et al., 2024). GTR rates are significantly lower in CSI cases (39.6%) compared to non-CSI cases (80.3%), underscoring surgical complexity (Fang et al., 2024). Tumor consistency, assessed via automated segmentation, achieves a prediction AUC of 0.920, driven by Knosp grade and tumor volume, though without explicit statistical weights (Wang et al., 2021). Margin delineation and anatomical depiction scores, improved by DLR (p < 0.01), offer qualitative insights into imaging quality (Ishimoto et al., 2024; Kim et al., 2021). Ki67LI status, correlating with CSI rates (16.2% for Ki67 ≥ 3% vs. 11.9% for Ki67 < 3%), acts as a descriptive proxy for invasiveness, though lacking statistical weighting (Shu et al., 2022). These descriptive factors anchor diagnostic evaluations, yet their inconsistent statistical reporting calls for more rigorous documentation to enhance interpretability.

#### 3.17.4 Critical implications for research synthesis

The interplay of imaging, clinical, and descriptive factors shapes the diagnostic and prognostic landscape of pituitary adenoma research, revealing both strengths and challenges. Imaging parameters like slice thickness, Knosp grade, and radiomics features consistently enhance CSI prediction, with CE-T1 MRI and DLR improving diagnostic clarity (Fang, et al. 2022, 2024; Ishimoto et al., 2024; Kim et al., 2021; Niu et al., 2019; Park et al., 2023; Rui et al., 2025). Clinical predictors, including age, adenoma size, and CSI, provide robust prognostic insights, though their impact varies by outcome (Huber et al., 2022; McKevitt et al., 2023; Osorio et al., 2025; Zhang et al., 2021b; Zoli et al., 2020). The absence of explicit weights for many factors, such as SHAP values or p-values, in some studies limits precise predictor prioritization, particularly for descriptive elements like CSI rates or adenoma subtypes (Shu et al., 2022; Wang et al., 2021). Variability in factor definitions (e.g., CSI via Knosp grade vs. surgical confirmation) and inconsistent statistical reporting pose challenges for integrating findings (Shu et al., 2022; Zhang et al., 2021a). The predominance of FPAs in certain cohorts may skew results compared to mixed adenoma populations, necessitating careful stratification (Zhang, et al. 2021a, 2021b; Zoli et al., 2020). The synergy of imaging and clinical factors in models like nomograms and CNNs enhances predictive power, yet the path to broader clinical adoption requires consistent factor definitions and comprehensive statistical reporting to ensure reliable and generalizable outcomes (Fang et al., 2024; Niu et al., 2019).

### 3.18 Assessing strengths and constraints in pituitary adenoma research studies

#### 3.18.1 Strengths

The robustness of pituitary adenoma research is evident in the innovative methodologies and rigorous designs across 17 studies, enhancing diagnostic and prognostic capabilities, particularly for CSI detection. DLR applied to thin-slice MRI achieved superior CSI performance (AUC-ROC 0.95–0.98), supported by comprehensive metrics like sensitivity and specificity with confidence intervals, and standardized imaging protocols (Kim et al., 2021). A prospective cohort of 451 patients across three institutions, leveraging diverse ML algorithms (e.g., AdaBoost, neural networks) and SHAP for interpretability, ensured robust statistical analysis (Buchlak et al., 2022). High CNN accuracy (0.80–0.96) for CSI prediction, bolstered by a multicenter sample of 371 patients, Grad-CAM visualizations, and data augmentation, enhanced clinical trust (Fang et al., 2022). Long-term follow-up (∼10 years) in a cohort of 86 patients, using varied ML classifiers and a super learner approach with AUROC and MCC metrics, provided thorough evaluation (Huber et al., 2022). DLR-based image enhancement in 104 patients, assessed by multiple readers, improved CSI and pituitary axis delineation (Park et al., 2023). The GSU-Net model for 8-class sellar region segmentation in 213 patients achieved high accuracy (Dice 0.940) and consistency prediction (AUC 0.920) with multiple ML algorithms (Wang et al., 2021). A consistent CD cohort of 151 patients with 92.3-month follow-up utilized multiple ML models, 38 variables, and shared code for reproducibility (Zoli et al., 2020). The largest CD cohort (1045 patients) introduced a novel stacking algorithm (AUC 0.743) with an online calculator for clinical utility (Zhang et al., 2021b). Unstructured EMR data via word embedding in 419 patients enhanced IR prediction (AUC 0.793) (Zhang et al., 2021a). A deep neural network in 140 patients with 5-fold CV and dropout achieved robust validation (AUC 0.962) (Staartjes et al., 2018). A radiomics-based nomogram in 194 patients, with 2553 features and decision curve analysis (Kothari et al., 2021), yielded an AUC of 0.871 (Niu et al., 2019). The Pit-SCHEME score in 392 patients, validated by supervised ML (SML, accuracy 0.85) and pathological confirmation, strengthened reliability (McKevitt et al., 2023). A multicenter study of 729 patients achieved high CNN accuracy (0.89) for CSI, supported by external validation and Grad-CAM(Fang et al., 2024). High-resolution CUBE (0.3 mm) with DLR outperformed multi-sequence comparisons (Ishimoto et al., 2024). A U-Net DL model in 362 patients with multimodal MRI and detailed preprocessing enhanced reliability (Shu et al., 2022). A cohort of 80 patients combined ML and statistical methods with ROC thresholds (e.g., tumor volume 1.51 cm^3^) (Osorio et al., 2025). The MTMAU-Net in 926 patients delivered high segmentation (Dice 0.9084) and classification (AUC 0.89) performance with external validation (Rui et al., 2025). These strengths anchor robust advancements, guiding clinical applications with varied focuses.

#### 3.18.2 Limitations

Despite these advancements, methodological constraints limit the generalizability and robustness of findings across studies. A retrospective, single-center design with a small sample (65 patients), limited surgical confirmation (4%), and no external validation reduced DLR generalizability (Kim et al., 2021). High attrition (55.9%), a single-country setting (Australia), moderate ML performance (MCC 0.23–0.30), and lack of traditional method comparisons constrained QoL prediction (Buchlak et al., 2022). Retrospective design, observer bias in image selection, and missing surgical details limited CSI prediction generalizability (Fang et al., 2022). A small sample (86 patients), missing data, restricted classifiers, short follow-up (<24 months), and no external validation impacted DA dependency predictions (Huber et al., 2022). Retrospective, single-center data with low pathological confirmation (4.7%) and no clinical outcome evaluation restricted generalizability (Park et al., 2023). High Knosp grade/acromegaly bias (73.6–100% GH-secreting) and single-task focus limited model versatility (Wang et al., 2021). Retrospective design, missing cortisol data, heterogeneous treatments, and small ML sample (151 patients) without external validation reduced CD outcome precision (Zoli et al., 2020). Single-center design, missing values (1.7–3.9%), and retrospective nature introduced bias for IR prediction (Zhang et al., 2021b). Single-center EMR reliance, potentially biased by a single surgeon, limited generalizability (Zhang et al., 2021a). Retrospective design, moderate sample (140 patients), and cumbersome 16-variable inputs reduced GTR prediction applicability (Staartjes et al., 2018). Single-center, retrospective design, manual segmentation, and lack of omics integration limited CSI prediction scalability (Niu et al., 2019). Retrospective design, missing MRI details, and wide CIs for biochemical predictors constrained remission prediction (McKevitt et al., 2023). Retrospective design, lower Grade 3A accuracy (0.78), and imaging heterogeneity introduced bias (Fang et al., 2024). Small sample (n = 24), single-center design, and exclusion of negative MRI scans limited comparative power (Ishimoto et al., 2024). Small clinical test size (27 patients), non-transparent DL, and heterogeneous Ki67 expression hindered Ki67LI prediction (Shu et al., 2022). Missing GH/IGF-1 data and lack of Knosp grades limited biochemical remission prediction (Osorio et al., 2025). Low microinvasion sensitivity (71.43%) and single-center training data restricted CSI classification (Rui et al., 2025). These constraints signal a need for broader, prospective designs to enhance clinical reliability.

#### 3.18.3 Critical analysis

The robust methodologies of these studies, including large samples, innovative models (e.g., GSU-Net, MTMAU-Net), and rigorous validation (e.g., 5-fold CV, external validation), bolster their diagnostic reliability, particularly for CSI detection (Fang, et al. 2022, 2024; McKevitt et al., 2023; Rui et al., 2025; Staartjes et al., 2018; Wang et al., 2021; Zhang et al., 2021b). Multicenter designs and comprehensive feature sets enhance generalizability, establishing valuable benchmarks (Buchlak et al., 2022; Fang et al., 2024; McKevitt et al., 2023; Rui et al., 2025). However, retrospective designs, single-center settings, and lack of external validation introduce biases, limiting applicability (Fang et al., 2022; Huber et al., 2022; Ishimoto et al., 2024; Kim et al., 2021; McKevitt et al., 2023; Niu et al., 2019; Osorio et al., 2025; Park et al., 2023; Rui et al., 2025; Shu et al., 2022; Staartjes et al., 2018; Wang et al., 2021; Zhang, et al. 2021a, 2021b; Zoli et al., 2020). The strengths and limitations of the 17 studies evaluating ML and DL approaches for pituitary adenoma diagnostics are illustrated in Supplemental Fig. 2. Small samples and missing data further challenge ML robustness (Huber et al., 2022; Ishimoto et al., 2024; Kim et al., 2021; Shu et al., 2022; Zoli et al., 2020). The absence of traditional method comparisons and Knosp grade reporting in some studies hinders validation of ML/DL superiority (Buchlak et al., 2022; Ishimoto et al., 2024; Osorio et al., 2025; Shu et al., 2022; Zhang et al., 2021a; Zoli et al., 2020). Innovative approaches like DLR and radiomics advance diagnostics, but proprietary algorithms and imaging heterogeneity pose reproducibility challenges (Ishimoto et al., 2024; Kim et al., 2021; Park et al., 2023; Rui et al., 2025; Shu et al., 2022). These insights pave the way for prospective, externally validated studies with standardized reporting to maximize AI's clinical impact in pituitary adenoma diagnostics.

### 3.19 Evaluating clinical potential and methodological rigor in AI-driven pituitary adenoma research

#### 3.19.1 Potential clinical impact

The transformative potential of AI-driven ML and DL approaches in pituitary adenoma management shines through their ability to enhance CSI screening, diagnosis, and surgical planning, with robust findings guiding clinical applications. High-sensitivity CSI detection (96–100%) using 1-mm MRI with DLR bolsters surgical planning and radiation therapy targeting, while also improving postoperative monitoring (Kim et al., 2021). Predictive tools for postoperative QoL, though less focused on CSI, inform treatment decisions, consent processes, and recovery planning (Buchlak et al., 2022). A CNN model with accuracy up to 0.96 refines preoperative CSI diagnosis, reducing reliance on Knosp grading and optimizing surgical strategies (Fang et al., 2022). Accurate forecasting of long-term DA dependency in prolactinoma patients enhances surgical triage, minimizing DA use by identifying high-risk cases and supporting interdisciplinary decisions (Huber et al., 2022). Enhanced sellar region imaging via 1-mm DLR MRI improves CSI and pituitary axis delineation, directly aiding surgical and adjuvant therapy planning (Park et al., 2023). Automated segmentation and consistency prediction (AUC 0.920, Dice 0.940) streamline preoperative reporting, reducing manual delineation time (<1 s/image) and improving CSI-related surgical outcomes (Wang et al., 2021). Improved preoperative predictions for GTR, remission, and long-term control in CD patients, though broader than CSI, enhance patient counseling and surgical planning (Zoli et al., 2020). An online calculator for IR probability (AUC 0.743) facilitates doctor-patient communication, with CSI as a key predictor (Zhang et al., 2021b). Enhanced GTR prediction (AUC 0.962) in moderate CSI cases strengthens surgical planning and counseling (Staartjes et al., 2018). A nomogram (AUC 0.871) refines preoperative CSI prediction, offering a cost-effective tool with high sensitivity and NPV to minimize surgical risks (Niu et al., 2019). The Pit-SCHEME score (AUC-ROC 0.858) supports perioperative decisions for FPA patients, guiding surgical and follow-up strategies via CSI-relevant predictors (McKevitt et al., 2023). A CNN model (accuracy 0.89) enhances preoperative CSI diagnosis, particularly for Grade 3A cases, reducing complications (Fang et al., 2024). Superior adenoma and CSI depiction using CUBE with DLR positions it as a potential replacement for 1-mm 2D T1WI in CE-MRI protocols (Ishimoto et al., 2024). Tumor volume (<1.51 cm^3^) and IGF-1 (<718.5 ng/mL) thresholds improve surgical planning and remission counseling, with CSI refining strategies (Osorio et al., 2025). A multitask DL model (accuracy 88.05%) enhances preoperative CSI diagnosis, strengthening surgical planning (Rui et al., 2025). These advancements collectively pave the way for AI-driven tools to transform clinical practice, particularly for CSI-focused applications.

#### 3.19.2 Reproducibility

The ability to replicate study findings hinges on the availability of data and code, with varying degrees of transparency across studies. Data availability upon request, combined with PyRadiomics for partial radiomics feature replication, supports methodological reproducibility, though proprietary GSU-Net code limits full replication (Wang et al., 2021). Statistical code sharing (Supplementary Content 1) with assumed data availability upon request aids methodological replication (Zoli et al., 2020). Data availability upon reasonable request, alongside Python/scikit-learn usage, enables partial reproducibility (Zhang et al., 2021b). Raw data availability upon request, with Python and RStudio documentation, supports partial replication, though missing hardware and EMR details constrain full reproducibility (Zhang et al., 2021a). Data availability upon request, with MATLAB/LIBSVM documentation and model formulas in Supplementary S5, facilitates partial replication (Niu et al., 2019). Raw data availability and caret R package usage enhance partial reproducibility (McKevitt et al., 2023). Data availability upon request supports partial methodological replication (Fang et al., 2024). Source code availability upon reasonable request aids partial reproducibility, though restricted imaging data access limits full replication (Shu et al., 2022). Publicly available MTMAU-Net code on GitHub, with assumed data availability upon request, significantly enhances methodological replication (Rui et al., 2025). Transparent code sharing fosters robust replication where available, yet limited data access signals a need for broader sharing to strengthen cross-study validation.

#### 3.19.3 Model generalizability

The applicability of AI models across diverse settings depends on testing with varied populations, imaging protocols, and centers. Testing across five centers with different MRI systems (1.5T vs. 3.0T) achieved consistent performance (accuracy 0.89 ± 0.07), despite lacking ethnicity data, bolstering generalizability (Fang et al., 2024). Testing across two centers with different scanners (GE, Siemens) demonstrated high validation performance (Dice 83.71 ± 5.93%, accuracy 84.55%, AUC 0.87), despite CSI prevalence variations (86.4% vs. 26.7%), supporting robust generalizability (Rui et al., 2025). Multicenter external validation enhances model applicability across diverse clinical contexts, yet the absence of ethnicity data calls for inclusive population testing to broaden generalizability.

#### 3.19.4 Clinical validation

The real-world applicability of AI models depends on robust clinical validation. A clinical test on 27 patients using 1, 3, and 5 slices demonstrated translational potential, though its small size and retrospective nature limit impact (Shu et al., 2022). The scarcity of prospective validation across studies with robust data suggests that while some models show promise, broader real-world testing is essential to confirm their clinical utility and drive adoption in pituitary adenoma management.

## 4 Discussion

### 4.1 Rethinking CSI assessment in pituitary adenomas: the role of artificial intelligence

CSI is a critical determinant in the clinical management of pituitary adenomas (PAs), influencing the extent of surgical resection, operative risk, and prognosis. Accurate preoperative detection is essential, particularly in borderline or equivocal cases where imaging ambiguity can lead to over- or under-treatment. Traditional radiological evaluation, primarily via Knosp grading, is limited by interobserver variability, qualitative thresholds, and poor performance in intermediate grades (e.g., Knosp 2–3). Recent advances in AI, particularly DL, offer a compelling avenue to address these challenges. This systematic review synthesized 17 studies applying AI methods to CSI detection, segmentation, and outcome prediction in pituitary adenomas. The findings support the view that DL, when applied judiciously and with attention to data quality, may enhance diagnostic accuracy, reduce variability, and improve clinical decision-making.

To contextualize this emerging role of AI, it's helpful to recognize its current categorization across neuro-oncology and diagnostic radiology. AI applications are commonly divided into: (1) image enhancement and reconstruction, (2) segmentation and volume quantification, (3) classification and diagnostic support, and (4) predictive modeling for outcomes. The 17 studies in this review collectively contribute across all four domains, with several integrating dual or multi-task architectures to unify tasks. This mirrors current trends seen in broader neuroradiology literature, where DL systems increasingly aim for end-to-end pipelines.

Nevertheless, enthusiasm for DL must be tempered by a realistic appraisal of its limitations. While AI models outperformed conventional techniques in many scenarios, particularly in imaging resolution, segmentation precision, and classification accuracy, these findings derive largely from retrospective, single-center studies with limited external validation. Thus, while the potential is substantial, further work is needed to translate these findings into robust clinical practice.

### 4.2 Enhancing image quality: deep learning-based reconstruction

Among the most immediate benefits of DL in neuroimaging is improved image fidelity through reconstruction algorithms. DLR was applied to contrast-enhanced MRI sequences in several studies, yielding significant improvements in SNR and CNR. For example, one study demonstrated that 1 mm DLR MRI achieved an SNR of 308.9 and tumor-to-brain CNR of 52.8, compared to SNRs of 253.9 and lower CNRs on 3 mm conventional imaging.

These improvements were not merely theoretical. Interobserver agreement improved significantly with DLR (Kappa 0.75 vs. 0.41 on conventional MRI), and lesion conspicuity, particularly along the medial wall of the cavernous sinus, was rated higher by experienced neuroradiologists. This is of direct clinical relevance, as false negatives or false positives in these regions can impact surgical exposure and risk.

Recent theories in AI-based imaging suggest that these models do more than denoise; they enhance perceptual saliency by amplifying lesion-specific textures, an effect likely contributing to the improved clinical interpretability observed in CSI detection. Yet, image acquisition parameters varied widely between studies, including differences in slice thickness (0.3–5 mm), sequence type (SPGR, CUBE, 2D/3D T1), and post-contrast timing. These discrepancies could affect reproducibility, especially in real-world institutions with variable scanner models or protocols. Thus, multicenter harmonization of imaging standards remains a priority for DL's clinical translation.

### 4.3 Tumor segmentation: from labor-intensive to automated

Accurate tumor segmentation is foundational for surgical navigation, radiation therapy, and tumor monitoring. Manual segmentation is resource-intensive, time-consuming, and prone to interobserver variability. DL-based models, particularly U-Net variants such as nnU-Net, UNETR, and MTMAU-Net, have now achieved state-of-the-art performance, with Dice similarity coefficients exceeding 90% in several studies.

Multi-task models like MTMAU-Net further integrate CSI classification and segmentation within a single architecture, allowing simultaneous delineation and staging of PAs. Such dual-output systems not only enhance workflow efficiency but also improve consistency by learning both morphological and semantic features. Importantly, inference times were typically under 1 s per image, making these models suitable for real-time applications once validated.

Still, as noted in implementation science literature, task unification can introduce interpretability trade-offs. Few studies examined attention maps or feature attribution to ensure that segmentation and classification heads relied on consistent regions. While internal validation metrics were high, generalizability remains a concern. Only two studies conducted external validation across institutions. Without broader multicenter testing, the risk of domain shift—where performance degrades on data outside the training distribution—remains real. External benchmarking datasets should be prioritized.

### 4.4 CSI classification: DL outperforms traditional methods—but not without limits

The most striking finding across included studies was the superior performance of DL models in classifying CSI. CNNs using ResNet, EfficientNet, and DenseNet backbones consistently outperformed logistic regression and Knosp grading, with AUCs as high as 0.98 in ResNet50-based models. Particularly in Knosp Grade 2–3 cases, where human agreement is low, DL demonstrated enhanced sensitivity and specificity.

However, claims that DL “outperforms in nearly every dimension” should be qualified. First, only three studies validated models externally. Second, even high AUC values may not translate to clinically actionable predictions without calibration or probability thresholds. Third, none of the studies included prospective deployment in clinical environments, and thus, real-world efficacy and clinician trust remain unproven.

Moreover, comparisons were largely limited to Knosp grading and logistic regression. Very few studies compared DL to classical radiomics approaches, which use handcrafted features such as texture, shape, and intensity statistics extracted from imaging data. Radiomics models have demonstrated robust performance in other CNS tumors, including gliomas and meningiomas. One included study found that radiomics-based nomograms slightly outperformed clinico-radiological models (AUC 0.871 vs. 0.828; p = 0.035), suggesting their continued relevance.

From a theoretical standpoint, DL classifiers may derive performance from their capacity to abstract volumetric and spatial patterns beyond human perception. However, this abstraction comes at the cost of interpretability and consistency, particularly across institutions with divergent labeling schemes (e.g., Knosp ≥1 vs. ≥3). A hybrid approach combining radiomics interpretability with DL feature extraction may offer the best of both worlds. Few studies in the CSI domain have explored this integrative strategy, presenting a promising direction for future research.

To quantify the diagnostic performance of these DL models and provide a comprehensive evaluation across studies, a meta-analysis of predictive metrics was conducted.

The combined results from multiple studies show that AI models perform strongly in detecting cavernous sinus invasion (CSI). The average AUC-ROC, a measure of diagnostic accuracy, was 0.93, indicating excellent performance. Deep learning (DL) models achieved a higher AUC-ROC of 0.95 compared to machine learning (ML) models at 0.90 (p = 0.02). The best result came from a 1-mm MRI with deep learning reconstruction (DLR) (Kim et al., 2021), which reached an AUC-ROC of 0.98, thanks to clearer images (SNR 132.9, CNR 52.8). The top ML model, a Random Forest using the Pit-SCHEME score (McKevitt et al., 2023), scored an AUC-ROC of 0.97 but had lower sensitivity (0.78), meaning it missed some positive cases compared to DL models, which ranged from 0.77 to 1.00. Knosp grading (Staartjes et al., 2018) was less reliable, with a specificity of 0.70, especially for tricky cases. A statistical model showed an average sensitivity of 0.87 and specificity of 0.88, with the SROC curve (Fig. 11) showing how these balance out. There was moderate variation across studies (I^2^ = 58%), mainly because CSI rates differed widely (25.5% to 67.9%), as confirmed by meta-regression (β = 0.023, 95% CI [0.008 to 0.038], p = 0.004). This reflects diverse patient groups. Tests confirmed these results are reliable. Quality checks using QUADAS-2 showed moderate to low bias risk, with multicenter studies (Fang et al., 2024; McKevitt et al., 2023; Niu et al., 2019) being more trustworthy due to better generalizability. There was little evidence of publication bias (Egger's p = 0.18, trim-and-fill: no missing studies). Future studies should focus on real-world testing, standard imaging methods, and practical use to make these models more impactful for CSI detection.

### 4.5 Beyond diagnosis: outcome prediction and decision support

Several included studies extended the role of AI beyond diagnosis, training DL and ML models to predict surgical outcomes, endocrinological remission, and dopamine agonist resistance. A CNN model predicting GTR achieved an AUC of 0.962, outperforming logistic regression (AUC 0.868, p < 0.001). These tools demonstrate potential for patient stratification and preoperative counseling.

However, success depended heavily on data structure. Structured imaging and clinical variables led to high predictive performance (AUCs 0.87–1.00), while models trained on free-text EMRs underperformed significantly (AUC ∼0.46). These disparities emphasize the need for curated, standardized input data. Furthermore, despite technical success, no studies evaluated clinical utility via decision curve analysis or real-time clinical deployment.

From a systems design perspective, future predictive tools must address the complex interaction between imaging phenotype, tumor biology, and treatment modality. Current models largely rely on imaging alone, while integrated multimodal models incorporating hormonal markers, genomics, and intraoperative findings remain rare. This gap offers a clear trajectory for development.

### 4.6 Ethical, regulatory, and implementation science perspectives

Despite high technical accuracy, ethical and translational dimensions were underrepresented. None of the included studies reported sex-stratified performance, despite the known female predominance in prolactinomas and mixed adenomas. The absence of subgroup analysis raises concerns about performance drift and demographic bias.

Likewise, explainability techniques like Grad-CAM or attention heatmaps were rarely employed, hindering clinician trust and regulatory compliance. According to recent FDA and EMA guidelines for software-as-a-medical-device (SaMD), model transparency and post-deployment monitoring are essential. Yet, these components were not addressed.

Furthermore, there was limited discussion of cost, integration into radiology workflows, or impact on multidisciplinary team (MDT) decision-making. While many studies envisioned AI as augmentative to clinicians, none conducted formal usability testing or workflow simulation. For AI to be integrated successfully, real-world readiness, including training time, PACS integration, and medico-legal responsibility, must be considered.

### 4.7 Meta-analysis, labeling consistency, and heterogeneity

Pooled AUC-ROC values from selected studies indicated excellent diagnostic performance for CSI detection in pituitary adenomas (DL: 0.95, 95% CI: 0.93–0.97; ML: 0.90, 95% CI: 0.86–0.93), with an overall pooled AUC-ROC of 0.93 (95% CI: 0.91–0.95). However, moderate heterogeneity (I^2^ = 58%, τ^2^ = 0.01, p = 0.02) was observed, primarily driven by varying prevalence rates (25.5% to 67.9%) across studies, as confirmed by meta-regression (β = 0.023, 95% CI: 0.008–0.038, p = 0.004). Labeling standards for CSI were inconsistent: some studies used Knosp ≥1, others Knosp ≥3, while multicenter studies (Fang et al., 2024; McKevitt et al., 2023; Niu et al., 2019)prioritized surgical confirmation as the gold standard, and single-center studies (Kim et al., 2021; Staartjes et al., 2018; Wang et al., 2021) often relied on intraoperative or pathological confirmation. This variability introduces training noise, potentially lowering the accuracy ceiling for DL models.

To better understand these methodological challenges, it is important to examine the assumptions made in calculating study metrics, as they impact the reliability of results.

To better understand the challenges in these studies, it's important to look at the assumptions made when calculating results, as these can affect how reliable and comparable the findings are. For example, one study (Wang et al., 2021)assumed that tumor consistency in the test group was split roughly 35% firm and 65% soft tumors. It also assumed the test data for Knosp grade prediction covered a full range of grades (0 to 4), with models like KNN and AdaBoost achieving good accuracy based on confusion matrices. Another study (Fang et al., 2024)estimated true positive, false negative, true negative, and false positive values using reported sensitivity, specificity, and an assumed CSI prevalence of 25.5%, with about 21 positive and 61 negative cases in the test set. In a different study (Ishimoto et al., 2024), contrast-to-noise ratio (CNR) values were assumed to reflect the contrast between pituitary adenoma and reference tissues (like brain parenchyma or normal pituitary gland) compared to background noise. It was assumed that CUBE with DLR gave better contrast than non-DLR CUBE or SPGR and was similar to 1-mm 2D T1WI with DLR, improving visibility of adenomas and nearby structures. Similarly, another study (Rui et al., 2025)estimated true positive, false negative, true negative, and false positive values based on CSI prevalence (26.7% for training, 86.4% for validation) and metrics like accuracy, sensitivity, and specificity. Knosp grading results were aligned with MTMAU-Net's accuracy, keeping calculations consistent despite some rounded numbers. These assumptions, while needed to combine data, can add variability, especially when CSI rates or tumor traits differ across studies. Clear reporting and standard methods are needed to make results more comparable.

Few studies addressed these labeling discrepancies, and inter-rater reliability among human labelers was rarely reported. Given that DL models depend on the quality of their annotations, standardized CSI ground truths, ideally based on surgical confirmation, are critical. This mirrors trends in other fields (e.g., BraTS for gliomas), where centralized, multi-expert labeling has improved benchmarking.

Additionally, subgroup analyses by Knosp grade, tumor subtype, or imaging modality were limited. Only the 1-mm MRI DLR model (Kim et al., 2021) demonstrated superior performance (AUC-ROC: 0.95–0.98) compared to Knosp-based models (AUC-ROC: 0.868). Reporting calibration curves, decision thresholds, and performance across clinically relevant strata (e.g., prevalence or imaging conditions) would enhance interpretability and clinical trust.

### 4.8 Clinical implications and future directions

The reviewed studies clearly demonstrate the technical viability of DL for CSI detection and PA management. However, clinical adoption will require:

1. **Prospective, multicenter trials** with standardized imaging protocols and diverse populations.
2. **Fairness and explainability audits** to address demographic bias and build clinician trust.
3. **Hybrid models** combining handcrafted features with DL abstraction to improve interpretability.
4. **Integration into MDT workflows**, PACS environments, and navigation systems with tested usability.
5. **Regulatory alignment** with existing frameworks for AI devices.

The landscape of pituitary AI is rapidly evolving toward multi-dimensional data integration. While our systematic review focuses on CSI prediction using static or radiomic features, emerging evidence from 2025, such as the work by (Motamed et al., 2025) highlights the potential of Hybrid Convolutional and Recurrent Neural Networks (CNN-LSTM). By leveraging Dynamic Contrast-Enhanced MRI (DCE-MRI), these models can capture not only spatial morphology (via CNN) but also the temporal kinetics of contrast uptake (via LSTM). This dual-path approach offers a processing speed of 0.17 s and superior robustness against scanner-specific noise. From a clinical perspective, the synergy of spatial and temporal features is particularly relevant for CSI assessment. The medial wall of the cavernous sinus is often difficult to delineate on static T1-weighted images; however, tracking the differential enhancement between the tumor and the internal carotid artery over time as demonstrated in the CNN-LSTM framework could provide the high-resolution temporal fingerprint needed to identify subtle invasions. Although current hybrid models primarily focus on size-based classification, adapting these architectures for CSI detection represents a critical frontier. Future research should prioritize Temporal Radiomics to bridge the gap between automated classification and precision surgical planning.

Moreover, AI's role should be framed as augmentative rather than replacement. In CSI detection, DL can reduce interobserver variability, particularly in intermediate Knosp grades. In segmentation, DL can relieve manual workload. In outcome prediction, AI may guide personalized counseling.Nevertheless, this transition demands not only technical refinement but cultural change, ethical vigilance, and health system adaptability.

## 5 Conclusion

This enriched discussion demonstrates that while DL has outperformed conventional methods in CSI assessment across multiple technical metrics, its clinical value remains contingent on validation, fairness, explainability, and usability. The reviewed 17 studies illustrate foundational advances but also underscore the critical next steps required for translational impact. Future research should prioritize prospective validation, standardized labeling, ethical deployment, and robust implementation science.

With rigorous development and stakeholder alignment, AI has the potential not only to enhance current diagnostic workflows but to redefine standards in the multidisciplinary care of pituitary adenomas.

## Declaration of generative AI and AI-assisted technologies in the manuscript preparation process

During the preparation of this work the authors used ChatGPT in order to check grammar. After using this tool, the authors reviewed and edited the content as needed and take full responsibility for the content of the published article.

## Funding

This research did not receive any specific grant from funding agencies in the public, commercial, or not-for-profit sectors.

## CRediT authorship contribution statement

**Farzan Asadirad:** Investigation, Validation, Visualization, Writing – original draft. **Mohammad Rezaei:** Investigation, Visualization, Writing – review & editing. **Parna Ghannadikhosh:** Investigation, Writing – review & editing. **Hadi Salehpour:** Investigation, Writing – review & editing. **Alireza Motamedi:** Investigation, Writing – review & editing. **Mobin Mobadersani:** Investigation, Supervision, Writing – review & editing. **Niloofar soleimannezhad:** Methodology, Validation, Writing – review & editing. **Sevil Ghaffarzadeh Rad:** Investigation, Validation, Writing – review & editing. **Esmaeil Gharepapagh:** Investigation, Validation, Writing – review & editing. **Sahar Rezaei:** Investigation, Methodology, Project administration, Supervision, Validation, Writing – review & editing. **Mahsa Karbasi:** Investigation, Validation, Writing – review & editing. **Hossein Arabi:** Validation, Writing – review & editing.

## Declaration of competing interest

The authors declare that they have no known competing financial interests or personal relationships that could have appeared to influence the work reported in this paper.

## Data Availability

No data was used for the research described in the article.

## Abbrivations

Artificial Intelligence: AI, Machine Learning: ML, Deep Learning: DL, Convolutional Neural Network: CNN, Support Vector Machine: SVM, Random Forest: RF, Logistic Regression: LR, K-Nearest Neighbors: KNN, Supervised Machine Learning: SML, Synthetic Minority Oversampling Technique: SMOTE, Least Absolute Shrinkage and Selection Operator: LASSO, Magnetic Resonance Imaging: MRI, T2-Weighted Imaging: T2WI, Contrast-Enhanced T1-Weighted: CE-T1, Spoiled Gradient Recalled: SPGR, Cavernous Sinus Invasion: CSI, Intraoperative Magnetic Resonance Imaging: IOMRI, Field of View: FOV, Region of Interest: ROI, Signal-to-Noise Ratio: SNR, Contrast-to-Noise Ratio: CNR, Area Under the Curve: AUC, Area Under the Receiver Operating Characteristic Curve: AUC-ROC, Matthews Correlation Coefficient: MCC, Negative Predictive Value: NPV, Cross-Validation: CV, Gradient-weighted Class Activation Mapping: Grad-CAM, Shapley Additive Explanations: SHAP, Decision Curve Analysis: DCA, Gross-Total Resection: GTR, Immediate Remission: IR, Adrenocorticotropic Hormone: ACTH, Growth Hormone: GH, Prolactin: PRL, Thyroid-Releasing Hormone: TRH, Thyroid-Stimulating Hormone: TSH, Insulin-Like Growth Factor 1: IGF-1, Ki67 Labeling Index: Ki67LI, Pituitary Adenoma: PA, Functional Pituitary Adenoma: FPA, Natural Language Processing: NLP, Internal Carotid Artery: ICA, Optic Chiasm: OC, Dopamine Agonist: DA, Electronic Medical Record: EMR, Deep Learning Reconstruction: DLR, Quality of Life: QoL, Risk Of Bias In Non-randomised Studies of Interventions: ROBINS-I, Institutional Review Board: IRB, Interquartile Range: IQR, Standard Deviation: SD, Yangpu Hospital: YFPH, Second Affiliated Hospital: SAH, Tongji Hospital: TPH.
