# Supplementary material for: AI-Powered MRI Radiomics and Deep Learning for Preoperative Prediction of Cavernous Sinus Invasion in Pituitary Adenomas: A Clinically Oriented Review of Current Evidence
Source: Neuroimage Rep. 2026 Jun 2;6(2):100354. doi: 10.1016/j.ynirp.2026.100354 (PMC13282517; doi:10.1016/j.ynirp.2026.100354)
Supplement: Multimedia component 1 [file mmc1.docx]

**Supplementary Materials**

**Supplementary Table 1.** Systematic Search and Selection of Relevant Articles for Review.

| Dataset | Search Strategy | Number of articles |
| --- | --- | --- |
| PubMed | ((((((((((((((((((((((((((((((((((((((((((Artificial Intelligence[MeSH Terms]) OR (machine learning[MeSH Terms])) OR (machine learning[Other Term])) OR (Artificial Intelligence[Other Term])) OR (Transfer Learning[Other Term])) OR (Computer Reasoning[Other Term])) OR (AI[Other Term])) OR (Artificial Intelligence[Other Term])) OR (Machine Intelligence[Other Term])) OR (Computational Intelligence[Other Term])) OR (Knowledge Acquisition[Other Term])) OR (Knowledge Representation[Other Term])) OR (deep learning[Other Term])) OR (Computer Vision System*[Other Term])) OR (Computing Methodology[Other Term])) OR (High Performance Computing[Other Term])) OR (computer neural network[Other Term])) OR (machine learning[Other Term])) OR (intelligent retrieval[Other Term])) OR (machine gunning[Other Term])) OR (ambient intelligence[Other Term])) OR (ambient intelligence[Title/Abstract])) OR (machine gunning[Title/Abstract])) OR (intelligent retrieval[Title/Abstract])) OR (machine learning[Title/Abstract])) OR (computer neural network[Title/Abstract])) OR (High Performance Computing[Title/Abstract])) OR (Computing Methodology[Title/Abstract])) OR (Computer Vision System*[Title/Abstract])) OR (deep learning[Title/Abstract])) OR (Knowledge Representation[Title/Abstract])) OR (Knowledge Acquisition[Title/Abstract])) OR (Computational Intelligence[Title/Abstract])) OR (Machine Intelligence[Title/Abstract])) OR (Artificial Intelligence[Title/Abstract])) OR (AI[Title/Abstract])) OR (Computer Reasoning[Title/Abstract])) OR (Transfer Learning[Title/Abstract])) OR (machine learning[Title/Abstract])) OR (Artificial Intelligence[Title/Abstract])) AND (((((Cavernous Sinus[MeSH Terms]) OR (Cavernous Sinus*[Other Term])) OR (sinus cavernous[Other Term])) OR (sinus cavernous[Title/Abstract])) OR (Cavernous Sinus*[Title/Abstract]))) AND (((((((((((((Pituitary Gland[MeSH Terms]) OR (Pituitary Gland[Other Term])) OR (Infundibular Stalk[Other Term])) OR (Hypophysis[Other Term])) OR (Infundibulum[Other Term])) OR (Hypophyseal Stalk[Other Term])) OR (pituitary[Other Term])) OR (pituitary[Title/Abstract])) OR (Hypophyseal Stalk[Title/Abstract])) OR (Infundibulum[Title/Abstract])) OR (Hypophysis[Title/Abstract])) OR (Infundibular Stalk[Title/Abstract])) OR (Pituitary Gland[Title/Abstract]))) AND ((((((((((((((((((((adenoma[MeSH Terms])) OR (adenoma*[Other Term])) OR (Neoplasm*[Other Term])) OR (benign tumor[Other Term])) OR (benign tumour[Other Term])) OR (non cancerous tumor[Other Term])) OR (non cancerous tumour[Other Term])) OR (nonmalignant neoplasm[Other Term])) OR (nonmalignant tumor[Other Term])) OR (nonmalignant tumour[Other Term])) OR (non cancerous tumour[Title/Abstract])) OR (benign tumour[Title/Abstract])) OR (nonmalignant tumour[Title/Abstract])) OR (nonmalignant tumor[Title/Abstract])) OR (nonmalignant neoplasm[Title/Abstract])) OR (non cancerous tumor[Title/Abstract])) OR (benign tumor[Title/Abstract])) OR (Neoplasm*[Title/Abstract])) OR (Adenoma*[Title/Abstract])) | 12 |
| Scopus | (TITLE-ABS-KEY("Artificial Intelligence" OR "machine learning" OR "Transfer Learning" OR "Computer Reasoning" OR "AI" OR "Artificial Intelligence" OR "Machine Intelligence" OR "Computational Intelligence" OR "Knowledge Acquisition" OR "Knowledge Representation" OR "deep learning" OR "Computer Vision System*" OR "Computing Methodology" OR "High Performance Computing" OR "computer neural network" OR "machine learning" OR "intelligent retrieval" OR "machine gunning" OR "ambient intelligence") AND TITLE-ABS-KEY("Cavernous Sinus*" OR "sinus cavernous") AND TITLE-ABS-KEY("Pituitary Gland" OR "Infundibular Stalk" OR "Hypophysis" OR "Infundibulum" OR "Hypophyseal Stalk" OR "pituitary") AND TITLE-ABS-KEY("Adenoma*" OR "Neoplasm*" OR "benign tumor" OR "non cancerous tumor" OR "nonmalignant neoplasm" OR "nonmalignant tumor" OR "nonmalignant tumour" OR "benign tumour" OR "non cancerous tumour")) | 20 |
| Web Of Science | (((((((((((((((((((TS=("Artificial Intelligence")) OR TS=("machine learning")) OR TS=("Transfer Learning")) OR TS=("Computer Reasoning")) OR TS=("AI")) OR TS=("Artificial Intelligence")) OR TS=("Machine Intelligence")) OR TS=("Computational Intelligence")) OR TS=("Knowledge Acquisition")) OR TS=("Knowledge Representation")) OR TS=("deep learning")) OR TS=("Computer Vision System*")) OR TS=("Computing Methodology")) OR TS=("High Performance Computing")) OR TS=(computer neural network)) OR TS=("machine learning")) OR TS=("intelligent retrieval")) OR TS=("machine gunning")) OR TS=("ambient intelligence")) AND ((TS=("Cavernous Sinus*")) OR TS=("sinus cavernous")) AND ((((((TS=("Pituitary Gland")) OR TS=("Infundibular Stalk")) OR TS=("Hypophysis")) OR TS=("Infundibulum")) OR TS=("Hypophyseal Stalk")) OR TS=("pituitary ")) AND (((((((((TS=("Adenoma*")) OR TS=("Neoplasm*")) OR TS=("benign tumor")) OR TS=("non cancerous tumor")) OR TS=("nonmalignant neoplasm")) OR TS=("nonmalignant tumor")) OR TS=("nonmalignant tumour")) OR TS=("benign tumour")) OR TS=("non cancerous tumour")) | 26 |
| Embase | '('Artificial Intelligence' OR 'machine learning' OR 'Transfer Learning' OR 'Computer Reasoning' OR 'AI' OR 'Artificial Intelligence' OR 'Machine Intelligence' OR 'Computational Intelligence' OR 'Knowledge Acquisition' OR 'Knowledge Representation' OR 'deep learning' OR 'Computer Vision System*' OR 'Computing Methodology' OR 'High Performance Computing' OR 'computer neural network' OR 'machine learning' OR 'intelligent retrieval' OR 'machine gunning' OR 'ambient intelligence'):ab,kw,ti AND ('Cavernous Sinus*' OR 'sinus cavernous'):ab,kw,ti AND ('Pituitary Gland' OR 'Infundibular Stalk' OR 'Hypophysis' OR 'Infundibulum' OR 'Hypophyseal Stalk' OR 'pituitary'):ab,kw,ti AND ('adenoma*' OR 'Neoplasm*' OR 'benign tumor' OR 'non cancerous tumor' OR 'nonmalignant neoplasm' OR 'nonmalignant tumor' OR 'nonmalignant tumour' OR 'benign tumour' OR 'non cancerous tumour'):ab,kw,ti | 20 |

**Supplementary Table 2.** Comprehensive performance metrics across studies.

| Study | Model/Condition | TP | FN | TN | FP | Total Sample Size (N) | AUC-ROC (95% CI) | Sensitivity (%) | Specificity (%) | PPV (%) | NPV (%) | F1-Score | Accuracy (%) | SNR | CNR | Other Metrics |
| --- | --- | --- | --- | --- | --- | --- | --- | --- | --- | --- | --- | --- | --- | --- | --- | --- |
| (Kim et al., 2021) | 3-mm MRI (Reader 1, Residual Tumor) | 34 | 11 | 19 | 1 | 65 | 0.85 (0.77–0.93) | 76 | 95 | 97 (85–100) | 63 (44–80) | 85.14 | 81.54 | - | - | Kappa=0.63 |
|  | 1-mm MRI (Reader 1, Residual Tumor) | 40 | 5 | 15 | 5 | 65 | 0.82 (0.71–0.93) | 89 | 75 | 89 (76–96) | 75 (51–91) | 89.00 | 84.62 | 57.1 | 22.5 | Kappa=0.58, CNR (vs. normal pituitary)=15.7 |
|  | 1-mm MRI DLR (Reader 1, Residual Tumor) | 42 | 3 | 18 | 2 | 65 | 0.92 (0.84–0.99) | 93 | 90 | 95 (85–99) | 86 (64–97) | 93.99 | 92.31 | 132.9 | 52.8 | Kappa=0.62, Misdiagnosed: 13/65, CNR (vs. normal pituitary)=38.4 |
|  | 3-mm MRI (Reader 2, Residual Tumor) | 37 | 8 | 19 | 1 | 65 | 0.89 (0.81–0.96) | 82 | 95 | 97 (86–100) | 70 (50–86) | 88.87 | 86.15 | - | - | Kappa=0.63 |
|  | 1-mm MRI (Reader 2, Residual Tumor) | 33 | 12 | 16 | 4 | 65 | 0.77 (0.66–0.88) | 73 | 80 | 89 (75–97) | 57 (37–76) | 80.22 | 75.38 | 57.1 | 22.5 | Kappa=0.58, CNR (vs. normal pituitary)=15.7 |
|  | 1-mm MRI DLR (Reader 2, Residual Tumor) | 40 | 5 | 18 | 2 | 65 | 0.89 (0.81–0.98) | 89 | 90 | 95 (84–99) | 78 (56–93) | 91.90 | 89.23 | 132.9 | 52.8 | Kappa=0.62, Misdiagnosed: 7/65, CNR (vs. normal pituitary)=38.4 |
|  | 3-mm MRI (Reader 1, CSI) | 17 | 6 | 39 | 3 | 65 | 0.83 (0.73–0.93) | 74 | 93 | 85 (62–97) | 87 (73–95) | 79.12 | 86.15 | - | - | Kappa=0.68 |
|  | 1-mm MRI (Reader 1, CSI) | 18 | 5 | 37 | 5 | 65 | 0.83 (0.73–0.93) | 78 | 88 | 78 (56–93) | 88 (74–96) | 78.00 | 84.62 | 57.1 | 22.5 | Kappa=0.52, CNR (vs. normal pituitary)=15.7 |
|  | 1-mm MRI DLR (Reader 1, CSI) | 22 | 1 | 40 | 2 | 65 | 0.95 (0.90–1.00) | 96 | 95 | 92 (73–99) | 98 (87–100) | 93.94 | 95.38 | 132.9 | 52.8 | Kappa=0.72, Misdiagnosed: 7/65, CNR (vs. normal pituitary)=38.4 |
|  | 3-mm MRI (Reader 2, CSI) | 19 | 4 | 38 | 4 | 65 | 0.87 (0.77–0.96) | 83 | 90 | 83 (61–95) | 90 (77–97) | 83.00 | 87.69 | - | - | Kappa=0.68 |
|  | 1-mm MRI (Reader 2, CSI) | 17 | 6 | 38 | 4 | 65 | 0.82 (0.72–0.92) | 74 | 90 | 81 (58–95) | 86 (73–95) | 77.34 | 84.62 | 57.1 | 22.5 | Kappa=0.52, CNR (vs. normal pituitary)=15.7 |
|  | 1-mm MRI DLR (Reader 2, CSI) | 23 | 0 | 40 | 2 | 65 | 0.98 (0.94–1.00) | 100 | 95 | 92 (74–99) | 100 (91–100) | 95.83 | 96.92 | 132.9 | 52.8 | Kappa=0.72, Misdiagnosed: 7/65, CNR (vs. normal pituitary)=38.4 |
| (Buchlak et al., 2022) | AdaBoost (LR, SMOTE) |  |  |  |  |  | 0.70 | 84 | 44 | 76 | 86 (71–94) | 0.79 | 71 | - | - | MCC=0.30 |
|  | AdaBoost (LR, no SMOTE) |  |  |  |  |  | 0.70 | 80 | 48 | 88 | - | 0.84 | 75 | - | - | MCC=0.26 |
|  | Logistic Regression (LR, SMOTE) |  |  |  |  |  | 0.68 | 83 | 40 | 70 | 85 (70–93) | 0.76 | 67 | - | - | MCC=0.25 |
|  | Neural Network (RFE, no SMOTE) |  |  |  |  |  | 0.62 | 78 | 54 | 93 | 88 (74–95) | 0.85 | 75 | - | - | MCC=0.24 |
|  | Neural Network (LR, no SMOTE) |  |  |  |  |  | 0.65 | 81 | 43 | 81 | - | 0.81 | 71 | - | - | MCC=0.23 |
|  | Random Forest (RFE, no SMOTE) |  |  |  |  |  | 0.66 | 77 | 57 | 95 | - | 0.85 | 75 | - | - | MCC=0.22 |
|  | Neural Network (LR, SMOTE) |  |  |  |  |  | 0.64 | 81 | 39 | 73 | - | 0.76 | 67 | - | - | MCC=0.21 |
|  | Random Forest (LR, SMOTE) |  |  |  |  |  | 0.65 | 80 | 38 | 74 | - | 0.77 | 67 | - | - | MCC=0.20 |
|  | GBM (RFE, no SMOTE) |  |  |  |  |  | 0.65 | 77 | 47 | 90 | - | 0.83 | 73 | - | - | MCC=0.18 |
|  | SVM (LR, SMOTE) |  |  |  |  |  | 0.65 | 79 | 39 | 80 | - | 0.80 | 69 | - | - | MCC=0.18 |
|  | GBM (LR, SMOTE) |  |  |  |  |  | 0.63 | 80 | 36 | 70 | - | 0.75 | 65 | - | - | MCC=0.18 |
|  | KNN (LR, no SMOTE) |  |  |  |  |  | 0.62 | 77 | 55 | 93 | - | 0.84 | 74 | - | - | MCC=0.17 |
|  | Logistic Regression (LR, no SMOTE) |  |  |  |  |  | 0.71 | 77 | 0 | 97 | - | 0.86 | 76 | - | - | MCC=0.16 |
|  | KNN (RFE, SMOTE) |  |  |  |  |  | 0.57 | 78 | 42 | 86 | - | 0.82 | 71 | - | - | MCC=0.16 |
|  | KNN (LR, SMOTE) |  |  |  |  |  | 0.63 | 81 | 32 | 57 | - | 0.67 | 58 | - | - | MCC=0.15 |
|  | Gaussian NB (LR, SMOTE) |  |  |  |  |  | 0.65 | 81 | 31 | 49 | - | 0.61 | 54 | - | - | MCC=0.14 |
|  | Random Forest (LR, no SMOTE) |  |  |  |  |  | 0.63 | 78 | 34 | 89 | - | 0.83 | 73 | - | - | MCC=0.14 |
|  | AdaBoost (RFE, no SMOTE) |  |  |  |  |  | 0.64 | 77 | 40 | 89 | - | 0.82 | 72 | - | - | MCC=0.13 |
|  | Gaussian NB (LR, no SMOTE) |  |  |  |  |  | 0.64 | 88 | 28 | 19 | - | 0.31 | 38 | - | - | MCC=0.13 |
|  | Logistic Regression (RFE, no SMOTE) |  |  |  |  |  | 0.65 | 76 | 0 | 97 | - | 0.85 | 75 | - | - | MCC=0.11 |
|  | Logistic Regression (RFE, SMOTE) |  |  |  |  |  | 0.59 | 79 | 29 | 49 | - | 0.60 | 52 | - | - | MCC=0.10 |
|  | AdaBoost (RFE, SMOTE) |  |  |  |  |  | 0.61 | 79 | 29 | 48 | - | 0.59 | 52 | - | - | MCC=0.09 |
|  | KNN (RFE, no SMOTE) |  |  |  |  |  | 0.57 | 76 | 40 | 96 | - | 0.85 | 74 | - | - | MCC=0.09 |
|  | Neural Network (RFE, SMOTE) |  |  |  |  |  | 0.60 | 77 | 31 | 51 | - | 0.60 | 53 | - | - | MCC=0.08 |
|  | GBM (LR, no SMOTE) |  |  |  |  |  | 0.66 | 76 | 31 | 85 | - | 0.80 | 69 | - | - | MCC=0.06 |
|  | Gaussian NB (RFE, no SMOTE) |  |  |  |  |  | 0.65 | 0 | 25 | 45 | - | 0.00 | 50 | - | - | MCC=0.06 |
|  | SVM (RFE, no SMOTE) |  |  |  |  |  | 0.65 | 75 | 0 | 92 | - | 0.83 | 71 | - | - | MCC=0.05 |
|  | Random Forest (RFE, SMOTE) |  |  |  |  |  | 0.59 | 77 | 28 | 51 | - | 0.60 | 52 | - | - | MCC=0.05 |
|  | Gaussian NB (RFE, SMOTE) |  |  |  |  |  | 0.58 | 82 | 22 | 36 | - | 0.42 | 45 | - | - | MCC=0.05 |
|  | GBM (RFE, SMOTE) |  |  |  |  |  | 0.57 | 74 | 28 | 49 | - | 0.58 | 50 | - | - | MCC=0.02 |
|  | SVM (LR, no SMOTE) |  |  |  |  |  | 0.68 | 74 | 0 | 97 | - | 0.84 | 73 | - | - | MCC=-0.01 |
|  | SVM (RFE, SMOTE) |  |  |  |  |  | 0.50 | 74 | 25 | 51 | - | 0.59 | 50 | - | - | MCC=-0.02 |
| (Fang et al., 2022) | CNN (Fold 1) | 17 | 4 | 43 | 11 | 75 | 0.89 | 61 | 91 | 61 | 91.49 | 0.69 | 80 | - | - | DOR=16.61 |
|  | CNN (Fold 2) | 19 | 2 | 53 | 1 | 75 | 0.98 | 95 | 96 | 95 | 96.36 | 0.93 | 96 | - | - | DOR=503.50 |
|  | CNN (Fold 3) | 16 | 4 | 47 | 7 | 74 | 0.92 | 70 | 92 | 70 | 92.16 | 0.74 | 85 | - | - | DOR=26.86 |
|  | CNN (Fold 4) | 17 | 3 | 51 | 3 | 74 | 0.94 | 85 | 94 | 85 | 94.44 | 0.85 | 92 | - | - | DOR=96.33 |
|  | CNN (Fold 5) | 16 | 4 | 47 | 6 | 73 | 0.93 | 73 | 92 | 73 | 92.16 | 0.76 | 86 | - | - | DOR=31.33 |
|  | Knosp (0–1/2–4) | 98 | 4 | 123 | 146 | 371 | 0.84 | 96 | 46 | 40 | 96.85 | 0.57 | 60 | - | - | DOR=20.59 |
|  | Knosp (0–2/3a–4) | 83 | 19 | 190 | 79 | 371 | 0.84 | 81 | 71 | 51 | 90.91 | 0.63 | 74 | - | - | DOR=10.51 |
|  | Knosp (0–3a/3b–4) | 44 | 58 | 255 | 14 | 371 | 0.84 | 43 | 95 | 76 | 81.47 | 0.55 | 81 | - | - | DOR=13.81 |
| (Huber et al., 2022) | Super Learner (DA Dependency, Long-term) | 9 | 1 | 16 | 2 | 85 | 0.97 (0.92–1.00) | 94 (83–100) | 91 (64–100) | 95 (82–100) | 91 (75–100) | 0.94 | 89.29 | - | - | MCC=0.85 (0.60–1.00) |
|  | Super Learner (DA Dependency, Early-term) | 5 | 1 | 10 | 12 | 85 | 0.80 (0.57–0.94) | 89 (73–100) | 46 (14–86) | 86 (77–95) | 56 (15–100) | 0.87 | 53.57 | - | - | MCC=0.38 (-0.08–0.77) |
|  | Random Forest (DA Dependency, Long-term) |  |  |  |  |  | 0.98 | - | - | - | - | - | - | - | - | MCC=0.93 |
|  | Super Learner (Hyperprolactinemia, Early) | 8 | 8 | 7 | 2 | 76 | 0.69 (0.50–0.83) | 53 (22–78) | 74 (53–94) | 52 (33–76) | 76 (64–88) | 0.52 | 60 | - | - | MCC=0.27 (-0.02–0.57) |
|  | Super Learner (Hyperprolactinemia, Long-term) | 4 | 22 | 2 | 0 | 83 | 0.80 (0.58–0.97) | 17 (0–67) | 95 (80–100) | 23 (0–100) | 93 (88–96) | 0.20 | 21.43 | - | - | MCC=0.11 (-0.12–0.69) |
| (Park et al., 2023) | 1-mm DLR (Reader 1, CSI) |  |  |  |  | 64 | 0.92 (0.823–0.975) | 88.2 | 96.2 |  |  |  |  | 308.9 | 133.5 | ICC(SNR)=0.949 (0.847–0.992), ICC(CNR)=0.924 (0.777–0.998) |
|  | 3-mm (Reader 1, CSI) |  |  |  |  | 64 | 0.88 (0.773–0.951) | 76.5 | 100 |  |  |  |  | 253.9 | 124.2 | ICC(SNR)=0.945 (0.881–0.977), ICC(CNR)=0.923 (0.827–0.972) |
|  | 1-mm DLR (Reader 2, CSI) |  |  |  |  | 64 | 0.91 (0.804–0.967) | 85.3 | 96.2 |  |  |  |  | 308.9 | 133.5 | ICC(SNR)=0.949 (0.847–0.992), ICC(CNR)=0.924 (0.777–0.998) |
|  | 3-mm (Reader 2, CSI) |  |  |  |  | 64 | 0.87 (0.758–0.943) | 97.1 | 76.9 |  |  |  |  | 253.9 | 124.2 | ICC(SNR)=0.945 (0.881–0.977), ICC(CNR)=0.923 (0.827–0.972) |
|  | 1-mm DLR (Reader 1, Residual Tumor) | 58 | 2 | 3 | 1 | 64 | - | 96.7 | 75 | 98.31 | 60 | 0.98 | 95.3 | 308.9 | 133.5 | ICC(SNR)=0.949 (0.847–0.992), ICC(CNR)=0.924 (0.777–0.998) |
|  | 3-mm (Reader 1, Residual Tumor) |  |  |  |  | 64 | - | 96.7 | 75 |  |  |  |  | 253.9 | 124.2 | ICC(SNR)=0.945 (0.881–0.977), ICC(CNR)=0.923 (0.827–0.972) |
|  | 1-mm DLR (Reader 2, Residual Tumor) | 56 | 4 | 3 | 1 | 64 | - | 93.3 | 75 | 98.25 | 42.86 | 0.96 | 92.2 | 308.9 | 133.5 | ICC(SNR)=0.949 (0.847–0.992), ICC(CNR)=0.924 (0.777–0.998) |
|  | 3-mm (Reader 2, Residual Tumor) |  |  |  |  | 64 | - | 93.3 | 75 |  |  |  |  | 253.9 | 124.2 | ICC(SNR)=0.945 (0.881–0.977), ICC(CNR)=0.923 (0.827–0.972) |
| (Wang et al., 2021) | Clinical (AdaBoost, Auto) | 8 | 2 | 16 | 4 | 30 | 0.84 (0.735–0.945) | 75 | 78 | 66.67 | 88.89 | 0.71 | 77 | - | - | - |
|  | Radiomics (KNN, Auto) | 8 | 2 | 17 | 3 | 30 | 0.92 (0.842–0.998) | 80 | 83 | 72.73 | 89.47 | 0.76 | 81.8 | - | - | Dice=0.940 (0.913–0.967) |
|  | Clinical (Linear SVC, Ori) | 7 | 3 | 15 | 5 | 30 | 0.821 (N/A) | 70 | 75 | 58.33 | 83.33 | 0.64 | 74.1 | - | - | - |
|  | Radiomics (MLP, Ori) | 8 | 2 | 16 | 4 | 30 | 0.877 (N/A) | 75 | 80 | 66.67 | 88.89 | 0.71 | 78.3 | - | - | - |
|  | Knosp Grade 0 | 1 | 0 | 31 | 0 | 32 | - | 100 | 100 | 100 | 100 | 1.0 | 100 | - | - | Kappa=0.781 |
|  | Knosp Grade 1 | 2 | 0 | 30 | 0 | 32 | - | 100 | 100 | 100 | 100 | 1.0 | 100 | - | - | Kappa=0.781 |
|  | Knosp Grade 2 | 2 | 0 | 30 | 0 | 32 | - | 100 | 100 | 100 | 100 | 1.0 | 100 | - | - | Kappa=0.781 |
|  | Knosp Grade 3a | 6 | 0 | 26 | 0 | 32 | - | 100 | 100 | 100 | 100 | 1.0 | 100 | - | - | Kappa=0.781 |
|  | Knosp Grade 3b | 4 | 4 | 22 | 2 | 32 | - | 50 | 91.7 | 66.67 | 84.62 | 0.57 | 81.25 | - | - | Kappa=0.781 |
|  | Knosp Grade 4 | 12 | 1 | 19 | 0 | 32 | - | 92.3 | 100 | 100 | 95 | 0.96 | 96.88 | - | - | Kappa=0.781 |
| (Zoli et al., 2020) | KNN (GTR, Test) | 26 | 1 | 3 | 0 | 30 | 0.988 | 96.4 | 100 | 100 | 75 | 0.982 | 96.8 | - | - | Brier=0.035 |
|  | SVM (Early Remission, Test) | 26 | 0 | 4 | 0 | 30 | 1.00 | 100 | 100 | 100 | 100 | 1.00 | 100 | - | - | Brier=0.097 |
|  | GBM (Long-term Remission, Test) | 26 | 1 | 1 | 2 | 30 | 0.783 | 95.7 | 37.5 | 81.5 | 75 | 0.880 | 80.7 | - | - | Brier=0.151 |
|  | KNN (GTR, Training) | 107 | 3 | 10 | 1 | 121 | 0.993 | 97 | 88.9 | 99 | 72.7 | 0.980 | 96.3 | - | - | - |
|  | SVM (Early Remission, Training) | 94 | 12 | 2 | 13 | 121 | 0.681 | 89.1 | 14.3 | 96.1 | 5.3 | 0.925 | 86.1 | - | - | - |
|  | GBM (Long-term Remission, Training) | 91 | 19 | 4 | 7 | 121 | 0.719 | 82.4 | 35.4 | 82.4 | 40.5 | 0.838 | 74.3 | - | - | - |
| (Zhang et al., 2021b) | Stacking |  |  |  |  |  | 0.743 (0.677–0.806) | 80.4 | 58.9 | 79.2 | 60 | 0.798 | 74.6 | - | - | MCC=0.399 |
|  | GBDT |  |  |  |  |  | 0.734 (0.669–0.796) | - | - | - | - | - | 73.2 | - | - | - |
|  | Random Forest |  |  |  |  |  | 0.726 (0.656–0.792) | - | - | - | - | - | 74.6 | - | - | - |
|  | XGBoost |  |  |  |  |  | 0.712 (0.645–0.776) | - | - | - | - | - | 73.2 | - | - | - |
|  | Logistic Regression |  |  |  |  |  | 0.701 (0.630–0.767) | - | - | - | - | - | 73.2 | - | - | - |
|  | MLP |  |  |  |  |  | 0.700 (0.629–0.766) | - | - | - | - | - | 73.2 | - | - | - |
|  | AdaBoost |  |  |  |  |  | 0.699 (0.632–0.764) | - | - | - | - | - | 73.7 | - | - | - |
|  | Naïve Bayes |  |  |  |  |  | 0.681 (0.614–0.744) | - | - | - | - | - | 68.4 | - | - | - |
|  | Decision Tree |  |  |  |  |  | 0.664 (0.598–0.726) | - | - | - | - | - | 74.2 | - | - | - |
| (Shu et al., 2022) | LR (Structured + Conclusions) |  |  |  |  |  | 0.793 (0.689–0.897) | - | - | - | - | - | - | - | - | - |
|  | LR (Chief Complaint) |  |  |  |  |  | 0.777 (0.709–0.845) | - | - | - | - | - | - | - | - | - |
|  | LR (HPI) |  |  |  |  |  | 0.737 (0.624–0.850) | - | - | - | - | - | - | - | - | - |
|  | MLP (Structured) |  |  |  |  |  | 0.759 (0.633–0.885) | - | - | - | - | - | - | - | - | - |
|  | MLP (HPI + Conclusions) |  |  |  |  |  | 0.737 (0.677–0.796) | - | - | - | - | - | - | - | - | - |
|  | SVM (Structured) |  |  |  |  |  | 0.733 (0.612–0.845) | - | - | - | - | - | - | - | - | - |
|  | SVM (Chief Complaint + Conclusions) |  |  |  |  |  | 0.722 (0.628–0.816) | - | - | - | - | - | - | - | - | - |
|  | RF (Structured) |  |  |  |  |  | 0.678 (0.544–0.812) | - | - | - | - | - | - | - | - | - |
|  | RF (Chief Complaint) |  |  |  |  |  | 0.686 (0.610–0.756) | - | - | - | - | - | - | - | - | - |
|  | RF (Cautions) |  |  |  |  |  | 0.468 (0.317–0.619) | - | - | - | - | - | - | - | - | - |
| (Staartjes et al., 2018) | Deep Neural Network | 89 | 6 | 40 | 5 | 140 | 0.962 (0.960–0.963) | 93.7 (93.1–94.3) | 88.9 (88.2–89.5) | 88.6 (88.1–89.2) | 93.9 (93.2–94.5) | 0.908 | 90.9 (90.5–91.3) | - | - | - |
|  | Logistic Regression | 77 | 18 | 37 | 8 | 140 | 0.860 (0.845–0.875) | 80.9 (79.4–82.5) | 83.3 (81.4–85.2) | 83.1 (81.3–84.9) | 81.4 (79.6–83.2) | 0.817 | 82 (81–83.1) | - | - | - |
|  | Knosp Classification | 88 | 7 | 32 | 13 | 140 | 0.868 (N/A) | 92.2 | 70 | 75.5 | 89.9 | 0.830 | 81.1 | - | - | - |
| (Niu et al., 2019) | Clinico-radiological (Training) | 36 | 11 | 38 | 12 | 97 | 0.846 (0.831–0.861) | 76.5 | 76.1 | 75 | 77.55 | 0.76 | 76.3 | - | - | - |
|  | Clinico-radiological (Test Set) | 29 | 6 | 43 | 19 | 97 | 0.828 (0.812–0.844) | 82.3 | 68.6 | 60.42 | 87.76 | 0.70 | 77.3 | - | - | - |
|  | CE-T1 (Training) | 40 | 7 | 33 | 17 | 97 | 0.852 (0.837–0.868) | 85.1 | 66 | 70.18 | 82.5 | 0.77 | 75.3 | - | - | - |
|  | CE-T1 (Test Set) | 28 | 7 | 50 | 12 | 97 | 0.826 (0.804–0.844) | 80 | 80.7 | 70 | 87.72 | 0.75 | 80.4 | - | - | - |
|  | T2 (Training) | 38 | 9 | 31 | 19 | 97 | 0.768 (0.748–0.787) | 80.9 | 62 | 66.67 | 77.5 | 0.73 | 71.1 | - | - | - |
|  | T2 (Test Set) | 22 | 13 | 44 | 18 | 97 | 0.733 (0.712–0.754) | 62.9 | 71 | 55 | 77.19 | 0.59 | 68 | - | - | - |
|  | CE-T1+T2 (Training) | 40 | 7 | 33 | 17 | 97 | 0.869 (0.855–0.884) | 85.1 | 66 | 70.18 | 82.5 | 0.77 | 75.3 | - | - | - |
|  | CE-T1+T2 (Test Set) | 27 | 8 | 49 | 13 | 97 | 0.803 (0.784–0.821) | 77.1 | 79 | 67.5 | 85.96 | 0.72 | 79.1 | - | - | - |
|  | Nomogram (Training) | 44 | 3 | 35 | 15 | 97 | 0.899 (0.887–0.911) | 93.6 | 70 | 74.58 | 92.11 | 0.83 | 81.4 | - | - | - |
|  | Nomogram (Test Set) | 30 | 5 | 47 | 15 | 97 | 0.871 (0.857–0.885) | 85.7 | 75.8 | 66.67 | 90.38 | 0.75 | 79.4 | - | - | - |
| (McKevitt et al., 2023) | Pit-SCHEME Score | 224 | 37 | 92 | 39 | 392 | 0.858 (0.820–0.895) | 85.8 | 70.2 | 85.17 | 71.32 | 0.85 | 80.6 | - | - | LR+=2.88 |
|  | LDA (Multivariate) | 35 | 21 | 20 | 8 | 84 | 0.78 (N/A) | 62 (41–80) | 71 (57–83) | 81.40 | 48.78 | 0.70 | 68 (56–78) | - | - | - |
|  | CART (Multivariate) | 32 | 24 | 22 | 6 | 84 | 0.70 (N/A) | 58 (37–77) | 79 (65–89) | 84.21 | 47.83 | 0.69 | 72 (60–81) | - | - | - |
|  | kNN (Multivariate) | 24 | 32 | 22 | 6 | 84 | 0.83 (N/A) | 42 (23–63) | 79 (65–89) | 80 | 40.74 | 0.55 | 63 (51–74) | - | - | - |
|  | SVM (Multivariate) | 28 | 28 | 19 | 9 | 84 | - | 50 (30–70) | 69 (55–81) | 75.68 | 40.43 | 0.60 | 62 (51–74) | - | - | - |
|  | RF (Multivariate) | 32 | 24 | 22 | 6 | 84 | 0.88 (N/A) | 58 (37–77) | 79 (65–89) | 84.21 | 47.83 | 0.69 | 72 (60–81) | - | - | - |
|  | Naïve Bayes (Multivariate) | 36 | 20 | 18 | 10 | 84 | 0.77 (N/A) | 65 (44–83) | 65 (51–78) | 78.26 | 47.37 | 0.71 | 65 (54–76) | - | - | - |
|  | LDA (Multivariate + Pit-SCHEME) | 41 | 15 | 24 | 4 | 84 | 0.90 (N/A) | 74 (54–89) | 85 (72–94) | 91.11 | 61.54 | 0.82 | 81 (71–89) | - | - | p=0.0002 |
|  | CART (Multivariate + Pit-SCHEME) | 31 | 25 | 26 | 2 | 84 | 0.77 (N/A) | 56 (35–75) | 92 (80–98) | 93.94 | 50.98 | 0.70 | 79 (68–87) | - | - | p=0.006 |
|  | kNN (Multivariate + Pit-SCHEME) | 31 | 25 | 25 | 3 | 84 | 0.88 (N/A) | 56 (35–75) | 88 (75–95) | 91.18 | 50 | 0.70 | 75 (63–84) | - | - | - |
|  | SVM (Multivariate + Pit-SCHEME) | 31 | 25 | 26 | 2 | 84 | - | 56 (35–75) | 92 (80–98) | 93.94 | 50.98 | 0.70 | 79 (68–87) | - | - | p<0.0001 |
|  | RF (Multivariate + Pit-SCHEME) | 44 | 12 | 26 | 2 | 84 | 0.97 (N/A) | 78 (58–91) | 92 (80–98) | 95.65 | 68.42 | 0.86 | 85 (75–92) | - | - | - |
|  | Naïve Bayes (Multivariate + Pit-SCHEME) | 35 | 21 | 26 | 2 | 84 | 0.81 (N/A) | 63 (42–81) | 94 (83–99) | 94.59 | 55.32 | 0.76 | 83 (72–90) | - | - | - |
| (Fang et al., 2024) | CNN (External Test) | 16 | 5 | 58 | 3 | 82 | 0.92 (0.88–0.96) | 77 | 95 | 87 | 89 | 0.82 | 89 | - | - | DOR=63.61 |
|  | Diameter (23.45 mm) | 20 | 1 | 34 | 27 | 82 | 0.75 (N/A) | 94 | 55 | 36 | 97 | 0.52 | 63 | - | - | DOR=19.86 |
|  | Length (23.95 mm) | 18 | 3 | 44 | 17 | 82 | 0.80 (N/A) | 88 | 72 | 45 | 96 | 0.60 | 76 | - | - | DOR=19.58 |
|  | Knosp (0–1/2–4) | 20 | 1 | 24 | 37 | 82 | 0.70 (N/A) | 97 | 40 | 31 | 98 | 0.47 | 52 | - | - | DOR=23.48 |
|  | Knosp (0–2/3A–4) | 18 | 3 | 46 | 15 | 82 | 0.82 (N/A) | 86 | 75 | 48 | 95 | 0.62 | 78 | - | - | DOR=18.60 |
|  | Knosp (0–3A/3B–4) | 9 | 12 | 60 | 1 | 82 | 0.70 (N/A) | 42 | 98 | 83 | 86 | 0.56 | 87 | - | - | DOR=30.71 |
| (Ishimoto et al., 2024) | CUBE with DLR (Tumors, Radiologist A) | - | - | - | - | 24 | - | - | - | - | - | - | - | 11.6 (14.4–9.8) | 13.7 (17.4–12.0) – 35.2 (36.7–26.2) | Depiction=3 (2.9–2.6), Kappa=0.75 |
|  | CUBE with DLR (Tumors, Radiologist B) | - | - | - | - | 24 | - | - | - | - | - | - | - | 11.6 (14.4–9.8) | 13.7 (17.4–12.0) – 35.2 (36.7–26.2) | Depiction=3 (3.0–2.7), Kappa=0.75 |
|  | CUBE with DLR (Boundary) | - | - | - | - | 13 | - | - | - | - | - | - | - | 11.6 (14.4–9.8) | 13.7 (17.4–12.0) – 35.2 (36.7–26.2) | Depiction=2 (2.1–1.8), Kappa=0.81 |
|  | CUBE without DLR (Tumors, Radiologist A) | - | - | - | - | 24 | - | - | - | - | - | - | - | 6.6 (8.9–6.4) | 3.8 (7.2–3.0) – 8.8 (13.6–7.5) | Depiction=2 (2.3–1.9), Kappa=0.20 |
|  | CUBE without DLR (Tumors, Radiologist B) | - | - | - | - | 24 | - | - | - | - | - | - | - | 6.6 (8.9–6.4) | 3.8 (7.2–3.0) – 8.8 (13.6–7.5) | Depiction=2 (1.9–1.6), Kappa=0.20 |
|  | CUBE without DLR (Boundary) | - | - | - | - | 13 | - | - | - | - | - | - | - | 6.6 (8.9–6.4) | 3.8 (7.2–3.0) – 8.8 (13.6–7.5) | Depiction=1 (1.7–0.9), Kappa=0.45 |
|  | 1-mm 2D T1WI with DLR (Tumors, Radiologist A) | - | - | - | - | 24 | - | - | - | - | - | - | - | 10.5 (13.1–9.5) | 16.4 (21.4–14.6) – 34.2 (38.8–29.5) | Depiction=2 (2.1–1.6), Kappa=0.41 |
|  | 1-mm 2D T1WI with DLR (Tumors, Radiologist B) | - | - | - | - | 24 | - | - | - | - | - | - | - | 10.5 (13.1–9.5) | 16.4 (21.4–14.6) – 34.2 (38.8–29.5) | Depiction=2 (2.5–1.9), Kappa=0.41 |
|  | 1-mm 2D T1WI with DLR (Boundary) | - | - | - | - | 13 | - | - | - | - | - | - | - | 10.5 (13.1–9.5) | 16.4 (21.4–14.6) – 34.2 (38.8–29.5) | Depiction=1 (1.8–0.9), Kappa=0.35 |
|  | SPGR (Tumors, Radiologist A) | - | - | - | - | 24 | - | - | - | - | - | - | - | 9.3 (10.8–8.4) | 6.3 (9.0–5.7) – 16.0 (19.3–13.8) | Depiction=2 (2.0–1.6), Kappa=0.60 |
|  | SPGR (Tumors, Radiologist B) | - | - | - | - | 24 | - | - | - | - | - | - | - | 9.3 (10.8–8.4) | 6.3 (9.0–5.7) – 16.0 (19.3–13.8) | Depiction=2 (2.1–1.7), Kappa=0.60 |
|  | SPGR (Boundary) | - | - | - | - | 13 | - | - | - | - | - | - | - | 9.3 (10.8–8.4) | 6.3 (9.0–5.7) – 16.0 (19.3–13.8) | Depiction=1 (1.2–0.8), Kappa=0.64 |
| (Shu et al., 2022) | ceT1WI (Validation) | - | - | - | - | 52 | - | 86.85 | 85.97 | - | - | - | 87.39 | - | - | - |
|  | T2WI (Validation) | - | - | - | - | 52 | - | 89.65 | 88.57 | - | - | - | 89.4 | - | - | - |
|  | Multimodal (Validation) | - | - | - | - | 52 | - | 89.98 | 86.82 | - | - | - | 89.18 | - | - | - |
|  | ceT1WI (Clinical, 1 slice) | - | - | - | - | 27 | - | - | - | - | - | - | 62.96 | - | - | - |
|  | ceT1WI (Clinical, 3 slices) | - | - | - | - | 27 | - | - | - | - | - | - | 74.07 | - | - | - |
|  | ceT1WI (Clinical, 5 slices) | - | - | - | - | 27 | - | - | - | - | - | - | 74.07 | - | - | - |
|  | T2WI (Clinical, 1 slice) | - | - | - | - | 27 | - | - | - | - | - | - | 77.78 | - | - | - |
|  | T2WI (Clinical, 3 slices) | - | - | - | - | 27 | - | - | - | - | - | - | 77.78 | - | - | - |
|  | T2WI (Clinical, 5 slices) | - | - | - | - | 27 | - | - | - | - | - | - | 74.07 | - | - | - |
|  | Multimodal (Clinical, 1 slice) | - | - | - | - | 27 | - | - | - | - | - | - | 70.37 | - | - | - |
|  | Multimodal (Clinical, 3 slices) | - | - | - | - | 27 | - | - | - | - | - | - | 77.78 | - | - | - |
|  | Multimodal (Clinical, 5 slices) | - | - | - | - | 27 | - | - | - | - | - | - | 77.78 | - | - | - |
| (Osorio et al., 2025) | Preop Tumor Volume (Threshold: 1.51 cm³) | - | - | - | - | 80 | 0.691 (N/A) | 42.9 | 94.7 | - | - | - | - | - | - | p=0.003, Threshold=1.51 cm³ |
|  | Max Tumor Dimension (Threshold: 1.55 cm) | - | - | - | - | 80 | 0.684 (N/A) | 64.4 | 75.0 | - | - | - | - | - | - | p=0.005, Threshold=1.55 cm |
|  | Preop IGF-1 Level (Threshold: 718.50 ng/ml) | - | - | - | - | 80 | 0.736 (N/A) | 58.1 | 100.0 | - | - | - | - | - | - | p=0.002, Threshold=718.50 ng/ml |
|  | Postop GH Level (GTR, Threshold: 1.15 ng/ml) | - | - | - | - | 55 | 0.837 (N/A) | 61.7 | 100.0 | - | - | - | - | - | - | p<0.001, Threshold=1.15 ng/ml |
| (Rui et al., 2025) | MTMAU-Net (All Grades, Training) | 156 | 62 | 562 | 36 | 816 | 0.89 (N/A) | 71.43 | 94.02 | 81.25 | 90.06 | 0.76 | 88.05 | - | - | Dice=90.84 ± 0.55%, HD95=2.13 ± 0.37 mm |
|  | MTMAU-Net (Knosp 0–2, Training) | 139 | 79 | 552 | 46 | 816 | - | 63.64 | 92.23 | 75.14 | 87.48 | 0.69 | 87.20 | - | - | - |
|  | MTMAU-Net (Knosp 3, Training) | 153 | 65 | 555 | 43 | 816 | - | 70.00 | 92.86 | 78.06 | 89.52 | 0.74 | 83.33 | - | - | - |
|  | MTMAU-Net (Knosp 4, Training) | 218 | 0 | 598 | 0 | 816 | - | 100.00 | 100.00 | 100.00 | 100.00 | 1.00 | 100.00 | - | - | - |
|  | Knosp Grading (All Grades, Training) | 155 | 63 | 560 | 38 | 816 | - | 71 | 93.65 | 80.31 | 89.89 | 0.75 | 87.68 | - | - | - |
|  | Knosp Grading (Knosp 0–2, Training) | 155 | 63 | 560 | 38 | 816 | - | 71 | 93.65 | 80.31 | 89.89 | 0.75 | 87.68 | - | - | - |
|  | Knosp Grading (Knosp 3, Training) | 120 | 98 | 349 | 249 | 816 | - | 55 | 58.44 | 32.52 | 78.08 | 0.41 | 57.62 | - | - | - |
|  | Knosp Grading (Knosp 4, Training) | 218 | 0 | 598 | 0 | 816 | - | 100 | 100 | 100.00 | 100.00 | 1.00 | 100.00 | - | - | - |
|  | MTMAU-Net (Validation) | 80 | 15 | 13 | 2 | 110 | 0.87 (N/A) | 84 | 86.67 | 97.56 | 46.43 | 0.90 | 84.55 | - | - | Dice=83.71 ± 5.93% |
|  | 2D-UNet (Segmentation, Training) | - | - | - | - | 816 | - | - | - | - | - | - | - | - | - | Dice=84.17 ± 0.96%, HD95=7.17 ± 0.61 mm |
|  | 3D-UNet (Segmentation, Training) | - | - | - | - | 816 | - | - | - | - | - | - | - | - | - | Dice=85.68 ± 0.65%, HD95=6.03 ± 0.46 mm |
|  | V-Net (Segmentation, Training) | - | - | - | - | 816 | - | - | - | - | - | - | - | - | - | Dice=85.91 ± 0.64%, HD95=5.96 ± 0.25 mm |
|  | nnU-Net (Segmentation, Training) | - | - | - | - | 816 | - | - | - | - | - | GMT | - | - | - | Dice=90.79 ± 0.55%, HD95=2.15 ± 0.38 mm |
|  | UNETR (Segmentation, Training) | - | - | - | - | 816 | - | - | - | - | - | - | - | - | - | Dice=89.14 ± 0.58%, HD95=2.57 ± 0.38 mm |
|  | 2D-ResNet18 (Classification, Training) | 104 | 114 | 537 | 61 | 816 | 0.6961 (N/A) | 47.62 | 89.74 | 63.03 | 82.49 | 0.54 | 78.62 | - | - | - |
|  | 2D-ResNet34 (Classification, Training) | 47 | 171 | 583 | 15 | 816 | 0.5782 (N/A) | 21.42 | 97.44 | 75.81 | 77.32 | 0.33 | 77.36 | - | - | - |
|  | 2D-ResNet50 (Classification, Training) | 78 | 140 | 557 | 41 | 816 | 0.7443 (N/A) | 35.71 | 93.16 | 65.55 | 79.91 | 0.46 | 77.99 | - | - | - |
|  | 2D-DenseNet121 (Classification, Training) | 130 | 88 | 516 | 82 | 816 | 0.7503 (N/A) | 59.52 | 86.32 | 61.32 | 85.43 | 0.60 | 79.24 | - | - | - |
|  | 2D-SENet50 (Classification, Training) | 78 | 140 | 537 | 61 | 816 | 0.6144 (N/A) | 35.71 | 89.74 | 56.12 | 79.32 | 0.44 | 75.47 | - | - | - |
|  | 3D-ResNet18 (Classification, Training) | 99 | 119 | 567 | 31 | 816 | 0.7526 (N/A) | 45.24 | 94.87 | 76.15 | 82.65 | 0.57 | 81.76 | - | - | - |
|  | 3D-ResNet34 (Classification, Training) | 93 | 125 | 578 | 20 | 816 | 0.7159 (N/A) | 42.86 | 96.58 | 82.30 | 82.22 | 0.56 | 82.39 | - | - | - |
|  | 3D-ResNet50 (Classification, Training) | 83 | 135 | 562 | 36 | 816 | 0.6763 (N/A) | 38.10 | 94.02 | 69.75 | 80.66 | 0.49 | 79.25 | - | - | - |
|  | MTMAU-Net (Only Segmentation, Training) | - | - | - | - | 816 | - | - | - | - | - | - | - | - | - | Dice=89.94 ± 0.58%, HD95=2.44 ± 0.38 mm |
|  | MTMAU-Net (Only Classification, Training) | 114 | 104 | 542 | 56 | 816 | 0.8089 (N/A) | 52.38 | 90.60 | 67.06 | 83.90 | 0.59 | 80.50 | - | - | - |


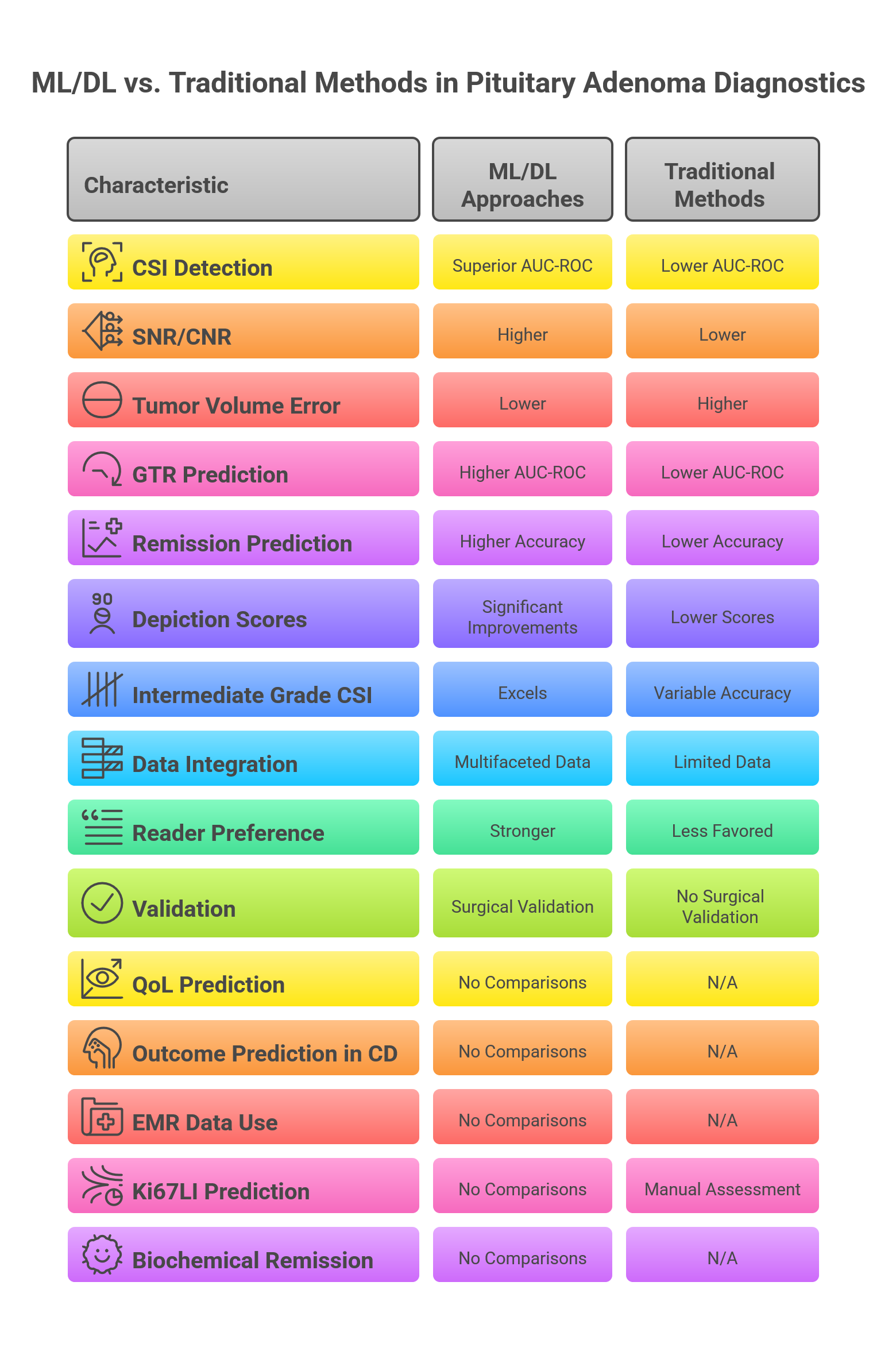


**Supplemental Figure 1**. Comparative performance of ML and DL models against traditional methods.


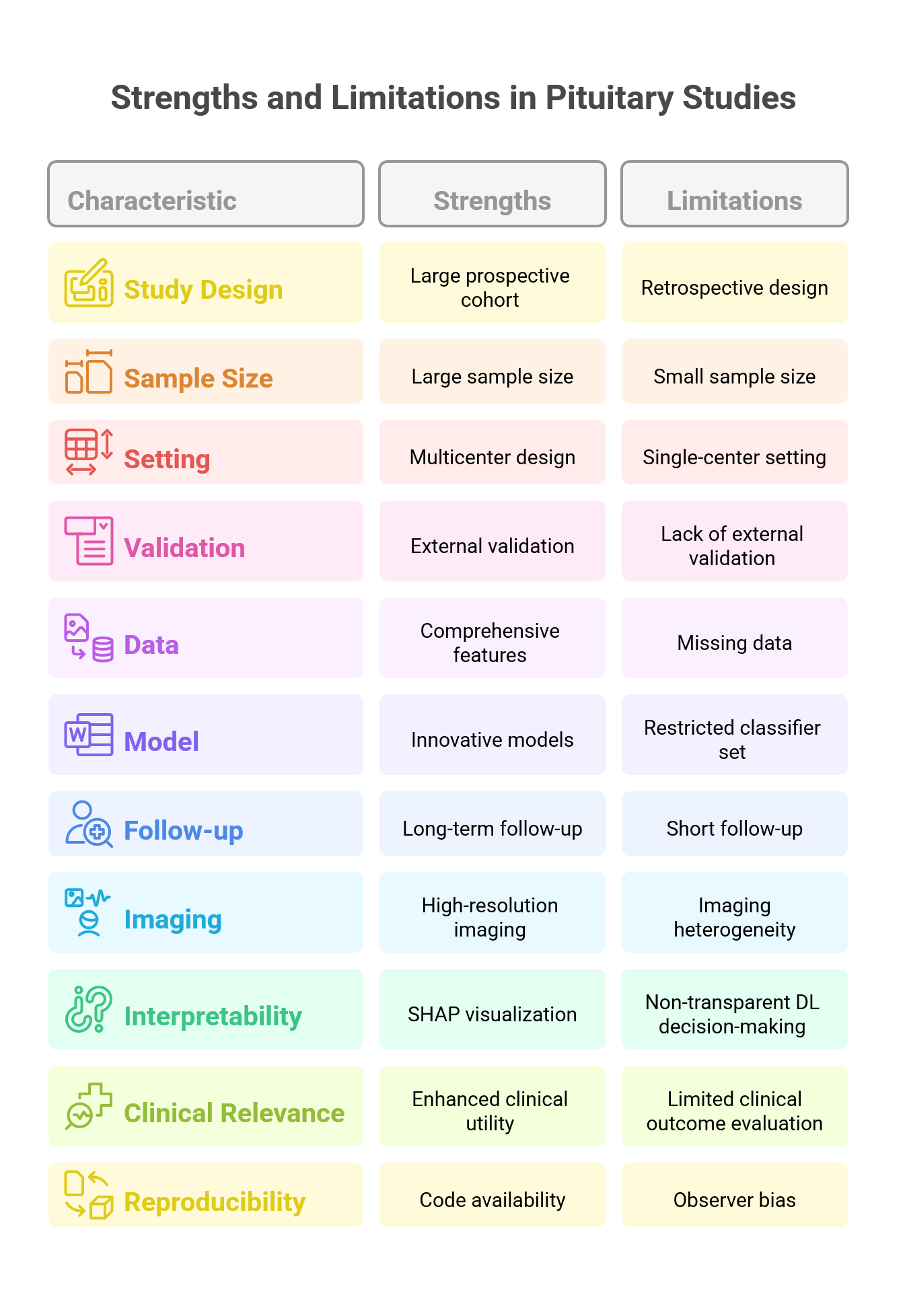
**Supplemental Figure 2.** Visual summary of strengths (e.g., large samples, multicenter designs, innovative models) and limitations.
